# Current advance of nanotechnology in diagnosis and treatment for malignant tumors

**DOI:** 10.1038/s41392-024-01889-y

**Published:** 2024-08-12

**Authors:** Bilan Wang, Shiqi Hu, Yan Teng, Junli Chen, Haoyuan Wang, Yezhen Xu, Kaiyu Wang, Jianguo Xu, Yongzhong Cheng, Xiang Gao

**Affiliations:** 1 Department of Pharmacy, Evidence-based Pharmacy Center, Children’s Medicine Key Laboratory of Sichuan Province, West China Second University Hospital, Sichuan University, Chengdu, 610041 China; 2 Key Laboratory of Birth Defects and Related Diseases of Women and Children, Ministry of Education, West China Second University Hospital, Sichuan University, Chengdu, 610041 P.R. China; 3 Department of Gynecology and Obstetrics, Development and Related Diseases of Women and Children Key Laboratory of Sichuan Province, Key Laboratory of Birth Defects and Related Diseases of Women and Children, Ministry of Education, West China Second University Hospital, Sichuan University, Chengdu, 610041 P.R. China; 4 Institute of Laboratory Medicine, Sichuan Provincial People’s Hospital, School of Medicine, University of Electronic Science and Technology of China, Chengdu, 610072 P.R. China; 5 West China School of Basic Medical Sciences & Forensic Medicine, Sichuan University, Chengdu, 610041 China; 6 Department of Neurosurgery and Institute of Neurosurgery, State Key Laboratory of Biotherapy and Cancer Center, West China Hospital, West China Medical School, Sichuan University and Collaborative Innovation Center for Biotherapy, Chengdu, 610041 China

**Keywords:** Drug development, Translational research

## Abstract

Cancer remains a significant risk to human health. Nanomedicine is a new multidisciplinary field that is garnering a lot of interest and investigation. Nanomedicine shows great potential for cancer diagnosis and treatment. Specifically engineered nanoparticles can be employed as contrast agents in cancer diagnostics to enable high sensitivity and high-resolution tumor detection by imaging examinations. Novel approaches for tumor labeling and detection are also made possible by the use of nanoprobes and nanobiosensors. The achievement of targeted medication delivery in cancer therapy can be accomplished through the rational design and manufacture of nanodrug carriers. Nanoparticles have the capability to effectively transport medications or gene fragments to tumor tissues via passive or active targeting processes, thus enhancing treatment outcomes while minimizing harm to healthy tissues. Simultaneously, nanoparticles can be employed in the context of radiation sensitization and photothermal therapy to enhance the therapeutic efficacy of malignant tumors. This review presents a literature overview and summary of how nanotechnology is used in the diagnosis and treatment of malignant tumors. According to oncological diseases originating from different systems of the body and combining the pathophysiological features of cancers at different sites, we review the most recent developments in nanotechnology applications. Finally, we briefly discuss the prospects and challenges of nanotechnology in cancer.

## Introduction

Cancer is one of the leading causes of death globally, with an approximate incidence of 19.3 million newly diagnosed cases and about 10.0 million fatalities annually.^1,2^ Due to its ever-growing incidence and mortality rate, it has become one of the highest challenges for a longer life expectancy worldwide. Although in developed countries like the United States, general cancer incidence and mortality rates have been gradually decreasing in the past decade owing to new research on tumor mechanisms, improved diagnostic tools, and new therapeutic strategies, the burden of cancer remains a major challenge in developing as well as developed economies.^2–7^ It is in urgent demand for more ground-breaking innovations and ever-improving tactics, together with a better understanding and utilization of existing cancer management strategies.

Common treatments, including surgery, radiotherapy, and chemotherapy, have been applied to most patients with malignant tumors, either in combination or separately. Although surgery is regarded as an irreplaceable treatment for most solid localized tumors, systematic treatment is required as a supplement upon the occurrence of metastasis.^8–14^ Radiation therapy is used in more than half of patients with cancer and is one of the most effective treatment modalities,^15–21^ but it remains a huge challenge to alleviate short- and long-term toxicity.^15,22–26^ Traditional chemotherapy is also facing great challenges due to its inherent properties, such as instability, insolubility, drug resistance, and prominent tissue damage.^27–34^ Also, the traditional systematic administration of drugs has put all somatic cells at risk of toxicity. However, there are ways of reducing side effects and avoiding the drawbacks of chemotherapy by enclosing drugs inside a tiny compartment, absorbing the drug into well-designed pores or mediums, providing a relatively stabilized microenvironment, enabling active interactions within the body with the assistance of biomimicking membranes, and releasing drugs after being transported to the desired sites. This is what nanotechnology is attempting to do.

**Fig. 1:**
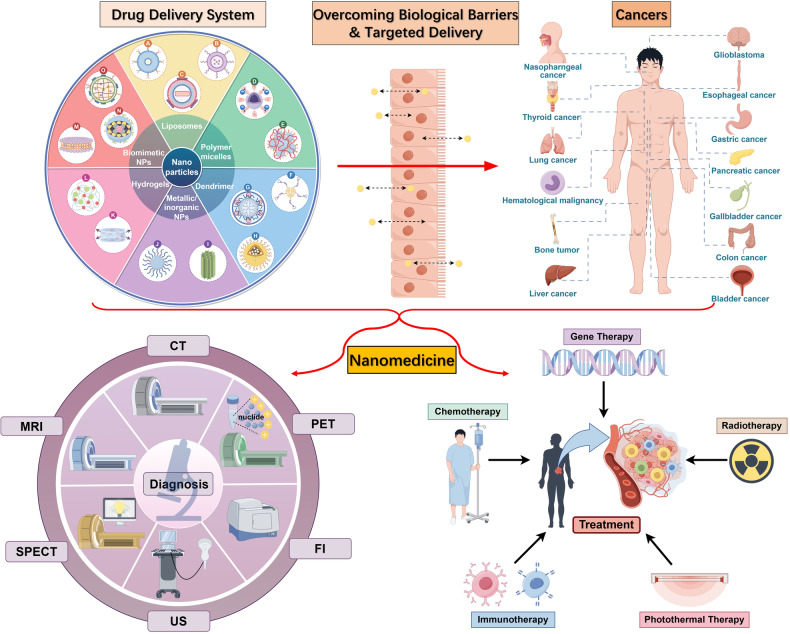
Nanomedicine in cancer diagnosis and treatment. CT computed tomography, MRI magnetic resonance imaging, PET positron emission tomography, SPECT single-photon emission tomography, US ultrasound, FI fluorescence imaging. (Drawn by Figdraw)

Nanomedicine, after being brought forward conceptually, has been investigated as being used in a large number of diseases,^35–41^ especially in the efficient transportation of anti-tumor drugs, diagnosis, and imaging, giving credit to its prominent physiochemical and structural characteristics (Fig. 1).^42–46^ In the past years, nanomedicine has undergone a long history of evolution, namely, from non-targeting to targeting, from simple materials to mixed systems, and from single to combined technologies. The combination of nanotechnology and conventional tumor therapy can not only enhance the properties of chemoradiotherapy drugs but also reduce the incidence of poisoning and other side effects.^39,47–58^ Regulatory authorities have authorized several therapeutic nanoparticle (NP) platforms, such as liposomes, albumin, and polymeric micelles, for cancer treatment. These NPs can rapidly cross the human biological barriers, even in a targeted manner^59–61^ and continuously release the content to maintain the appropriate blood concentration of the drug.^62,63^ A growing interest in manufacturing new formulations inside the NPs can avoid the disadvantages of both traditional treatment and nanotechnology and promote preclinical and clinical efficacy.^64–66^

Meanwhile, along with the advantages nanomedicines are offering, the challenges ahead are also worth mentioning. There are mainly two unresolved issues in the nanomedicine area. Enhanced permeability and retention (EPR), a widely recognized routine of nanomedicine, has provided limited improvement in the survival outcomes of patients, even though EPR has been confirmed to decrease the risk of adverse effects and enhance efficacy during the preclinical trial and in the animal model.^67–72^ On the contrary, immune-checkpoint inhibition (ICI), which can inhibit the immune-checkpoint molecule of cancer, improves the survival outcome of patients with cancer considerably.^73,74^ However, the incidence rate of adverse effects in ICI is higher than that of EPR, and only <13% of patients can benefit after the treatment.^75^

This review focuses on summarizing nanomedicines of all types and their diagnostic and therapeutic use in the treatment of various types of cancer. Firstly, the morphological characteristics, uses, and recent advances of each type of nanomedicine are explained, including polymeric nanoparticles, liposomes, micelles, hydrogels, exosomes and other extracellular vesicles, natural membrane nanoparticles, viruses, and inorganic nanoparticles. Secondly, the applications of nanomedicine to malignant tumors are systematically explained, including digestive system, lung (respiratory system), intracranial, hematological, genitourinary, skeletal, skin, and thyroid cancers. The advantages and disadvantages of nanomedicine in the treatment of these tumors are then outlined. Finally, we discuss the prospects and challenges for nanotechnology in malignant tumors (Tables 1–3).

**Table 1:** Features of different nanoparticles

Types of NPs		Advantages	Disadvantages
Polymeric NPs	Natural polymers	Biocompatibility and degradability, Excellent transmucosal capability	Low stability
	Synthetic polymers	High stability, bioavailability and loading capacity	Tissue accumulation, Low degradability, Unfavorable side effects
Liposomes		Excellent biocompatibility, Wide adaptability to drugs, Reduced adverse effects, avoiding bio-clearance of agents	Low storage of lipophilic molecules, opsonization, immunogenicity and instability, High costs
Micelles		Alleviating toxicity, Enabling transportation of lipophilic drugs, Reducing the uptake by macrophages, Prolonging the circulation time, Targeting drug release	Instability in vivo, Low solubility of small size particles, Low drug loading capacity
Hydrogels		High biocompatibility, High drug loading capacity, Controllable and adjustable of drug release, Highly adjustable chemical and physical properties	Fast clearance, Off-target accumulation
Extracellular vesicles		Targeted drug delivery, High biocompatibility, Loading a large variety of cargos	Difficult for isolation, purification and differential recognition, Abnormal accumulations in the liver
Natural membrane-coated NPs		Prolonging the blood circulation time, Evading clearance from immune system, Boosting therapeutic effects, Improving target capabilities	Complexity of scale-up manufacturing, Accumulating in livers or reticuloendothelial systems, Difficulties with purification and storage
Virus		Outstanding targeting capability, Potential in immunotherapy or vaccination strategies	Lacking of clinical translation
Inorganic NPs	Mesoporous silica	Easy functionalization and surface modification, High capacity of storing drugs, Simple production process	High costs
	Gold NPs	Low toxicity, High stability, Simple synthesis, Bioconjugation of desired molecules, Efficient light-to-heat conversion	Prone to accumulating in tissue and toxicity potential, Unsatisfactory clinic treatment outcomes
	Carbon nanomaterials	Excellent optical properties, Thermal conductivity, Electrical conductivity, Chemical stability, Functionalization	Potential problems of biosafety

**Table 2:** Approved nanomedicine for cancer

Therapeutic type	Product name	Therapeutic agent	Nanocarrier	Administration route	Indications	First approval
Chemotherapy	Doxil	Doxorubicin	PEGylated liposomes	iv.	Kaposi sarcoma, breast cancer, ovarian cancer, multiple myeloma	USA 1995
Chemotherapy	Caelyx	Doxorubicin	PEGylated liposome	iv.	Metastatic breast, Kaposi’s sarcoma, multiple myeloma	USA 1996
Chemotherapy	DaunoXome	Daunorubicin	Liposomes	iv.	Kaposi sarcoma	USA 1996
Chemotherapy	Myocet	Doxorubicin	Liposomes	iv.	Metastatic breast cancer	USA 1996
Chemotherapy	DepoCyt	Cytarabine	Liposomes	iv.	Lymphomatous meningitis	USA 1999
Chemotherapy	Eligard	Leuprolide	Polymeric micelles	iv.	Prostate cancer	USA 2002
Chemotherapy	Lipusu	Paclitaxel	Liposomes	iv.	Ovarian cancer, metastatic gastric cancer	China 2003
Chemotherapy	Oncaspar	Pegaspargase	Polymeric micelles	iv.	Acute lymphoblastic leukemia	USA 1994
Chemotherapy	MEPACT	Mifamurtide	Liposomes	iv.	Osteosarcoma	Europe 2009
Chemotherapy	Ameluz	5-aminolevulinic acid	Liposomes	it.	Nodular basal cell carcinoma	USA 2011
Chemotherapy	Marqibo	Vincristine sulfate	Liposomes	iv.	Philadelphia chromosome-negative acute lymphoblastic leukemia	USA 2012
Chemotherapy	Lipodox	Doxorubicin hydrochloride	Liposome	iv.	Kaposi’s sarcoma, ovarian cancer, multiple myeloma, metastatic pancreatic cancer	Chnia 2013
Chemotherapy	Kadcyla	DM1(or Emtansine)	Protein	iv.	Breast cancer	USA 2013
Chemotherapy	Paclical	Paclitaxel	polymeric nanoparticle-based	iv.	Pancreatic cancer, metastatic breast cancer	USA 2015
Chemotherapy	Onivyde	Irinotecan	PEGylated liposomes	iv.	Metastatic pancreatic cancer	USA 2015
Chemotherapy	VYXEOS	Cytarabine/daunorubicin (5∶1)	Liposomes	iv.	Acute myeloid leukemia	USA 2017
Chemotherapy	Pazenir	Paclitaxel	Micelle	iv.	Metastatic breast cancer, metastatic pancreatic cancer, non-small cell lung cancer	USA 2019
Chemotherapy	Apealea	Paclitaxel	Nanocrystal	iv.	Ovarian cancer, peritoneal cancer, fallopian tube cancer	USA 2018
Chemotherapy	Abraxane	Paclitaxel	Albumin	iv.	Breast cancer, non-metastatic small cell lung cancer, metastatic adenocarcinoma of the pancreas	USA 2005
Chemotherapy	Ontak	Denileukin Diftitox	Protein	iv.	Cutaneous T-cell lymphoma	USA 1999
Chemotherapy	Genexol-PM	Paclitaxel	Polymeric micelles	iv.	Breast cancer, lung cancer	Korea 2007
Chemotherapy	Nanoxel	Paclitaxel	Polymeric micelles	iv.	Breast cancer, ovarian cancer	India 2007
Chemotherapy	Paclical	Paclitaxel	Polymeric micelles	iv.	Ovarian cancer	Russia 2015
Chemotherapy	PICN	Paclitaxel	Polymer/lipid NP	iv.	Metastatic breast cancer	India 2014
Chemotherapy	DHP107	Paclitaxel	Lipid NP	po.	Advanced gastric cancer	Korea 2016
Chemotherapy	LipoplatinTM, NanoplatinTM	Cisplatin	Liposomes	iv.	Pancreatic cancer, lung cancer	USA 2018
Hyperthermia	NanoTherm	Fe2O3	Metallic	iv.	Glioblastoma, prostate cancer, pancreatic cancer.	USA 2013
Hyperthermia	NanoTherm	—	Iron oxide NP	it.	Recurrent glioblastoma	Europe 2010
Radiotherapy enhancer	Hensify	—	Hafnium oxide NP	it.	Locally advanced soft tissue sarcoma	Europe 2019

*iv.* intravenous, *po*. peros, *it.* intratumoral

**Table 3:** Anti-tumor nanomedicines in clinical trials

Therapeutic type	Product name	Therapeutic agent	Nanocarrier	Administration route	Indications	Trial No.	Stage
Chemotherapy	ThermoDox	Doxorubicin	Liposome	iv.	Liver cancer	NCT02181075	Phase 1
Chemotherapy	LEP-ETU	Paclitaxel	Liposome	iv.	Neoplasm	NCT00080418	Phase 1
Chemotherapy	Nano-QUT	Quercetin	PLGA-PEG NPs	iv.	Oral cancer	NCT05456022	Phase 2
Chemotherapy	NK 105	Paclitaxel	Micellar nanoparticle	iv.	Breast cancer	NCT01644890	Phase 3
Chemotherapy	Docetaxel-PM	Docetaxel	polymeric micelle	iv.	Esophagus squamous cell carcinoma, metastatic cancer	NCT03585673	Phase 2
Chemotherapy	BIND-014	Docetaxel	Polymeric micelles	iv.	KRAS positive non-small cell lung cancer, squamous cell non-small cell lung cancer	NCT02283320	Phase 2
Chemotherapy	Genexol PM	Paclitaxel	Polymeric micelles	iv.	Bladder cancer, ureter cancer	NCT01426126	Phase 2
Chemotherapy	Nab-paclitaxel (Abraxane)	Paclitaxel	Albumin	iv.	Non-small cell lung cancer	NCT02016209	Phase 2
Chemotherapy	Abraxane	Paclitaxel	Albumin	iv.	Pancreatic cancer	NCT00691054	Phase 2
Chemotherapy	NC-6004	Cetuximab	polymeric micelle	iv.	Head and neck neoplasms	NCT02817113	Phase 1
Chemotherapy	MBP-426	Oxaliplatin	Liposome	iv.	cancer	NCT00355888	Phase 1
Chemotherapy	FF-10850	Topotecan	Liposomes	iv.	Advanced solid tumors	NCT04047251	Phase 1
Chemotherapy	Cetuximab nanoparticles	Cetuximab	Ethylcellulose nanoparticles	iv.	Colon cancer	NCT03774680	Phase 1
Chemotherapy	Docetaxel-PNP	Docetaxel	Liposome	iv.	Advanced solid malignancies	NCT01103791	Phase 1
Chemotherapy	ABT-888	Doxorubicin	PEGylated liposomes	iv.	Ovarian cancer	NCT01113957	Phase 2
Chemotherapy	CPX-351	Cytarabine and daunorubicin at 5:1 ratio	Liposomes	iv.	Acute myeloid leukemia	NCT02286726	Phase 2
Chemotherapy	LipoVNB	Vinorelbine tartrate	Liposomes	iv.	Advanced malignancy	NCT02925000	Phase 1/2
Chemotherapy	CriPec	Docetaxel	Polymeric micelles	iv.	Ovarian cancer	NCT03742713	Phase 2
Chemotherapy	LY01610	Irinotecan hydrochloride	Liposomes	iv.	Small cell lung cancer	NCT04381910	Phase 2
Chemotherapy	Liposome doxorubicin	Doxorubicin	Liposomes	iv.	Desmoid tumor	NCT05561036	Phase 3
Chemotherapy	EndoTAG-1	Paclitaxel	Cationic liposomes	iv.	Breast cancer	NCT01537536	Phase 2
Chemotherapy	KM1	Carboplatin	Virus	iv.	Ovarian cancer	NCT05684731	Phase 1
Immunotherapy	mRNA-NP vaccine	Autologous total tumor mRNA	Liposome	iv.	Melanoma	NCT05264974	Phase 1
Immunotherapy	Dex2	Antigen	Exosome	iv.	Non-small cell lung cancer	NCT01159288	Phase 2
Radiotherapy	AGuIX-NP	Cisplatin	Inorganic NPs	—	Gynecologic cancer	NCT03308604	Phase 1
Radiotherapy	AGuIX-NP	Temozolomide	Inorganic NPs	—	Glioblastoma	NCT04881032	Phase 1/2
Radiotherapy	NBTXR3	—	Hafnium Oxide NPs	it.	Advanced and/or metastatic malignant solid neoplasm, metastatic malignant liver cancer, metastatic malignant lung cancer	NCT05039632	Phase 1/2
Gene therapy	iExosomes	KRAS G12D siRNA	Exosomes	iv.	Metastatic pancreas cancer	NCT03608631	Phase 1

*iv.* intravenous, *po.* peros, *it.* intratumoral

## Materials to construct the drug carriers

Material design is the footstone for nanotechnology, with the hope of constructing drug delivery systems for tumor therapy. We have generally categorized the currently developed materials into the following types: polymeric NPs (including natural and synthetic ones), liposomes, micelles, hydrogels, exosomes, and other extracellular vesicles; natural membrane-coated NPs (including red blood cell-NPs, leukocyte-like carriers, and plateletsomes); and viruses; and inorganic NPs (including mesoporous silica, gold NPs, and carbon nanomaterials). Though nanomaterials are categorized into different types in this review, the combination strategy of various materials is not excluded. Notably, an increasing number of studies focus on hybrid materials, which can combine the advantages of two or more types of materials. For instance, polymeric materials can be modified on the surface of gold NPs to overcome the low rigidity of polymers and improve the biocompatibility of gold-based NPs.

### Polymeric nanoparticles

Polymeric nanoparticles include a wide range of biomaterials with sizes ranging from 10 to 100 nm. There are different classifications of polymeric nanoparticles.^38,76–78^ Generally, polymeric nanoparticles can be sorted into three subgroups: synthetic polymers, natural products, and hybrid ones, which combine the synthetic and the natural together with the potential to achieve various functions. All of them have featured characterizations in the application of nanoparticle drug delivery systems, including biodegradability, biocompatibility and nontoxicity. Herein, synthetic and natural polymeric nanoparticles and their properties are discussed.

#### Natural polymers

Natural polymeric nanocarriers are polymers firstly discovered and produced by organisms, including polysaccharides and protein NPs.^79^ Both can be easily acquired from natural organisms and put into use after fabrication. Strictly speaking, there is nearly no complete “natural polymer” that can be used as a nanocarrier without any modification.

The most widely used polysaccharide-based biomaterial in the architecture of nanomedicine is regarded as chitosan. Chitosan is a water-soluble, biocompatible, and biodegradable polysaccharide material that originates from the shells of crustaceans.^38,80,81^ Due to the excellent capability of transmucosa, chitosan-based biomaterials are commonly used for drug delivery systems through varied kinds of epithelia, such as the eye^82,83^ and the intestinal.^84,85^ Yeh et al.^86^ found chitosan can “permeabilize” the mucosa epithelium by means of mediating the opening of a tight junction between cells of the mucosa so that a drug or other therapeutic component can cross the mucosa barrier and act on malignant tissue. Therefore, the oral route of administration is widely recognized as the most comfortable and compliant approach for patients.^87,88^ Another advantage of chitosan is that it can inspire other scientists to modify or design brand new biomaterials with chitosan.^84,87–112^ In recent research,^87^ a new design of chitosan named AuNP-siRNA-glycol chitosan-taurocholic acid nanoparticles (AR-GT NPs) was used to treat liver metastasis from colorectal cancer in an animal trial. This delivery system has potential for treating liver-related tumors. In addition, chitosan itself has the potential to be an add-on therapy to upregulate the function of the immune system during the procedure of tumor treatment. According to Carroll et al., chitosan can induce type I interferons (IFN) to promote the maturation of dendritic cells and enhance the sensitivity of Th1 through the cGAS and STING pathways.^89^ Generally, the application of chitosan is very promising. However, chitosan and other nanocarriers with positive charges on their surfaces were once thought to be limited in clinical application due to their toxicity and inflammatory response.^113^ In the study of Wei et al.^113^, injection of chitosan could lead to rapid progress of necrocytosis because of intracellular Na^+^ overload caused by excessive “permeabilized effect” instead of inflammation. This is also a significant mechanism that has implications for the better design of safer chitosan-based nanocarriers. Taken together, chitosan has been widely adapted in the therapeutic approach with great hope, especially via oral administration; however, more basic or clinical trials and a more sophisticated design are required to ensure its unpredictable safety issues.

Another widely explored natural polymer-based nanocarrier is protein NPs, especially human serum albumin (HSA). HSA is potentially ideal for chemotherapeutic drug delivery due to several key characteristics, including: (1) it is an endogenous carrier from the human body, so it is unlikely to cause an inflammatory and corresponding toxic response; (2) it is a natural carrier for various ions and compounds in the circulatory system; (3) it has a long circulation time, so it can be remodeled to release drugs sequentially^114^; (4) it can be biodegraded and reproduced by metabolism^115^; (5) it tends to accumulate at the site of abnormal vasculature, including leakage or capillaries in the TME; (6) cancer cells, which require numerous types of substances to grow, are likely to approach HAS.^38,77,116^ Besides, like chitosan, HSA can also be modified to optimize its properties. As a typical protein, HSA has both carboxylic acid and amino groups, which enable HSA to have characteristic isoelectric points (pI). The pI of HSA is variable because the ratio of amino acids and acids can be mediated by chemical modification.^38^ Tian et al.^117^ have achieved flexible isoelectric points through this method in the gene transfer of virus vectors. HSA is also promising to deliver negatively charged molecules, including nucleic acid. As a drug, more trials are required to resolve the restriction on TME. A newly designed nanomaterial consisting of three components, including Au(1)Ag(9) (<8 nm; stimulus component), docetaxel (an anticancer drug), and bovine serum albumin, showed high efficacy and biosafety in chemotherapy therapy induced by near-infrared light.^118^ At present, however, the most tested application of HSA is HSA-based photothermal therapy (PTT) due to the precise localization characteristic of the tumor (most of them used bovine serum albumin, BSA, instead of HSA).^118–122^ Li et al. ^119^ combined BSA-based gadolinium oxide nanodots (GdNDs) with a small-molecule NIR-II fluorophore (FS) named FS-GdNDs, which demonstrated an excellent photothermal conversion efficiency of 43.99%, good photothermal stability, and favorable capability of MR/NIR-II imaging performance at low doses. During the toxicity test, it also showed outstanding biocompatibility and biodegradability, which are of enormous potential in photothermal applications. In addition, serum albumin is also explored in the areas of immunotherapy^123–125^ and radiotherapy,^126–130^ but they still require advanced study for clinical translation.

#### Synthetic polymers

Synthetic polymers for drug delivery have already been widely adopted in tumor treatment, with a relatively longer history than other nanoparticles. Some of them have been approved by the FDA for clinical applications, such as polylactic acid (PLA)^131–133^ and poly (lactic-co-glycolic acid) (PLGA).^134–136^ Recently, however, ever-decreasing concerns concentrate on the application of synthetic polymers solely but hybrid polymers such as polymeric micelles, which are mostly composed of both natural and synthetic polymers. When polymers are assembled into micelles, their functions and characteristics will be only partly reserved, and their applications will be different from each other.^137^

PLA is a biocompatible, biodegradable polymer, and its monomer, lactic acid, is a safe substance that is the basic particle in carbohydrate metabolism.^131^ PLGA, based on the structure of PLA, is the most widely used FDA-approved type of polymer. PLGA became well-known in 1994 when PLGA-PEG NPs were invented. PLGA inherits all of the advantages of PLA and has a long circulating half-life and continuous release properties, which makes it more suitable than other synthetic polymers.^39^ In recent studies, PLGA is not only ideal for chemotherapy drug delivery but also promising in immunotherapy^138,139^ and photothermal therapy,^138,140,141^ just like HSA. R837-αOVA-ApoE3-HNP,^142^ a recently designed nanovaccine, is composed of a PLGA core and a capsule of adjuvant imiquimod (R837), and a phospholipid membrane with antigen peptide (αOVA) and apolipoprotein E3 (ApoE3). Actually, this nanovaccine is a kind of antigenic analog that can boost antigen into DCs directly instead of being engulfed by DCs passively. After that, strong responses, including the maturation of specific CD8 (+) T cells, an increasing number of IFN-γ (+) CD8 (+) T cells, and the secretion of IFN-γ (+), were observed in this study, which showed tremendous potential to be a new method of cancer immunotherapy. By contrast, a recent study showed PLGA-carrying rapamycin can partly inhibit the activation of B cells and enhance the function of Treg cells, which can inhibit the development of antidrug antibodies in order to improve efficacy and minimize the hypersensitive effect during the agent treatment.^143^ It seems that PLGA tends to interact with not only tumor cells but also certain kinds of immune cells, which has significant guidance for scientists to tackle the TME dilemma.

Dendrimers, unlike other synthetic polymers, have a three-dimensional, repetitively branched shape, just as their name suggests (‘dendri’ means ‘tree-like’ in Greek). Due to this featured structure, dendrimer has a remarkable hydrophilicity with a small size of less than 20 nm,^144,145^ which makes it easy to cross biological barriers, including the mucosa, endothelium, and epithelium. Generally, dendrimers have several features, including: (1) nontoxicity; (2) bioavailability; (3) drug stability; (4) enhanced biological activities of the encapsulated and conjugated drugs; and (5) outstanding transmembrane capability. In addition, the most prominent feature of dendrimer is its highly multidirectional homogeneity.^146–149^ In this case, dendrimer can effectively interact with certain targets, including ligands or antigens, just like a polyvalent antibody. On one hand, dendrimer itself can be bioactive as a potential agent.^144,150^ On the other hand, dendrimers can be a platform to cooperate with other immunoactive substances, such as programmed death ligand 1 (PD-L1), a famous ICI agent. In a recent study,^151^ dendrimer-ICI conjugates (G7-aPD-L1) demonstrated better strength than free aPD-L1. From what has been discussed above, both effects provide possibilities for designing a new platform for cancer immunotherapy.

### Liposomes

Liposomes are spheres consisting of two lipid layers and encircling an aqueous core, ranging from 50 to 450 nm in size in medical applications.^152^ They are capable of carrying both hydrophilic (inside the aqueous solution core) and hydrophobic (associated within the lipid bilayer) molecules.^152^ Liposomes were firstly discovered by Alec Bangham in 1961^153^ and were among the first generation of lipid-based drug delivery systems introduced to clinics.^154^ Some liposome-formulated anti-cancer drugs have already been marketed, including Myocet liposomal, liposomal doxorubicin (DOX), and Marqibo, with several other liposomal formulations in clinical trials.^155–157^ There are generally four categories of liposomes: (a) traditional types, consisting of a lipid, mostly phospholipids; (b) bilayer PEGylated types, liposomes with polyethylene glycol (PEG) coating on the surface for a longer circulation period; (c) ligand-target types, linked with targeting ligands such as peptides, monoclonal antibodies, vitamins, or carbohydrates to target the desired cell types; (d) theranostic types, a multifunctional type with targeting ligands, imaging agents, and drugs inside.^158^

Liposome-based delivery systems have several advantages in terms of targeted and precise drug delivery, reducing systemic drug toxicity, ensuring a stable environment for drugs inside during transportation, and avoiding bio-clearance upon delivering gene therapy agents to the cytosol.^159–165^ Recent research shows liposomes conjugated to antibodies for EphA2, enclosing the docetaxel prodrug, can effectively reduce the toxicity of antitumor drugs, improve overall tolerability, maintain a desirable exposure of the drug in cancer tissue, and remarkably improve the antitumor activity in comparison with non-nanodelivery and non-targeted nanodelivery controls.^166^ There are also problems for liposome-based delivery, such as low storage of lipophilic molecules, opsonization, immunogenicity, and instability.^38,76,167^ The physical stimuli-responsive liposome has been a hotspot in recent years.^168–170^ Compared with conventional liposome-based medicines, they offer new practical options for controlled drug release at the desired sites, yet very few preclinical candidates have entered the clinical trial, not to mention reaching the hospital.^155,171,172^ These intelligent carriers face even more challenges than conventional ones, such as the choice of light source and wavelength settings, phospholipids with proper stimuli properties, and the toxicity of synthesized lipids.

### Micelles

Micelles are spheres composed of self-aggregated amphiphilic molecules with a size between 10 and 100 nm in diameter. In aqueous solutions, the monolayer of surfactants strikes a balance by thermodynamics, forming a hydrophilic surface contacting the medium as well as separating a hydrophobic core in the center.^173,174^ Both synthesized polymers and natural molecules can be utilized for the formation of micelles.^175^ However, it follows certain conditions, including the concentration of the minimal amphiphilic molecules, known as the critical micelle concentration (CMC), the structure of the chemical group, the temperature known as the Krafft temperature, under which micelles may collapse, and the medium.^176,177^ A wide variety of materials can be encapsulated into the micellar core; as a result, toxicity caused by the widespread administration of drugs can be alleviated.^178,179^ Meanwhile, it enables easier transportation of lipophilic drugs, which are otherwise hard to dissolve, and quickly eliminated them from the internal environment.^180^

Block polymers, with the fundamental components of a shell-forming part and a core-forming part, have different properties based on the materials. Several materials are available for micellar shells, including poly(ethylene glycol), polyvinyl alcohol, poly(*N*-vinyl-2-pyrrolidone), poly[*N*-(2-hydroxypropyl) methacrylamide], poly(oxazolines), poly(amino acid)s, zwitterionic polymers, and polysaccharides. Some are for cores, including polyether, polyester, polyamino acids, poly(ethylene imine), and poly(amino acid)s, all with different inherent properties.^137,181,182^ Poly(ethylene glycol), or PEG, one of the most investigated materials for the shell, creates a sheath on the surface of the micelles, thus reducing the uptake by macrophages and prolonging the circulation time.^183–187^ Regardless of the type of micelles, the selection of polymers should always consider these vital aspects: the type of loading drugs, biocompatibility, immunogenicity, toxicity, biodegradability, and special other properties.^175,188–190^ As reported, fine-adjustment of the components of micelles makes a precise spatiotemporal control available by enabling the micelles to respond to endogenous (for instance, pH, enzymes, ROS, and ATP) or exogenous stimuli (including light, temperature, and ultrasound treatment).^137,191^ Those properties can be combined when needed, functioning as double insurance for targeted drug releases. In recent research,^192^ Su et al. designed a pH and metalloproteinase double-sensitive PEGylated micelle for transporting and releasing anti-programmed death 1 (PD-1) antibody and paclitaxel in a targeted manner and achieved on-demand sequential release of antitumor medicine. Apart from reacting to stimuli, targeted micelles can be achieved by enhancing penetrability and retention through attaching ligands or monoclonal antibodies to the surface.^178,193^ As several micelle-based medicines enter the market, micelles seem to enjoy an optimistic future in clinical applications.^38^

### Hydrogels

Hydrogels are 3-dimensional chemically or physically crosslinked polymer chain networks with versatile properties achieved by fine-tuning.^194–196^ Cross-linking physical interactions mainly include hydrogen bonds, while chemical ones are mainly covalent bonds. Hydrogels are highly biocompatible, capable of loading several types of drugs simultaneously, controllable and adjustable in drug release, sensitive to stimuli, and capable of transferring physical states, which makes them potentially valuable for resolving obstacles in cancer management that the current drug delivery systems may encounter.^194,197–202^ Due to their highly adjustable chemical and physical properties, hydrogels are widely utilized in a great number of medical fields, including tissue engineering, wound healing, medical imaging, environmentally sensitive drug delivery, cancer vaccines, gene editing, biosensors, cell culture, and so on.^203–212^

The nanotherapeutic systems have faced obstacles in clinical trials due to inadequate dispersion within the body, limited ability to break down naturally, harmful effects on cells, and inconsistent effectiveness.^213^ Hydrogels are a newly developed polymer substance that closely resembles healthy tissues, making them very compatible with biological systems. Biological organisms exhibit a high degree of tolerance towards them, as they have minimal levels of toxicity. In addition, nanohydrogels possess a comparatively low surface to volume ratio when compared to numerous other nanomaterials, which aids in the process of cellular uptake.^214^ Nanohydrogels can be generated from either natural polymers or synthetic polymers. Natural polymers possess benefits such as exceptional biodegradability, biocompatibility, and the ability to be easily excreted or cleared by the kidneys. On the other hand, synthetic polymers offer stability, superior mechanical qualities, the capacity to regulate their structure, and favorable characteristics for drug release.^215^ The characteristics of nanohydrogels render them a highly promising carrier for delivering anticancer drugs. Furthermore, their controlled release, enhanced permeability, and protection of loaded drugs allow nanohydrogels to serve as topical drug delivery systems, potentially improving drug delivery through the skin and treating skin diseases, including cutaneous malignancies.^216^

Immune checkpoint blockers (ICBs) are a promising targeting strategy in cancer immunotherapy, yet they only benefit a small proportion of patients. Numerous studies have been investigated to tackle this problem, and recent research with a hydrogel endeavor seems to yield optimistic results. Wang et al. designed a prodrug hydrogelator to locally transport ICBs, boosting immunity against brain and colon cancer.^217^ Hydrogel functioned as a reservoir for long-term and stimulated discharge of camptothecin and aPD1 at the tumor site, resulting in a 100% tumor regression on all mice models. Meanwhile, hydrogels can be designed for enhanced penetration and retention, which is also a hotspot in cancer immunology research. Recent research showed the supramolecular tubustecan hydrogel formed hydrogel spheres upon being injected into the tumor tissues.^218^ They were capable of encapsulating the antitumor drug DOX, or curcumin, and enhancing penetration and retention. Besides that, hydrogels loaded with other nanoparticles provide us with a broader platform for combining multiple types of nanomedicine to solve the problem of tumor multidrug resistance.^219^

### Exosomes and other extracellular vesicles

Extracellular vesicles (EVs) are particles with a cell-originated membrane released by cells. Loaded with biological components with unrecognized functions, EVs were initially believed to be more like carriers of metabolites, waste, or cell debris, and their functions were gradually identified over time.^220,221^ Exosomes are tiny, single-membrane-enclosed vesicle organelles released by cells, with a diameter ranging from 30 to 200 nm and containing proteins, lipids, and other biological components.^222,223^ It is one of the most important and well-recognized EVs. Apart from exosomes, the first type of identified extracellular vesicles,^224^ there is an increasing number of EVs that are being recognized, and their classification is constantly and rapidly evolving.^225^

EVs, especially exosomes, are biologically important for a variety of cellular behaviors and intercellular communication. Recent research has revealed their significant importance in neoplasia, tumor growth promotion, local immunosuppression, and metastasis.^226–234^ However, EVs have the potential to be developed and utilized in several fields regarding cancer diagnosis and management. To our best knowledge, research on EVs is focused on three scopes: (i) non-invasive cancer diagnosis,^235–237^ achieved by various technologies of detecting exosomes and recognizing their biochemical components, which gives a hint of the biological components and pathophysiological conditions of the donor cells^222,238^; (ii) targeted drug vehicles, as the biocomponent of the membrane reflects that of the donor cell, making it possible to combine preferentially with certain cells^235,239,240^; (iii) EVs-based cancer immunotherapy, contributing to an activation of immunity towards tumor cells.

EVs are especially appropriate vehicles for targeted drug delivery because they are natural vesicles deriving from cells that are highly biocompatible. EVs from different kinds of cells are suitable for loading a large variety of cargos with precise modification.^241–243^ Oligonucleotide, natural or synthesized compounds are mostly studied and most frequently used drugs carried by EVs, among which RNA carried by EVs has been regarded as a great breakthrough in cancer nanodrugs.^225,244,245^ Mesenchymal stem cell-derived exosomes were reported to be used as carriers of microRNA-124a to treat glioblastoma.^246^ In vitro experiments showed that anti-glioma microRNAs containing exosomes remarkably reduced the viability and clonogenicity of cancer cells compared with the control groups. In vivo experiments showed favorable survival benefits, and 50% of mice had at least 110 days of life. Sancho-Albero et al. designed a cancer-derived exosome for transporting biorthogonal catalytic drugs into cancer cells.^247^ The catalysts were transported by those “Trojan Horses” exosomes and activated the prodrugs within them. Besides the normal cargos mentioned above, several new combinations of nanomedicines are constantly emerging, making it possible to combine cancer-treating strategies like gene therapy and attempts with oncolytic viruses.^248,249^

Cancer immunotherapy, which generates or enhances immunity against cancer cells, has attracted increasing attention in the past decade.^250^ EVs play significant roles in modulating the immune responses to cancer, both in positive and negative ways.^251–253^ Among all kinds of EVs, tumor-derived EVs (TEVs) and immune cell-derived EVs are regarded as promising new directions in tumor immunotherapy. One major characteristic of TEVs is that several kinds of immunogenic molecules are on the surface, which can induce the immune response against cancer cells, including Hsp 70, miRNAs, and MHC-1^225,254–258^ and could be developed as cancer vaccines.^259^

Functions of immune cell-derived EVs include presenting antigen, activating immune cells, Treg cell differentiation, suppressing immune responses, and suppressing inflammation.^252,260^ For example, EVs derived from NK cells (NK-EVs) have been reported to share similar cytotoxic abilities and have been used as anti-tumor therapies.^261–263^ Zhu et al. recently reported a method to enhance this potency.^261^ NK-EVs were priming with interleukin (IL)-15 and were separated from the normal NK-EVs. They were injected separately into cancer model mice intravenously, and NK-EVs (IL-15) showed significantly stronger cytolytic properties towards human tumor tissues. At the same time, gene expressions related to the cytotoxicity of NK cells were also promoted. Dendritic cell-derived exosomes (DEXs) were also utilized to treat cancer. Lu et al. reported using exosomes released from α-fetoprotein-expressing DCs (DEX_AFP_) in hepatocellular carcinoma (HCC) mice models.^264^ Mice treated with DEX_AFP_ had significantly improved survival rates and better control of tumor growth.

Although some crucial problems remain to be solved, such as interference of biochemical backgrounds, technologies for better isolation and purification, differential recognition of exosomal surface compounds, lack of understanding of the biology of EVs, abnormal accumulation of EVs in the liver, etc.,^236,238,265–267^ the emerging research aimed at solving these problems gave us great expectations of a potential giant leap in the diagnostic and therapeutic applications of EVs.

### Natural membrane-coated NPs

Besides natural extracellular vesicles, artificial nanoparticles with natural membranes are being investigated during these years in the field of drug delivery. Like natural EVs, these cell-mimicking nanoparticles, with characteristics borrowed from natural ones, can be highly biocompatible and exhibit limited immunogenicity.^268,269^ Membrane from several kinds of cells can be used to artificially synthesize nanoparticles with different properties according to the original cell.^270–272^

#### Red blood cell-NPs

Erythrocyte-originated nanoparticles, also called RBC-membrane-coated nanoparticles (RBC-NPs), are commonly investigated as drug delivery vehicles. RBC-NPs have shown several priorities compared with normal drug delivery systems: (i) prolonging the blood circulation time for the loaded drugs^241,273–278^; (ii) evading clearance from the immune system^273–275,277^; (iii) boosting the therapeutic effects of loaded drugs^241,274,276,278,279^; and (iv) improving the cancer cell target capabilities when integrating biomimetic modifications.^275,280^ Interestingly, several researchers combined membranes from erythrocytes and cancer cells to create a hybrid one, with the intention of utilizing the advantages of RBC-NPs to homotypically target cancer cells.^274,276^ Jiang et al. fused the erythrocyte membrane with the MCF-7 (breast cancer cell line) membrane and produced hybrid melanin particles for in vivo photothermal treatment.^274^ Bearing proteins from both RBC and cancer cells onto the surface, together with a melanin core, the fused nanoparticles showed enhanced antitumor properties, along with several advantages listed above. In their research, the proportion of the fusion membrane was also discussed, which may be a crucial problem in similar coatings of NPs with merged membranes in the coming study. Looking ahead, NPs coated with cell membranes could be an interesting way of adding or augmenting the desired characteristics of nanomedicines. However, it still should be recognized that the surface property, intracellular interactions, and circulating capability of the cell membrane-coated nanomedicine are not completely equal to those of the original cells, since during the membrane preparation, fusion, and coating procedures, some compositions of the membrane might be finely tuned.

#### Leukocyte-like carriers

Leukocyte-like carriers, fabricated either by coating nanoparticles with leukocyte membranes^272,281,282^ or by decorating liposomal nanoparticles with biochemical components from leukocyte membranes,^283^ are leukocyte-mimicking nanoparticles designed for improving pharmaceutical efficacies and reducing toxicities. Most research on leukocyte-like carriers has focused on their ability to target tissues with inflammation,^282,284–286^ which is a basic pathological process in many diseases, including cancer.^287^ Palomba et al. investigated the functions of the leukocyte membrane on silicon particles.^281^ With several critical receptors on the surface, those particles exhibited higher abilities of permeating tumor blood vessels. Leukocyte-like carriers have other possibilities when combined with tumor cell membranes. They et al. designed composite nanoparticles composed of exogenous phospholipids fused with the leukocyte and tumor cell membranes, namely leutusomes, with a paclitaxel (PTX)-constructed core.^283^ Compared with liposomes consisting of only leukocytes or cancer cell membranes, leutusomes showed advanced tumor-retention ability with few systemic adverse effects. The research group noticed this phenomenon in leutusomes with membrane components from both head and neck tumor cells (HN12 and B16 melanoma cells), suggesting the potential generalization of this tactic.

#### Plateletsomes

Platelets, one of the most important modulators of homeostasis, have long been known to play promoting roles in tumor progression and metastasis.^288,289^ Thus, there has been an anti-tumor strategy to deplete tumor-associated platelets or suppress their activities in a tumor-related environment.^290,291^ Besides, there is growing interest in NPs with platelet membrane or platelet-membrane moieties as new treatments for cancer, among which platelet membrane-coated NPs are receiving the most attention. Recently, research reported small interfering RNA (siRNA) delivered by a platelet membrane-enclosed metal-organic framework with high loading capacity and pH sensitivity.^292^ The membrane enabled particles with specific cancer-binding abilities while silencing target genes efficiently in vitro and achieving fine therapeutic efficacy in vivo. Also, another combination of leukocyte membrane and nano-magnetic nanoparticles was reported, which sensitized effective ferroptosis and boosted the immune response triggered by anticancer agents.^293^

### Virus

In contrast to the nanoparticles explained above, virus NPs, or virus-like nanoparticles (VLPs), are relatively unexplored. Viral vectors, such as adenoviruses, have shown success in gene therapy as well as in treating tumors. VLPs were once considered an optional deliver that could carry antibodies, small molecule-based drugs, siRNA, and contrast agents^268,294^ because of their outstanding targeting capability. In the most up-to-date study, since VLPs consist of the nontoxic viral capsid with antigen-analog, antigen-presenting cells, including DC,^295^ will take in the VLPs so that targeted transportation is achieved. After the process of immune response, certain immune cells will be upregulated and attack tumor cells, which means VLPs can mediate the function of the immune system. Although limited studies showed the advantages of VLPs in tumor treatment, several studies exhibit their potential in immunotherapy or vaccination strategies. Generally, VLPs can be sorted into plant VLPs and other VLPs. Due to their non-pathogenicity with humans, researchers have achieved more progress in plant VLPs than others. Potato virus X (PVX) has been reported to exhibit excellent performance as an immunotherapy.^296^ Lee et al.^296^ designed double-functional NPs (PVX-DOX) by combining DOX with PVX in order to produce both immunotherapeutic and chemotherapeutic effects on B16F10 melanoma. In this study, PVX played a critical role not only in drug delivery but also as an in-situ vaccine. More importantly, PVX+DOX was reported to have better efficacy for in situ vaccination than DOX carried by PVX, which means their interactions enhanced their effects when they were used separately. Another promising plant virus is cowpea mosaic virus (CPMV), which can be applied in tumor imagination and chemotherapeutic delivery due to its specific icosahedral shape.^297^ Nonetheless, the purity of carrier-used CPMV is not enough that some foreign matters, such as lipopolysaccharides, can lead to an unexpected hypersensitivity response.^298^ A new Lyo-eCPMV was introduced by Zheng et al. to tackle this dilemma.^298^ In addition, other VLPs, including Hepatitis B virus core (HBc) particles,^299^ HPV16 L1 VLP,^300^ and bacteriophage Qβ VLP,^295,301^ have potential clinical values, especially in immunotherapy. However, more studies are required for clinical translation.

### Inorganic NPs

Inorganic NPs, as drug carriers, have distinct physical, chemical, and biological properties due to their small size, large surface area to volume ratio, high drug loading capacity, controllable release, great biocompatibility, and biological stability. These characteristics make them extremely promising for use in a variety of medication delivery applications.

Despite the exceptional performance of inorganic nanoparticles, it is crucial to address the safety risks associated with them. After ingestion, biological distribution, and metabolism, inorganic nanoparticles can be excreted from the body through the kidneys via urine or the liver via bile. Nonetheless, some studies have indicated that certain types of inorganic nanoparticles might not degrade or be excreted.^302^ Furthermore, under certain circumstances, inadequate elimination may cause certain nanoparticles to remain in the body for a long time. Consequently, they may disrupt the normal function of organs or tissues and induce toxicity.^303^ This underscores the importance of not overlooking safety assessment while focusing on efficacy.

#### Mesoporous silica

Mesoporous silica, which can be produced utilizing structure-directed agents (SDAs) co-packaged with a silica precursor in the presence of a catalyst, is made of an amorphous silicon dioxide wall with equally dispersed pores (mesoporous structure).^304–306^ There are many advantages making it possible to utilize this kind of NP to develop controlled release systems, such as easy chemical modification on surface and interior pores, high capacity for storing anticancer drugs, and a simple production process.^306,307^ Mesoporous silica nanoparticles (MSNs) are internalized into cells by endocytosis, phagocytosis, and pinocytosis, depending on size, aspect ratio, and surface molecules.^308^ After entering cells, MSNs can release the encapsulated drug only by particularly interacting with intrinsic or extrinsic stimuli, and the release of “gatekeeper” is followed.^309–311^ Most MSNs will encounter an acidic environment in the endosome or lysosome after being absorbed, so a low pH is regarded as a trigger for content release from pores. Based on this conception, Liu et al. designed a kind of MSN called MSN/DOX@CaCO_3_@CM,^312^ in which the core was filled with a highly efficient DOX and CaCO_3_ interlayer. Their purpose was to regulate drug release in accordance with pH levels; furthermore, the encapsulation of the cancer cell membrane around the outer layer could enhance the stability of the colloid and promote tumor accumulation. Another important inner stimulus is the redox condition. Liang et al. invented a redox-sensitive DOX-loaded MSN called MSN-SS-GHA (DOX@MSN-SS-GHA),^313^ which shows promise for efficient redox-responsive targeting drug delivery and magnetic resonance imaging (MRI). Besides, magnetic fields and light are important external factors to promote drug release. A new MSN, referred to as MMSNs cloaked with RBC membrane, has been created to effectively combine photosensitizer delivery, immunological adjuvant, and MF-assisted targeting photodynamic therapy, offering a novel strategy for cancer treatment.^314^ As for the excretion, studies have shown that a large variety of silicon materials were detected in the feces and urine of animal models after injection and were likely to metabolize in the liver, be removed in the intestine, or be cleared by the kidney due to their highly positive charge.^315,316^ In addition, MSNs can be degraded into silicic acid,^317^ which occurs during drug delivery and ultrasound or MRI,^318,319^ which might be applied to follow the trail of transport and distribution of the particles in circulation.^320^ Because of the structural stability of MSNs, siRNA and low-molecular-weight polyethylene imine (PEI) can be bound to particles together through the interaction of anion and cation. With this method, siRNA will not be hydrolyzed by endonuclease in the serum or cytoplasm, so as to shut down the expression of the tumor gene.^321^ Recently, it was reported that MSNs carrying siRNA against TWIST plus cisplatin can overcome the clinical challenges of chemical drug resistance and tumor metastasis in epithelial ovarian cancer and other cancers with TWIST overexpression.^322^

#### Gold NPs

In addition to MSNs, gold nanoparticles have broad application prospects in cancer diagnosis and treatment because of their unique optical and Surface Plasmon Resonance (SPR) properties.^323,324^ The synthesis method of gold nanoparticles is relatively simple and consists of three main methods: physical, chemical, and biological processes. Biosynthesis is the most representative and attractive synthetic method in recent years, including plant^325–328^ and microbial-mediated^329^ synthesis. The shape of gold nanoparticles, such as gold nanocages, nanorods, nanocubes, nanostars, and nanospheres,^330^ is synthesized by different methods, which also determines that these different shapes of gold nanoparticles have different characteristics and different utilizations. For example, Yang et al. demonstrated that the shape-dependent SPR response of colloidal AuNPs and their respective effects can promote PDT efficiency.^331^ When gold NPs enter the body, they will be covered by various proteins in the physiological environment, such as albumin, collagen fiber, IgG, IgM, and transferrin, forming a “corona”,^332^ also known as the protein complex of nanoparticles, which is closely related to the transportation of nanoparticles in plasma, exchange with histones, and biodistribution.^333^ Meanwhile, the artificially designed NP protein complex coat can prevent the particles from being recognized by the immune system, thus prolonging their half-life in the blood.^334^ The chemical modification of gold nanoparticles is based on the negative potential on their surface. There are many kinds of molecules, such as small-molecule-based drugs, targeted ligands, genes, and so on. It has been reported that peptide-drug conjugates have demonstrated promising potential for increasing the targeted effectiveness of chemotherapeutic medications. Nevertheless, their limited uses may arise from their relatively short half-lives. However, this challenge can be solved by conveniently conjugating them with gold NPs.^335^ The characteristics of biocompatibility, non-toxicity, and no side effects contributed greatly to the anti-cancer therapy. What’s more, gold NPs can be used in photothermal therapy, photodynamic therapy, and photoimaging based on their favorable conductivity for heat.^336,337^ Photothermal therapy, wherein NPs encapsulated within tumors produce thermal energy when exposed to exogenously administered laser light, has been extensively documented as a unique approach to treating cancer with exceptional selectivity. Gold NPs play a crucial role in photothermal applications due to several advantageous properties they possess. Firstly, gold-based NPs exhibit biocompatibility, making them suitable for use in biological systems. Additionally, their small diameters facilitate tumor penetration when delivered systemically. Moreover, gold-thiol bioconjugation chemistry provides a straightforward method for attaching desired molecules. Furthermore, gold-based NPs demonstrate efficient conversion of light to heat, making them effective in photothermal processes. Lastly, their capacity to be adjusted to absorb near-infrared light allows them to penetrate tissues more deeply than other wavelengths.^338,339^ Through continuous integration, we found that the development of inorganic nanoparticles is not independent and separate.

#### Carbon nanomaterials

Carbon NPs have been shown to be a highly valuable subset of nanomaterials. Carbon-based nanomaterials, encompassing carbon dots, carbon nanotubes, and graphene, have been widely employed across diverse consumer and industrial sectors. These applications involve the improvement of biomedical vehicles, composites, electronics, sporting equipment, and lubricants.^318,340–343^ The relative proportions of sp, sp2, and sp3 hybridizations in carbon nanomaterials have a crucial role in determining the structural characteristics of these materials. Specifically, these hybridizations govern the development of two-dimensional nanomaterials with a flat morphology, such as graphene, one-dimensional nanomaterials with a hollow structure, like carbon nanotubes, as well as closed nanomaterials with zero-dimensional attributes, such as nanodiamonds. Furthermore, this ratio additionally influences several characteristics of carbon nanomaterials, including their structural integrity, electrical conductivity, chemical reactivity, and magnetic behavior, all of which contribute to the distinct benefits of different carbon nanomaterials in various applications.^344^ In this review, we mainly focus on two types of carbon materials: carbon nanotubes (CNTs) and nanodiamonds (NDs). The unique electrostatic fields of NDs are characterized by eight square facets with positive charges, three hexagonal facets with negative charges, and three hexagonal facets with neutral or intermediate charges.

Based on these chemical modifications on NDs, we can covalently attach anticancer drugs to their surface to solve the problem of drug resistance.^345,346^ For example, Liao et al.^347^ developed a nanocomposite consisting of ND conjugated with paclitaxel (PTX) and cetuximab (Cet) to specifically target EGFR-positive triple-negative breast cancer (TNBC) cells. Their study showed that the ND-PTX-Cet nanocomposite effectively induced mitotic catastrophe and apoptosis in TNBC cells by targeting the EGFR receptor. This finding suggested that the ND-PTX-Cet nanocomposite could be a promising therapeutic strategy for TNBC. Additionally, the flexibility to adjust functional groups on NDs and the natural fluorescence of fluorescent nanodiamonds (FNDs) make NDs a compelling platform for creating biomedical imaging tools and clinical diagnostics.^348^

Similar to NDs, CNTs can undergo various surface changes to enhance their functionality. The combination of this feature, together with the intrinsic benefits of CNTs, such as their high flexibility, tensile strength, and electrical conductivity, makes CNTs a desirable platform for various biological applications.^349–352^ Covalent functionalization of CNTs was employed to create a capping technique that responds to stimuli. In this technique, gold NPs were designed to obstruct the open ends of multiwalled CNTs (MWCNTs).^353^ The caps consisted of 40-nm gold nanoparticles that were attached to oxidized CNTs using linkers that were responsive to biochemical stimuli. These stimuli included changes in pH (achieved through a hydrazone bond that is broken under acidic conditions in tumors), a highly reducing environment (achieved through a disulfide bond that breaks in the presence of higher levels of glutathione inside cells), and esterase concentration (achieved through an ester bond). High-resolution imaging verified the existence of gold NPs mostly at the ends of CNTs, indicating the successful formation of a covalent bond between the gold nanoparticles and the open tips of the CNTs.^354^

Many particles contain two or more inorganic materials to form a hybrid. A variety of multi-material mixed inorganic nanoparticles exploit the advantages of single-material NPs and make up for the shortage of single-material nanoparticles. Meanwhile, the price is relatively low, which is more conducive to the realization of clinical translation and large-scale production.

Different designs and different types of materials are always exhibiting various functions and properties, based on which the downstream applications should be carefully considered after understanding the detailed pathological features and microenvironment of different tumors. In other words, tailorable materials-based nanotechnology is serving the tumor therapy requirements. During the research, there are two strategies that can be followed: (1) the biological characteristics of the tumor are well understood, which pushes us to find appropriate nanomedicines to overcome the dilemma that traditional protocols cannot solve; (2) novel materials with newfangled capabilities are first recognized or developed, urging scientists to search for their potential fit applications among the tumors. Both strategies require us not to be doctrinal and inflexible in the investigation of such stereotypes devoid of innovative contents.

## Nanomedicine in digestive system tumors

Digestive system tumors are common malignant tumors, including colorectal cancer, gastric cancer, esophageal cancer, liver cancer, pancreatic cancer, and other digestive organ cancers. According to the latest cancer statistics, the number of new cases and deaths of digestive system tumors is estimated to be the first of all tumors in the United States in 2023.^1^ Colorectal cancer ranks as the second leading cause of cancer-related fatalities,^355,356^ and most colorectal cancers originate from cancer stem cells (CSCs) in colorectal inner wall polyps, which are the accumulation of genetic and epigenetic variations.^357^ Liver cancer is the sixth in terms of global cancer incidence and the fourth in terms of cancer-related deaths. The primary risk factors comprise chronic hepatitis B virus (HBV), hepatitis C virus (HCV), excessive alcohol use, aflatoxin infection, smoking, type II diabetes, obesity, and others.^358,359^ Pancreatic cancer, sometimes referred to as the “king of cancers,” is recognized as one of the most challenging malignant tumors globally, presenting a 5-year survival rate of less than 9%. By 2030, pancreatic cancer is estimated to become the second most prevalent malignant tumor globally.^360^ Gastric cancer and esophageal cancer rank as the fifth and sixth most prevalent forms of cancer worldwide, respectively. The mortality rate of gastric cancer ranks second behind lung cancer, while over half of all cases of esophageal cancer are reported in China.^358,361^

Although traditional therapy (surgery, radiotherapy, and chemotherapy) has improved the treatment of digestive system tumors, it is accompanied by many side effects, including toxicity and drug resistance to normal cells and tissues. There is an urgent requirement to develop a novel treatment approach that can enhance the efficacy of treatment and minimize the occurrence of adverse effects.

In the past decades, nanotechnology has made considerable progress, and nanotechnology has also been widely used in the research of digestive system tumors, including NPs, dendrimers, liposomes, polymers, light-triggered therapy, and nanotechnology combined for diagnosis or treatment.^362–366^ Here, we summarize the research results of novel nanotechnology applied to digestive system tumors in recent years and provide new prospects for early screening and treatment for digestive system tumors.

### Diagnosis

Like other types of cancer, monitoring and diagnosis of digestive system malignancies mostly rely on the identification of tumor biomarkers and the utilization of imaging techniques. The early detection of tumors mainly depends on blood biomarkers, but most of the biomarkers fall off the tumor. After blood circulation, the secreted biomarkers are diluted, leading to a lack of specificity. Imaging tactics include CT, MRI, colonoscopy, endoscopic ultrasonography (EUS), and so on.^367–370^ However, they have similar problems, such as low sensitivity. Nanotechnology has obvious advantages in enhancing the specificity of biomarker detection, improving imaging effect, imaging time, and targeting, achieving local tumor aggregation, and reducing non-specific interference.

A nanosensor is a highly sensitive and specific tumor detection method. Loynachan et al. designed a multi-protease nanosensor for exogenous drug release.^371^ The sensor takes the renally cleared catalytic AuNCs as the template and couples them with a neutral avidin protein scaffold through a biotinylated protease-cleavable peptide connector to form the AuNC-NAV complex, which is stable in vivo without interference and retains catalytic activity. In the diseased site, it was disassembled due to the disorder of protease activity, including serine protease thrombin and zinc-dependent matrix metalloproteinase 9 (MMP9), among which the MMP-responsive AuNC-P2 20-NAV nanosensor was cleaved by MMP in the tumor site with abnormally higher MMP expression and released AuNC with a size of about 2 mm. AuNC was discharged into the urine through renal filtration, and then the disease status was detected simply and sensitively by detecting the ability of AuNC to catalyze peroxidase substrate. The results showed that the urine colorimetric signal of colon cancer mice was stronger than that of healthy mice, and the specificity of biomarkers was overcome by monitoring protease activity. At the same time, the nanosensor can be eliminated by renal excretion without being toxic. The up-regulation of significant biomarkers often occurs in hepatocellular carcinoma, and the individual differences are large. High-sensitivity detection of multiple biomarkers is very important for the early detection and diagnosis of liver cancer. Based on the enhanced Raman scattering (SERS) frequency shift immunoassay, Tang et al. designed a SERS-responsive silver nanoparticle film to simultaneously detect α-fetoprotein and glypican-3, which improved the sensitivity of liver cancer detection.^372^ CA19-9, a mucin-glycoprotein tumor marker, is the most sensitive marker for pancreatic cancer reported so far. The serum CA19-9 level in most patients with pancreatic cancer is significantly increased.^373^ Thapa et al. used carbon nanotubes (CNT) and PEI to construct thin films on the gold surface through the layer-by-layer (LBL) protocol. NHS-EDC was used to activate the carboxylic group on the CNT, and an anti-CA19-9 antibody was anchored on the surface of the membrane to form a biosensor. The detection limit of CA19-9 was 0.35 U/mL in buffer solution by impedance spectroscopy. Meanwhile, the samples containing glucose, ascorbic acid, and p53 antigen were tested to confirm the selectivity of the biosensor for the CA19-9 biomarker.^374^

The current CT and MRI imaging technology cannot accurately detect and visualize cancer staging. The combination of nanotechnology and endoscopic ultrasound (EUS), CT, and MRI to achieve active targeted imaging can increase the practicability of early screening and improve tumor monitoring. Liu et al. prepared NPs with diethylenetriamine pentaacetic acid (DTPA) by solvent diffusion method and then synthesized PLA-PEG-PLL-Gd NPs by chelating Gadolinium (Gd) ion with DTPA group on the surface of NPs. The PLA-PEG-PLL-Gd NP was further modified with a vascular endothelial growth factor (VEGF) antibody to obtain a new multifunctional polymer nano contrast agent (anti-VEGF PLA-PEG-PLL-Gd NPs) with an average size of 69.8 ± 5.3 nm. Compared with the non-VEGF-modified nanoparticles, the uptake of VEGF PLA-PEG-PLL-Gd NPs in cells increased. In vivo and in vitro MRI showed that the contrast agent could significantly improve the relaxation of the chelating unit and enhance the imaging signal. The duration of imaging was observed to be substantially extended from less than one hour to twelve hours, suggesting that the utilization of anti-VEGF PLA-PEG-PLL-Gd NPs as a nano contrast agent holds considerable promise for the early detection of liver cancers.^375^ Li et al. prepared a targeted uPAR nanoprobe DGLU11 by using endrograft poly-*L*-lysine (DGL) as a platform to couple the uPAR targeted peptide U11, gadolinium diethylenetriamine penta acetic acid (Gd DTPA), and cyanine dye cy5.5. The dual-mode MR/near-infrared fluorescence (NIRF)-targeted molecular imaging of precancerous pancreatic intraepithelial neoplasia (PanIN) and pancreatic ductal adenocarcinoma (PDAC) lesions was performed. The results revealed that the targeted probe had higher sensitivity in fluorescence imaging of MRI images.^376^ Shi et al. reported that Gd-dopping CuS NPs combined with tumor targeting and MMP-2 can be effectively used in magnetic resonance biomimetic/fluorescence imaging of gastric cancer. The study demonstrated that T-MAN nanoprobes can identify lymph node and gastric cancer metastasis in mice.^377^

One of the largest advantages of nanotechnology in cancer treatment is that it can overcome the dilemma of drug delivery and in vivo toxicity. Nanotechnology provides a platform for the delivery of insoluble or unstable drugs and improves the bioavailability and efficacy of drugs.

### Treatment

#### Chemotherapy

Traditional chemotherapy lacks specificity, and the concentration of drugs in tumors is so low that it often needs a high dose to give full play to the curative effect. The advent of nanotechnology has resulted in the extension of drug retention within the body, augmented drug accumulation at tumor locations through passive and active targeting mechanisms, and improved the specificity and efficacy of chemical therapy.^378–385^ Chen et al. designed superparamagnetic iron oxide with high magnetization and loss power (MnFe_2_O_4_@CoFe_2_O_4_) silica NPs loaded with DOX. The drug can be controlled and released by magnetic heating under an alternating magnetic field (AMF), which can effectively reduce the activity of pancreatic cancer cells.^386^ According to the EPR effect of tumor tissue, nanodrug can effectively penetrate and stay in the tumor lesion. Cervello et al. synthesized a brush copolymer PHEA-BIB-ButMA (PBB) using α-poly (*N*-2-hydroxyethyl)-*D*, *L*-aspartic acid (PHEA), and poly (butyl methacrylate) (ButMA) as raw materials by the atom transfer radical polymerization (ATRP) method.^387^ Then, the NPs loaded with sorafenib were prepared by the dialysis method for in vitro and in vivo anti-liver cancer research. The results showed that the nano-drug could accumulate significantly at the tumor site, and the anticancer effect of the drugs was enhanced. Based on cell penetrating peptide (CPP) and PDAC homing in vivo, He et al. modified the aptamer (GBI-10) targeting extracellular matrix (ECM) component (tenescin-C) onto matrix permeable CPP to prepare aptamer/cell penetrating peptide-camptothecin prodrug Apt/CPP-CPTD NPs.^388^ As a tumor-homing ligand, aptamer can target tumors and realize the accumulation of nanodrugs in the tumor site. At the same time, tenascin-C present in the PDAC matrix has the ability to segregate GBI-10 from CPP, and the presence of exposed CPP can facilitate PDAC’s continued penetration and tumor cell endocytosis. Following the internalization of Apt/CP-CPTD NP into PDAC cells, a heightened intracellular redox potential can subsequently induce the regulated release of chemical medicines both in vitro and in vivo, hence leading to a more effective anti-tumor effect. Sun et al. developed a novel polyethylene glycolated liposome (ES-SSL-OXA) that specifically targeted estrogen receptors. This liposome was loaded with oxaliplatin, leading to enhanced metabolic characteristics, an improved safety profile, and increased effectiveness against tumors compared to conventional oxaliplatin formulations.^389^ Yu et al. made the butyrate-modified NPs that were individually encapsulated with sorafenib and salinomycin. Butyrate, a multifunctional ligand, was found to facilitate transcytosis by interacting with monocarboxylate transporter 1 (MCT-1), which was found to be highly expressed on the surface of hepatocellular carcinoma (HCC) cells.^390^

Nanotechnology can effectively overcome the problems of low bioavailability, low efficacy, and toxicity of traditional chemotherapy drugs.^391–393^ FOLFOX, a combination of folic acid (FnA), 5-fluorouracil (5-FU), and oxaliplatin (OxP), is a standard drug for the treatment of colorectal cancer. Guo et al. prepared PEGylated lipid nanoparticles (Nano-Folox) targeting aminoethyl anisidine by nanoprecipitation technology, which were composed of the active forms of OxP ([Pt (Dach) (H_2_O)_2_] ^2+^) and FnA. The results showed that PEGylated nanoparticles enhanced the blood circulation of Pt by about 10-fold. The encapsulation efficiency (EE) and loading content (LC) of the NPs were about 99% and 67 wt%, respectively. Through the drug uptake experiment in vitro, higher Pt uptake was achieved in CT26-FL3 cells. In the in situ CRC mouse model, the Pt tumor accumulation (~40% ID/g) achieved by nano Folox targeting AEAA was remarkably higher than that of its non-targeted counterpart (~23% ID/g). The combination of nanoFolox and 5-FU exhibited a more potent chemotherapeutic effect while remaining non-toxic in comparison to FOLFOX.^394^ Irinotecan is an important chemotherapeutic drug for colorectal cancer and pancreatic cancer, but it has bone marrow and gastrointestinal toxicity.^395^ Although Onivyde (the irinotecan liposome) has been used in patients with PDAC, the toxicity of Onivyde still needs to be solved in the treatment of colon cancer. In a recent study from Ning et al., irinotecan loaded with lipid bilayer (LB)-coated MSNPs was studied, showing efficient drug delivery. Compared with free drugs and Onivyde, MSNPs showed higher efficacy and reduced bone marrow and gastrointestinal toxicity.^396^ Li et al. reported that the hydrophobic antitumor drug docetaxel (Dtxl) was loaded into the 125-nm-diameter polyethylene glycol silica nanotubes (SN-PEG) by electrostatic adsorption. The IC_50_ in vitro was only 7% of that of free Dtxl. Compared with Taxotere, SN-PEG-Dtxl showed stronger antitumor activity.^397^

Single chemical drug therapy is easy to produce drug resistance, and the treatment effect is not favorable. Nanocarriers can realize the synergetic chemotherapy of multiple drugs, reduce drug resistance, improve the pharmacokinetics of drugs in vivo, and enhance the anti-tumor effect.^380,387,398–400^ Albumin paclitaxel combined with gemcitabine is one of the recommended regimens for the treatment of pancreatic cancer, according to NCCN guidelines in clinic.^401^ Chen et al. designed a micelle for the co-delivery of paclitaxel and phosphorylation of gemcitabine based on the ethylene glycol-polyarginine-polylysine (PEG-*p*Arg-*p*Lys) platform. The AE105 peptide can specifically bind to the urokinase-type plasminogen activator receptor (uPAR), which is overexpressed in tumors and stromal cells. The micelle is modified by the AE105 peptide and a pH-sensitive molecule (2-propan-3-methylmaleic anhydride, CDM) to achieve targeting and a pH response. The micelles exhibited stability within the outer layer of the tumor, which possessed a higher pH value. In the tumor with a lower pH value, the micelles were decomposed to release paclitaxel to destroy the internal tumor matrix and phosphorylated gemcitabine to kill pancreatic cancer cells. Meanwhile, the relatively complete external matrix reduced the tumor metastasis, and the liposome has the ability to modulate the immunosuppressive microenvironment of tumors by enhancing the population of cytotoxic T cells and restricting the proportion of T regulatory cells.^402^ Cancer-associated fibroblasts (CAF) can remodel tumor extracellular matrix, resulting in low permeability and drug resistance to traditional drugs. Chen et al. prepared a novel FH-SSL-Nav liposome targeting the tumor matrix. FH is a peptide of small size that has strong affinity for tenascin C (TNC), a protein released by CAF. On the other hand, Navitoclax (NAV) is a tiny molecule with targeted and high affinity properties, capable of selectively inducing apoptosis in CAFs. The FH-SSL-Nav liposome can enhance the infiltration of hepatocellular carcinoma cells, down-regulate the deposition of ECM, reduce tissue fluid pressure (IFP), and promote blood perfusion. Combined with Nav and DOX therapy, the results revealed that the FH-SSL-NAV model could significantly improve the inhibitory effect of liposome adriamycin (7PEP-SSL-DOX) on hepatocellular carcinoma and partially reverse the microenvironment-induced chemotherapeutic drug resistance.^403^

#### Gene therapy

Tumor gene therapy is a focus of current attention, including restoring or enhancing gene function, reducing abnormal gene pathogenicity, inhibiting the expression of some genes, enhancing immunity, and reducing the risk factors of disease. Many forms of gene therapy have been explored for the treatment of digestive system tumors.^404–407^ The vector of gene therapy is regarded as important for gene therapy. The traditional virus vector has some security problems, such as immunogenicity and mutation. The non-viral vector based on nanotechnology overcomes the shortcomings of the viral vector and can achieve a higher curative effect through modification.

Safe and effective gene repair can be realized through nano-mediated tumor suppressor gene delivery, and the corresponding anti-tumor effect can be achieved. Kim et al. synthesized galactosylated a polyethylene glycol chitosan-grafted spermine (GPCS) copolymer by using the amide bond between galactosylated polyethylene glycol and melamine to load the programmed cell death protein 4 (PDCD4) gene, namely the GPCS/DNA complex. The particle size distribution of GPCS/DNA complex was uniform, and further in vitro and in vivo transfection studies showed that GPCS/DNA complex had higher transfection efficiency than PCs/DNA complex. The high level of GFP expression in liver tissue indicates that the specific ligand–receptor interaction between the galactose part of the GPCS copolymer and ASGPRs may effectively transfer the tumor suppressor gene PDCD4 to hepatoma cells.^408^ In light of the pronounced upregulation of vascular endothelial growth factor receptor (VEGFR) in HCC and the positive correlation between up-regulation of AChE expression and apoptosis, Liu et al. coupled YC21 targeting EGFR to a PC vector composed of β-cyclodextrin and PEI600 to form the EGFR targeting gene vector YPC. The YPC vector has high gene transfer ability to EGFR-positive hepatoma cells, promoting AChE gene expression and significantly inhibiting liver cancer in vivo and in vitro by inhibiting *p*-ERK and cyclin D1.^409^ Activated hepatic stellate cells (AHSC) promote the activation of immunosuppressive cells such as M2 and MDSC and form a matrix barrier to restrict the migration and function of T cells through CXCL12/CXCR4 and various growth factors such as TGF-β, which creates a favorable environment for tumor growth. Intrahepatic relaxin (RLN), an anti-fibrosis peptide, can inhibit the activity of aHSCs and alleviate liver fibrosis. Hu et al. used liposome calcium phosphate nanoparticles (LCPs) modified by aminoethyl anisamide (AEAA) targeting the sigma-1 receptor to deliver the RLN gene into tumor and reverse the inhibitory microenvironment. In the liver metastasis model of colorectal cancer and pancreatic cancer, pRLN LCPs nanodelivery gene therapy can significantly inhibit the progression of tumor metastasis, prolong the survival time, and reactivate the immune internal environment.^406^

Gene therapy can also achieve therapeutic effects by silencing tumor overexpression genes or oncogenes. Research has been focused on gene therapy that can deliver siRNA *via* nanocarriers. Pancreatic cancer exhibits a significant upregulation of the nerve growth factor (NGF) gene. Accordingly, Lei et al. prepared a novel fluorescent gold nanocluster (GNC) through a one-step reaction. The GNC particle size was 2.6 ± 0.5 nm, and it had a positive surface charge of 19.9 ± 0.8 mV. The NGF siRNA was adsorbed to form a GNC-siRNA complex with 16.6 ± 3.0 nm through electrostatic interaction. X-ray photoelectron spectroscopy (XPS) and electrophoretic mobility change analysis found that siRNA was successfully bound to GNC. Furthermore, the GNC siRNA complex has demonstrated significant efficacy in safeguarding siRNA molecules from destruction by serum nucleases. In vitro studies have shown that GNC siRNA can protect siRNA from degradation, promote cell uptake, and escape from the lysosome into the cytoplasm, thus achieving effective siRNA-mediated gene silencing. Concurrently, in vivo studies showed that GNC could extend the duration of siRNA circulation within the bloodstream while also augmenting the concentration of siRNA specifically within tumor tissues via the EPR effect. In the subcutaneous pancreatic cancer model, compared with the saline control group, the tumor volume of mice treated with GNC-siRNA decreased by 52%, and the NGF mRNA of mice treated with GNC-siRNA decreased by 69%. In the pancreatic cancer in situ model, the tumor volume in the GNC-siRNA treatment group was also the smallest. Compared with the saline control group, the GNC-siRNA group significantly reduced the expression of NGF mRNA in the tumor in situ. All these results indicated that the GNC siRNA complex could enhance the knockdown of a specific NGF gene in the pancreatic tumor, which can effectively inhibit tumor growth in the pancreatic tumor model without adverse effects or toxicity.^410^ Cancer-associated fibroblast (CAF) is an important part of the tumor microenvironment. By secreting growth factors, stimulating angiogenesis, altering the extracellular matrix, and inhibiting the anti-tumor immune response, it can facilitate tumorigenesis. Some studies have found that IL-6-mediates the interactions between gastrointestinal tumor cells and CAF by promoting the activation of fibroblasts.^411^ Salimifard et al. delivered IL6 specific siRNA through hyaluronic acid PEG chitosan lactate (H-PCL) nanoparticles (NPS), which could inhibit the proliferation and metastasis of CT26 cells and inhibit cancer progression.^412^

#### Immunotherapy

The challenges of effectively treating tumors using immunotherapy arise from the immunogenic nature of tumor cells and the immunosuppressive characteristics of the tumor microenvironment. The combination of nanotechnology and immunotherapy can enhance the sensitivity of tumor to immunotherapy.^413–417^

A therapeutic tumor vaccine is a method to induce antigen-specific adaptive immunity against tumors. A TLR agonist, a kind of immune adjuvant, has been used in clinical cancer treatment. However, its low solubility and poor pharmacokinetics limit its application in cancer immunotherapy.^418–421^ Ni et al. designed a novel double adjuvant antigen nano-vaccine (banNVs) for colon cancer therapy research.^422^ Firstly, the primer-PEG-PLA micelles were synthesized in two steps. The micellar and CpG-coding template DNA were subjected to a nanometer template RCR reaction to self-assemble into PEG-PLA and CpG polymer core-shell structure particles loaded with hydrophobic R848. Then, the cationic PEG-g-PPT was used to shrink the particles, while PPT was loaded with hydrophobic new antigens to form banNVs, realizing the synergistic delivery of peptide neoantigens (Adpgk), Toll-like receptor 7/8 (TLR7/8) agonist R848, and TLR9 agonist CpG. Antigen presentation and APC stimulation in vivo and in vitro have shown that banNVs might increase the expression of costimulatory factors CD80 and CD86 on DC and promote the intracellular delivery and release from endocytosis continuously of antigen and adjuvant, which led to persistent antigen presentation and DC maturation. Antigen-specific T cell response studies showed that the frequency of Adpgk+ induced by banNVs treatment was increased by 5.6 times, and CD8+ T cells were enhanced. Systemic T cell response and immunogenicity were enhanced in vivo, which was beneficial to achieve effective and lasting immunotherapy. Vaccines are also a fine choice for liver cancer treatment. To achieve the efficacy of a complete cancer vaccine, it is necessary to effectively deliver cancer antigen and immune enhancer at the same time, prevent off-target strong release, and maintain good biocompatibility. Li et al. prepared an Ms@Mof cancer vaccine containing OVA antigens and immune enhancer polyI:C, which is targeted and pH-responsive and can be efficiently delivered to the lymph nodes to enhance its availability and minimize off-target effects. Combined with the anti-PD1 antibody, it showed a synergistic effect, reversed immunosuppression of the tumor microenvironment, and was effective in long-term anti-tumor therapy.^423^

Cytokine is a common immunotherapeutic agent, yet its clinical application is limited by its toxicity, such as IL12. The optimization of the NP carrier can improve its application to some extent.^424,425^ In a recent paper, Barberio et al. prepared PLE IL-12 NPs by layer-by-layer (LBL) assembly, which can effectively encapsulate and release cytokines. The toxicity of PLE-IL-12-NPs was observed in healthy C57BL/6 mice. The results showed that the mice without carrier IL-12 lost weight rapidly during the administration, while the mice treated with PLE-IL-12-NPs changed barely. PLE-NPs reduced the toxicity of IL12, enhanced the activity of tumor lymphocytes, and slowed down the growth of colon cancer.^426^ Xu et al. used tripolyphosphate (TPP) as the coagulated cross-linking agent to form CS-TPP/IL-12 NPs loaded with IL12. With the increase in the weight ratio of chitosan/TPP, the average diameter of NPs was 178–372 nm. In contrast to the mice who received IL-12 treatment, the mice treated with CS-TPP/IL-12 exhibited a notable reduction in liver enzymes, namely ALT and AST. Furthermore, no evident pathological alterations were observed in the heart, liver, spleen, lung, or kidney of these mice. It can be concluded that CS-TPP can shield the toxicity of IL-12 in the process of circulation in mice. Subsequent investigations conducted in vivo have demonstrated that the CS-TPP/IL-12 complex effectively suppresses the liver metastasis of colorectal cancer (CRC) through the facilitation of NK cell infiltration and the recruitment of certain T cell populations.^427^ In addition to IL12, tumor immunotherapy mediated by nano-carrier delivery has many other cytokines, such as IL15, tumor necrosis factor-associated apoptosis-inducing ligand (TRAIL), transforming growth factor (TGF), TNF, etc.^428–432^

According to the characteristics of digestive system tumors, different immunotherapies have been used in anti-tumor research. CRC that is microsatellite-stable exhibits resistance to immunotherapy. Studies have found that quercetin (Q) and malonate lactone (A) in the molar ratio of 1:4 (Q: A) can synergistically induce immunogenic cell death (ICD). To achieve proportional delivery of Q and a for colon cancer, Zhang et al. prepared QA-M micelles using 1,2-distearyl-sn-glycerin-3-phosphate ethanolamine-n-methoxy-poly (ethylene glycol 2000) (DSPE-PEG2000) and *D*-α-tocopherol polyethylene glycol succinate (TPGS). The average diameter of the micelles was 20 ± 0.6 nm, and their distribution was relatively narrow. The entrapment efficiency of the micelles was more than 90%. QA-M micelles could remarkably inhibit the tumor of colon cancer in situ in mice. The reactivation of anti-tumor immunity was achieved by the induction of ICD, which resulted in cytotoxic effects and the regulation of the immunosuppressive tumor microenvironment.^433^ Goodwin et al. used lipid calcium phosphate (LCP) NPs to co-coat phosphorylated adjuvant 5’pppdsRNA, RIG-1 ligand, and CRC model phosphorylated tumor-specific peptide antigen (p-AH1-A5). In the CT-26 FL3 in-situ CRC liver metastasis model, the growth rate of primary colon cancer was significantly slowed, and the formation of liver metastasis was inhibited. Meanwhile, the vaccine component 5’pppdsRNA adjuvant enhanced the CD8^+^ T cell population without increasing the immunosuppressive cell type, such as T-regulatory cells or myeloid-derived inhibitory cell population.^434^ The liver is a natural immune tolerance organ and is more severe under the conditions of liver disease and inflammation. Ceramide is a sphingolipid metabolite that can affect T cell signal transduction and induce the apoptosis of cancer cells. However, its hydrophobicity and poor cell permeability limit its application. Li et al. designed C6-ceramide nanoliposomes (LipC6), which enhanced the cell permeability of ceramide. The findings from the in vivo mouse model demonstrated that the administration of LipC6 effectively suppressed tumor growth by decreasing tumor cell proliferation and Akt phosphorylation and promoting tumor cell apoptosis. LipC6 could also increase the activity of CD8^+^ T cells, reduce the number of tumor-associated macrophages, and alleviate TAM tolerance.^435,436^ Hepatic sinusoidal endothelial cells (LSEC) can lead to immune tolerance in the liver. Melittin, a natural cationic host defense peptide, has unique LSEC targeting capability, tumor cytotoxicity, and immunoregulatory effects. However, melittin is highly toxic and cannot be intravenously injected. The α-peptide-NP drug delivery platform solves the problem of melittin delivery, regulates hepatic sinusoidal endothelial cells (LSECs), breaks the inherent tolerance of the liver, and activates LSECs that can mediate immune cell recruitment and reverse the inhibition of other immune cells.^437^ In view of the poor immunogenicity of pancreatic cancer, Li et al. constructed a mixed micellar delivery system targeting M2 tumor-associated macrophages, which specifically blocked PI3K=γ and colony stimulating factor-1 receptor (CSF-1R) pathways. The micellar can activate the anti-tumor immune response through a dual mechanism, including reducing the tumor inhibition signal and the infiltration of MDSC and remodeling the immunosuppressive microenvironment into the immune active microenvironment, which improved the immunotherapeutic effect of pancreatic cancer.^438^

#### Combining therapy

In order to achieve better therapeutic effects and reduce toxicity, most reported studies use multi-functional drugs to treat tumors. Nanotechnology provides a broad platform and selectivity for the combination of multiple drug delivery methods, which contributes to the optimization of drug delivery schemes, the realization of complementary advantages, and the enhancement of the anti-digestive system tumor effect.^394,439–442^

The integration of chemotherapy with gene therapy is a very efficacious and pioneering approach in the treatment of cancer. siRNA can not only inhibit cancer itself by silencing the oncogenes but also enhance the sensitivity of cancer cells to chemotherapy, thus improving the overall therapeutic effect. Li et al. developed lactic acid (LA)-modified redox reactive NPs (LA NPs) to deliver 10-hydroxycamptothecin (HCPT) poly and sibcl-2 RNA for synergistic anti-hepatoma. The size of the NPs was around 85 nm, and the encapsulation efficiency of siRNA was 80%. Through LA-specific recognition of the overexpressed ASGP receptor on HCC cells, the NPs could accumulate in the tumor tissues and target HCC cells. The highly reducing GSH catalyzed the prodrug transformation in the liver tumor microenvironment, where the hydroxyl camptocamptoid molecule and siBcl-2 were rapidly released. Apoptosis induced by HCPT and siBcl-2 silenced anti-apoptotic genes coordinated to increase apoptosis and inhibit tumor growth.^443^ The poor prognosis of esophageal cancer is related to the lack of targeted treatment. The combination therapy of nano targeted delivery systems can provide a novel way for the therapy of esophageal cancer. A new nanocarrier EYLN designed by Jun et al. was loaded with the chemical anticancer drug adriamycin and a small interfering RNA targeting LPCAT1, which was highly expressed in esophageal cancer, to form EYLN-DOX/siLPCAT1. EYLN-DOX/siLPCAT1 was coated with proinflammatory leukocyte membrane to obtain mEYLNs-DOX/siLPCAT1, which was easy to internalize by esophageal cancer cells, target tumors, and significantly inhibit the proliferation and migration of esophageal cancer cells. The inhibitory efficacy of EYLNS-DOX was shown to be superior to that of the original DOX formulation. Moreover, the introduction of siLPCAT1 was observed to significantly augment the tumor inhibition capabilities of EYLNS-DOX. Thus, the combination of chemical and gene therapy achieved higher efficacy.^444^

**Fig. 2:**
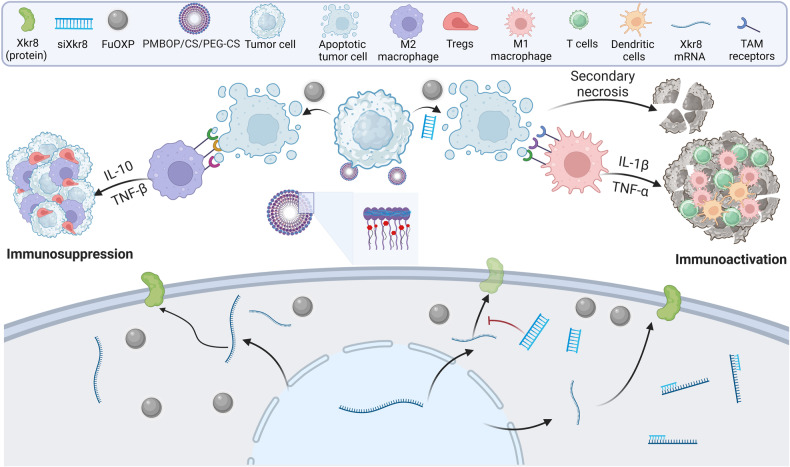
The proposed strategy aims to counteract the effects of chemotherapy drugs, specifically the induction of Xkr8 and the resulting immunosuppression. This reversal strategy involves delivering siXkr8 directly to the affected site, simultaneously with the chemotherapy drugs. This approach not only targets the specific gene responsible for these effects but also utilizes the synergy between siXkr8 and the chemotherapy drugs to achieve a more effective therapeutic outcome. (Created with BioRender.com) Reproduced with permission from Chen et al.^681^, Copyright 2022 Springer Nature Limited

Sorafenib is a molecularly targeted drug used to treat HCC by oral administration. Nevertheless, the therapeutic efficacy of Sorafenib is still inadequate. Yu et al. developed an oral delivery platform consisting of nanoparticles that were modified with butyrate. These nanoparticles were individually loaded with sorafenib and salinomycin. In contrast to regular hepatocytes, the expression of monocarboxylate transporter 1 (MCT-1) was found to be significantly elevated on the surface of HCC cells. In addition, butyrate exhibited an interaction with MCT-1, hence aiding in the process of transcytosis. Therefore, this platform demonstrated the ability to enhance transepithelial transport in the intestine, facilitate drug accumulation in the liver, and promote HCC cell uptake through MCT-1-mediated endocytosis. The efficacy of this combination approach was established through in vivo research conducted on an orthotopic HCC model. These experiments revealed that the strategy generated substantial damage through ferroptosis and stimulated a robust systemic immune response, ultimately resulting in effective tumor elimination.^390^ Although immunotherapy has been widely used in the treatment of cancer, certain in vivo and in vitro factors, such as myelosuppressive cells (MDSC) and tumor-associated macrophages (TAMS), can cause immune escape and make tumors resistant to immunotherapy.^445–450^ Immunogene therapy can suppress factors related to immune escape, improve the immunosuppressive environment of tumors, and enhance the immunotherapy of tumors.^451–453^ Chen et al. demonstrated that the administration of chemotherapeutic drugs resulted in the upregulation of Xkr8 gene expression at the transcriptional level in both in vitro and in vivo. Xkr8 is a protein that functions as a scramblase and is known to be activated in the context of apoptosis. The researchers utilized the aforementioned finding to create a nanocarrier capable of simultaneously delivering Xkr8 short interfering RNA (siRNA) and FuOXP, which is a prodrug combination consisting of 5-fluorouracil and oxoplatin. The intravenous injection of the nanocarrier demonstrated significant inhibition of tumor development in both pancreatic and colon cancer models, accompanied by an augmented immune response targeting the tumors (Fig. 2).^454^

Microenvironment formation in colon cancer is closely related to the intestinal microbiome and chronic inflammatory response. The intestinal microbiome can influence tumor immunotherapy.^455–459^ Lipopolysaccharide (LPS) is the product of intestinal Gram-negative bacteria, and LPS can activate colon cancer-related oncogenes, which are related to the immunosuppressive microenvironment.^460^ Based on nanotechnology, Wang et al. used LPS to trap plasmid selectively blocking LPS and lipid-protamine to compose LPD nanoparticles with a size of 140 nm and a surface charge of + 40.5 mV. After being injected into mice bearing CT26-FL3 tumors in situ through the caudal vein, LPS trap was highly expressed in the tumor. Meanwhile, CD8^+^, CD4^+^ T cells, MHCII^+^, and CD86^+^ DC increased, while MDSC decreased, indicating that the targeting of LPS has the potential to substantially enhance the functioning of DCs, the infiltration of T cells, and concurrently decrease the population of immunosuppressive cells. Combined with anti-PD-L1 therapy, more CD8^+^ and CD4^+^ T cells were produced, which extended the survival period of mice and enhanced the checkpoint blockade therapy of anti-PD-1/PD-L1 with no response to CRC.^461,462^ Huang et al. prepared a pH-responsive lipid of dendritic polymers-calcium phosphate (TT-LDCP) NPS modified with tumor targeting peptide (SP94), which carried the immunosuppressive factor of small interfering RNA (siRNA) and encoded immune stimulation cytokines IL-2 plasmid DNA (pDNA). TT-LDCP NP was capable of transferring siRNA and pDNA to hepatoma cells, thereby increasing the infiltration of CD8^+^ T cells into the tumor. This, in turn, reprogrammed the TME and inhibited the development of primary HCC and distant metastases. Combined with the HCC vaccine, TT-LDCP NP increased the sensitivity of HCC to the cancer vaccine.^463^

Phototherapy showed efficacy in the removal of lesions by non-invasive methods, employing either photothermal (PTT) or photodynamic (PDT) methods. They are widely used in the treatment of various cancers.^464^ PDT can induce apoptosis by promoting the formation of reactive oxygen species (ROS). It has space-time selectivity and low systemic toxicity. The use of phototherapy in gastrointestinal tumors is particularly advantageous due to the availability of gastrointestinal endoscopy and its tubular organ structure.^465^ However, in biological tissue, low light penetration leads to a low curative effect, while the accurate delivery of photosensitizer is also a problem.^466–469^ Nanotechnology provides a reliable way for photosensitizer delivery and PDT combined with other therapies (chemotherapy, gene therapy, and immunity) to enhance efficacy.^470–478^ Furthermore, using nanomaterials in photosensitizers makes them more soluble, bioavailable, targeted, stable, and lowers their toxicity.^479^

In a recent study, Hu et al. integrated β-cyclodextrin-grafted hyaluronic acid (HA-CD) with a heterodimer (NSP) crosslinked by a disulfide bond between photosensitizer pyrodemethylchlorophyll A (PPA) and 2,3-dioxygenase-1 (IDO-1) inhibitor NLG919 to form a multifunctional supramolecular nanocomposite (HCNSP). In this study, HA can target the CD44 receptor on the surface of colon tumor cells, and near-infrared (NIR) laser radiation can help release reactive oxygen species, which stimulated the infiltration of cytotoxic T lymphocytes in tumor cells with anti-tumor immunogenicity. Meanwhile, NLG919 inhibited IDO-1 and reversed the immunosuppressive tumor microenvironment. HCNSP realized photodynamic immunotherapy and IDO-1 blocking to synergistic suppress CT26 immunoreactive mice colorectal tumors. The hydrodynamic diameter of HCNSP was determined to be 48.2±0.6 nm, while its negative surface charge was found to be −29.7 ± 0.8 mV, and it possessed superior reduction sensitivity. Irradiation of CT26 cells with a 671 nm laser could effectively induce ROS production of HCNSP, inhibit tumor cell activity, and induce immunogenic cell death (ICD), CRT exposure, and DC cell maturation. The antitumor effect of HCNSP was further evaluated by a Balb/C mouse model of the CT26 tumor. The HCNSP treatment group showed significant tumor accumulation, inhibition of tumor growth, and prolongation of survival time. After treating tumors, analysis found that the tumor cells in the HCNSP+laser group were significantly apoptotic, accompanied by upregulation of IDO-1. For the immunoassay and distal HCNSP tumor effect analysis, the HCNSP+laser group showed an increase in tumor infiltration of T lymphocytes and only 12.6% tumor infiltration of Treg, which was 2.2 times lower than Treg tumor infiltration in the PBS group. Although the increase of IFN-γ secretion and IDO-1 expression induced by laser led to the highest ratio of Kyn to Trp in the HCNSP+laser group, the release of NLG919 resulted in a two-fold decrease in the ratio of Kyn to Trp, which effectively overcomes the immunosuppression of TME and increases the number of memory T lymphocytes, indicating that the synergistic PDT and IDO-1 inhibition could induce a strong immune response and an anti-tumor effect.^480^ Gao et al. prepared pH- and redox-responsive chemotherapeutic drug doxorubicin (DOX) and photosensitizer zinc (II) phthalocyanine (ZnPc) polymer micelles with a particle size of 160–180 nm and neutral charge. The polymer micelle was stable in the aqueous medium. When acting on liver cancer cells, the chemotherapeutic agent and photosensitizer produce synergistic cytotoxicity.^481^ Yuan et al. found that photosensitized NPs (mTHPC@VeC/T-RGD NPs) killed cancer cells under 660 nm near infrared (NIR) laser light, sensitized the tumor to PD-L1 blocking therapy, decreased primary and distant tumor growth, and created long-term host immunological memory to avoid recurrence.^482^ Similarly, Liu et al. created multifunctional nanoparticles (OIMH NPs) by synthesizing metal-organic framework MIL-100 (Fe) NPs and then modifying them with hyaluronic acid (HA), loading oxaliplatin (OXA), and indocyanine green (ICG). The OIMH NPs combined chemotherapy and PTT to kill tumor cells and sensitized them to PD-L1 checkpoint inhibition.^483^

### Diagnosis and treatment integration

The unique and excellent performance of nanotechnology can realize multi-material loading and integration of diagnosis and treatment. The therapeutic drugs and various contrast agents can be encapsulated in nanoparticles at the same time, which can realize the imaging diagnosis and visual treatment effect.^484–488^ Jing et al. developed a nanoprobe based on extracellular vesicles, which have a high affinity for tumor cells. This nanoprobe was used in combination with PET, CT, and NIRF imaging to clearly visualize in situ colon cancer models for guided surgery. Tumor location and margins in mouse tumor resection can be clearly seen with real-time NIRF imaging.^489^ Aiming at the T cell immune resistance induced by the interaction between programmed PD-L1 and PD-1 on T cells in gastric cancer, Luo et al. combined folic acid (FA) and disulfide (SS)-polyethylene glycol (PEG)-conjugated polyethyleneimine (PEI) with a superparamagnetic iron oxide nanoparticles (SPIONs) composite as a PD-L1 siRNA delivery system, which could effectively target PD-L1 in gastric cancer and be used as a T2-weighted contrast agent for cancer MRI.^490^

Diagnostic imaging can also be combined with phototherapy. Zhang et al. prepared amphiphilic JNP NPs that delivered hydrophilic drugs (DOX) and hydrophobic drugs (docetaxel) simultaneously. The nanoparticles had independent pH and near-infrared sensitivity, which was conducive to controlled drug release. Meanwhile, AuNC and Fe(OH)_3_ nanomaterials had the ability of CT/MR imaging and could cause the benefit of chemical photothermal therapy under near-infrared laser irradiation, which led to multiple effects to significantly inhibit tumor growth.^491^ Zeng et al. designed Au@MSNs ICG, which was a kind of MSN based on NIR response PTT and loaded with the imaging diagnostic agent indocyanine green (ICG). For hepatoma cells, the NPs had stable fluorescence, biocompatibility, and NIR/CT signals. The NPs could produce a fine temperature response under NIR laser irradiation, which was conducive to the precise treatment of tumors.^492^ Qiu et al. developed a drug delivery system called arsenic trioxide (ATO)/PFH NPs@Au-cRGD. This system was designed to function as a nano-ultrasound contrast agent, enabling the synergistic utilization of ultrasound imaging and immune stimulation through ferroptosis and PTT.^478^

## Nanomedicine in lung cancer

Lung carcinoma is a type of malignant neoplasm that has a significant incidence and an unsatisfactory mortality rate. With a five-year survival rate of approximately 19%, lung cancer is responsible for over 1.8 million deaths annually worldwide, making it the leading cause of cancer-related fatalities. This poses a significant threat to human life and well-being.^1,358,493–495^ During the initial phase, lung cancer typically does not exhibit any symptoms. The symptoms will occur gradually as their illness progresses, including cough, hemoptysis (hemoptysis), blood in the sputum, dyspnea, asthma, weight loss, fatigue, and dysphagia. Based on the histological features of cancer cells, lung cancer can be categorized into two primary forms: small-cell lung cancer (SCLC) and non-small-cell lung cancer (NSCLC).^495–497^ The molecular mechanism of lung cancer has a complex molecular basis. It is controlled by many genetic and epigenetic parameters. Any DNA damage will stimulate the transformation of lung epithelial cells into tumor cells. Therefore, it is a challenge to accurately understand which mutations are the main cause of lung cancer. Genomics suggests that the variation in lung cancer arises from the alteration of particular biological compounds. V-Ki-ras2 Kirsten ratsarcoma viral oncogene homolog (KRAS) and epidermal growth factor receptor (EGFR) were the most frequent mutations observed, along with p53, Kelch-like ECH-associated protein 1 (Keap1), serine/threonine kinase 11 (STK11), and neurofibromatosis 1 (NF1) as tumor suppressor genes.^498,499^ The expression of EGFR is significantly high in over 70 to 80 percent of NSCLC, making it a crucial focus for the treatment of lung cancer.^500–502^

Currently, the management of lung cancer primarily involves surgical intervention, the administration of chemical drugs, and radiation therapy.^503–506^ The primary option for treating lung cancer is surgical resection, and patients with early-stage lung cancer have a higher five-year survival rate following surgical resection. However, lung cancer patients are usually diagnosed when it is already in the late stages, and the survival rate of patients is reduced due to ineffective treatment. Patients with advanced lung cancer mainly receive chemotherapy and radiotherapy. The chemotherapy drugs used in clinical practice primarily consist of four main categories of cytotoxic agents. The first category is alkylating agents, such as cisplatin and carboplatin, which directly damage DNA and disrupt its replication and transcription. The second category is antimetabolites, including pemetrexed and gemcitabine, which can interfere with the synthesis of nucleic acids. The third category is topoisomerase inhibitors, such as topotecan and etoposide. Lastly, the fourth category is spindle poisons, including vinorelbine, paclitaxel, and docetaxel, which impact the polymerization of microtubules in the mitotic spindle. Platinum salts are the mainstay of NSCLC treatment among these medications. Nevertheless, conventional medications possess drawbacks such as drug resistance, a limited half-life period, and numerous adverse effects (like kidney damage, heart toxicity, blood disorders, and gastrointestinal reactions), significantly constraining their usage in clinical applications. Hence, it is crucial to devise novel approaches for early diagnosis in order to prolong survival time among individuals with lung cancer. Additionally, the discovery of alternative therapeutic strategies that minimize adverse reactions and maximize treatment effectiveness is imperative.^507,508^

Over the past few years, the gradual advancement of nanotechnology has overcome the limitations of conventional medications in managing lung cancer. Nanotechnology can be employed during chemotherapy to resolve the negative side effects and drug resistance associated with chemotherapy drugs. As a drug carrier, NPs have unique advantages in the treatment of lung cancer, including overcoming the physiological barrier, effectively delivering hydrophobic drugs, optimized-release drugs, and targeting tumor tissue specifically to enhance the accumulation of drugs at the target sites.^500^

### Diagnosis

Clinically, bronchoscopy is a useful method for assessing lesions in the central airways, but it is an invasive procedure that can only examine a few upper bronchi.^509^ According to the guidelines, the clinic imaging diagnoses for lung cancer include CT, MRI, single-photon emission computed tomography (SPECT), and positron emission tomography (PET). These are no-doubt powerful noninvasive diagnostic tools. However, weaknesses of these techniques are obvious, such as noneffective detection for small lesions, short blood circulation time of the contrast agent, non-specificity to the target tissue, and high concentration-induced nephrotoxicity.^510,511^

Furthermore, the biomarker is an essential diagnostic tool for lung cancer. As molecular biology and related technologies develop, more and more tumor biomarkers are discovered. People in good health and those in the early stages of disease exhibit little to no sign of these markers, indicating the presence of malignant tumors only when the concentration of serum tumor biomarkers rises. Recently, the molecular biomarkers utilized in clinical settings primarily consist of autoantibodies, complement fragments, microRNAs, circulating tumor DNA, blood proteins, etc. The value of circulating tumor DNA and proteins as a biomarker in advanced tumor diagnosis has been confirmed.^512^ Nevertheless, the efficacy of their use to diagnose early-stage lung cancer remains uncertain.^513^

Nanotechnology gradually improves the development of tumor diagnosis, and provides influential breakthroughs in medicine and healthcare. NPs have a variety of properties that allow them to overcome the biological and chemical barriers of the human body, increasing therapeutic efficacy while reducing invasiveness and optimizing biocompatibility.^514^ For instance, the rapid development of nanomaterials in imaging substances exceeds the constraints of contrast agents frequently used in clinical settings. NPs provide substantial advantages for CT/MR imaging, such as improved enhancement, increased specificity for tumor lesions, and enhanced imaging accuracy when compared to conventional CT or MR contrast agents.^515^ Thus, it is essential to continue developing low-cost, non-invasive diagnostic methods for the early identification of the disease. This is now possible due to the swift advancement of nanotechnology.^516^

#### Nanotechnology-based biomarkers

Biomarkers linked to tumors can be found in the bloodstream through liquid biopsy, which presents a significant opportunity for early detection of lung cancer. Nanoscaled extracellular vesicles (EVs), such as exosomes, are small vesicles released by various cell types and are highly present in bodily fluids.^517^ To be more precise, exosomes isolated from lung cancer tissue include molecules linked to tumors, such as chemokines, different microRNAs, and the epidermal growth factor receptor (EGFR). These exosomes play a crucial role in facilitating cellular communication and have significant involvement in the development of tumors.^518^ Hence, investigating particular biomarkers obtained from EVs in the bloodstream can offer valuable insights that are challenging to obtain through medical imaging,^519^ making it a rising method in the clinical detection of lung tumors. Recently, it has been discovered by scientists that exosomal miRNAs, such as miR-205, miR-19a, miR-19b, miR-30b, and miR-20a, experienced a decrease in the bloodstream following surgical removal. These compounds could potentially serve as valuable indicators for diagnosing squamous cell lung carcinoma. Potential biomarkers for the diagnosis of NSCLC include exosomal miR-126, miR-96, miR-23a, miR-30b/30c,^517^ miR-19b-3p, miR-21-5p, miR-221-3p, miR-584-5p, miR-425-5p, miR-409-3p,^520,521^ as well as lncRNA TBIL A, AG AP2-AS1,^522^ AHSG, and ECM1.^523,524^ Here we summarize the research progress of nanotechnology-based biomarkers explored in the diagnosis of lung cancer in recent years.

A high concentration of exosomes in the circulatory system is one of the noninvasive markers for early diagnosis of NSCLC, but there is no practical and accurate method to distinguish the normal and tumor cell exosomes. Fan et al. established a biosensor to accurately identify the origin of exosomes through between interaction of antibodies and the biological affinity of different recognition sites. Therefore, it provides a choice for high-throughput screening of biomarkers in clinical specimens.^521^ Yang et al. developed an immune biochip that can capture tumor-derived exosomes by using antibodies that target proteins associated with tumors. They can also measure the amount of exosomal RNA in the biochip using cationic lipid complexes that contain molecular beacons. The outcome demonstrated that the biochip can effectively distinguish tumor-derived exosomes from other exosomes and improve detection sensitivity and specificity with a short testing time.^525^ EVs contain a set of biomarkers under intense investigation. At present, the clinical utilization of EVs is restricted by troublesome and nonstandard separation methods and large-scale instruments. Liu et al. reported a simple and easy-to-use EV separation tool based on size that can help separate high-yield and high-purity EVs from biological fluids.^526^ The microRNA targeted biochip designed by Lee et al. can capture EVs and form large nanocomposites, promoting the combination of RNA and molecular beacons near the chip interface, thus improving the detection sensitivity.^527^

Cell-free DNA (cfDNA) is released by necrotic or apoptotic cells. Compared with cell-free DNA (cfDna), nanoscaled EV-derived DNA (nEV-DNA) is released from living cells, contains a relatively more complete fragment, and requires less plasma for detection. Wan et al. used PCR to examine nEV-DNA and cfDNA obtained from NSCLC patients and discovered a strong linear association between the quantities of nEV-DNA or cfDNA and tumor volume in the early stages of NSCLC. The sensitivity of nEV-DNA in the early diagnosis of NSCLC was 25.7% and 14.2%, while the specificity was 96.6% and 91.7%, respectively. These results suggest that nEV-DNA is superior to cfDNA in accurately identifying NSCLC at an early stage.^528^ Moreover, 60% of NSCLC patients are resistant to immunosuppressive therapy targeting T790M (a mutation of the EFGR site in DNA). So, non-invasive detection of the EFGR T790M mutation is necessary. However, there are too few cfDNA fragments detectable in circulation, so the sensitivity is low. Elena et al. put forward the combination usage of cfDNA and exosomal RNA/DNA (exoRNA/exoDNA), which can improve the diagnostic accuracy of lung cancer. By simultaneously capturing exosomal RNA/DNA and cfDNA, the sensitivity of detecting the T790M mutation achieved 92%, and the specificity achieved 89%.^529^ Krug et al. also demonstrated that exosomal RNA combined with cfDNA can improve the sensitivity of EGFR mutation detection in patients with NSCLC, especially in metastatic patients with low circulating tumor DNA levels; the sensitivity increased from 26% to 74%.^530^

Conventional analytical methods for biomarkers, such as Western blot, ELISA, and mass spectrometry, have complex reaction steps and complicated operations, thus limiting their clinical applications. For electrochemical technology, though it’s a technique with fast response, relatively low cost, and a wide range of detection, improvements are still needed for examining a wider range of biomarkers. Nanotechnology-based biomarker detection tools have been developed recently. Shoja et al. synthesized reduced graphene oxide (rGO), ordered mesoporous carbon (OMC), and Ni-oxytetracycline (Ni-OTC) NPs as the electroactive label, and fabricated the electrochemical geno-biosensor to detect the EGFR exon 21 mutation. This electrochemical biosensor possessed excellent sensitivity, satisfactory stability, and reproducibility.^531^ Besides, label-free electrochemical DNA sensing is the most important method to construct low-density DNA microarrays for clinical diagnosis. However, due to defects such as poor arrangement of DNA on the transducer surface and inconsistent data, label-free electrochemical detection suffers from poor selectivity and sensitivity. Divya et al. synthesized carbon-dot-stabilized silver NPs (CD-AgNPs), which are sensitive and specific for detecting low-density DNA. The use of short-chain DNA as a model system for label-free DNA sensing allows for effective differentiation of the hybridization state of the target DNA by altering the charge transfer resistance. This method is simple, rapid, and highly sensitive to molecular affinity reactions.^532^

In recent years, new technologies, such as photoelectric detection technology and surface-enhanced Raman spectroscopy (SERS), have become simpler and more sensitive than traditional biological detection methods.^533,534^ However, there are still some shortcomings. The development of nanotechnology provides a rich technical basis for the construction of multifunctional SERS nanoprobes. However, due to the heterogeneous composition of exosomes on the surface, even the exosomes secreted by the same cell may have uneven peaks in each spectrum. Shin et al. carefully analyzed the unique Raman bands of lung exosomes and tried to use principal component analysis (PCA) to learn the unique peaks of major patterns. The results indicated that several Raman bands had a strong association with the biomarkers of NSCLC, which are helpful for further study of exosomal biomarkers in cancer diagnosis.^535^ Moreover, Shin et al. explored the characteristics of SERS through machine learning and found a similarity between plasma exosomes. The classification accuracy was 95%, and the area under curve (AUC) was 0.912. The results indicated that deep learning combined with exosomal analysis is a potential method for early lung cancer liquid biopsy.^519^

#### Imaging diagnosis

Recently, NP-based contrast agents have been highly valued. Through modifications of NPs, limitations such as poor targeting and high toxicity could be partially solved. For example, the clinical use of CT imaging agents relies on iodine molecules that are quickly eliminated from the bloodstream by the kidneys because of their compact size. Iodine encapsulated with NPs can prolong the circulation time and reduce the required dose.^514^ Besides CT, nanotechnology can also overcome an important defect of traditional MRI contrast agents. Currently, paramagnetic gadolinium (Gd)-chelates used in clinical settings as MRI contrast agents have the risk of releasing free gadolinium ions and may result in the development of nephrogenic systemic fibrosis due to their potential toxicity. Gd NPs can reduce toxicity by preventing Gd^3+^ leakage. Thus, several gadolinium NPs have been developed, including Gd_2_O_3_, Gd-Si oxide NPs, and Gd-based silica NPs.^515^

Metal NPs, including gold, bismuth, and tantalum, have received increasing attention in the imaging diagnosis of cancer due to their steerable size and versatile surface chemistry characteristics. NPs can facilitate their targeting behavior towards the tumor by conjugating or attaching to biomolecules such as antibodies, aptamers, or peptides. Therefore, NPs are expected to effectively aggregate in the tumor sites by binding to specific ligands to enhance permeability and retention. However, NPs have not yet been utilized to maximal effect due to their surface properties and the non-negligible clearance of reticuloendothelial system (RES) organs.^536^ In order to solve these problems, different approaches were applied, and some progress was achieved. Perfluorocarbons (PFC) have the feature of high biocompatibility. Wu et al. have implied a PFC NP for MRI of lung cancer diagnosis in the rabbit model and found that paramagnetic PFC NPs increasingly accumulate in the primary lung tumors during the first 12-h post-intratracheal administration. Though it was not targeted, the accumulation and prolonged contact with tumor cells led to a similar result.^537^ Furthermore, pEGFR-targeted Ba2GdF7 NPs, a contrast agent that can be eliminated by the kidneys, were recently developed by Feng et al. By binding to EGFR-targeted peptides, this agent is intended to target tumors in MR/CT dual-mode imaging. Mouse model tests have confirmed that pEGFR-targeted Ba2GdF7 NPs with renal-clearing capabilities show good biocompatibility, effective kidney elimination, successful tumor targeting, and improved contrast enhancement.^538^

The unique configuration of positive and negative charges in zwitterions has led to the discovery of the possibility of producing antifouling NPs recently. The formation of a thick layer of hydration on the particle’s surface increases its colloidal stability and corrosion resistance, prolongs its half-life in the circulation, and protects it from immune system destruction.^515^ Recently, Wu et al. created Gd@C-dots NPs that were modified with Ac-Cys-ZEGFR: 1907. These NPs have a high-affinity probe for targeting EGFR, and their size can be controlled. They are also non-toxic and can be efficiently cleared by the kidneys without any leakage of free Gd^3+^ into the plasma or urine.^536^ Furthermore, there have been reports of the utilization of nanocomposites based on dendrimers as imaging agents due to their distinct structural features, controllable surface properties, and excellent compatibility with living organisms. According to Chen and colleagues, the targeting probe for NSCLC, Cy5.5-Gd-Au DENP-FA, has been synthesized for CT, MR, and optical tri-modal imaging. The active binding of folic acid (FA) to the folate receptors on tumor cells is likely responsible for promoting the uptake of nanoprobes. Hence, these nanoprobes exhibit positive compatibility with biological systems in vitro and in vivo and are primarily eliminated through the reticuloendothelial system and renal pathway.^539^

Although nanotechnology for the lung cancer imaging has been rapidly developed, nanoparticles used in clinical trials were demonstrated to induce fibrosis and asbestos-like scarring in the lung. Therefore, it is emphasized to adequately assess the underlying toxicological effects and the possibility of large-scale manufacturing of nanomaterials, which is also an important issue that should be solved in the future. In addition, many clinical studies are currently in the early stages, and the acute and chronic toxicity, detection range, depth, and reliability of these new materials need additional validation through clinical trials.^540^

### Treatment

#### Chemotherapy

Although chemotherapy can be effective in treating cancer, it can still be difficult to suppress the spread of drug-resistant cancer cells, which can harm healthy cells. Therefore, it is imperative to immediately investigate novel and effective therapeutic approaches for eliminating cancer cells and preventing the occurrence or spread of tumors. Drug delivery systems that use nanotechnology are accelerating the advancement of cancer treatment. The loading of small-molecule chemotherapeutic medications onto NPs for transportation enhances drug accumulation, improves the permeability and retention (EPR) effect, and greatly diminishes the adverse impact on healthy tissues. Here we summarize the recent advances in research on nanotechnology in lung cancer chemotherapy.

Platinum chemotherapy, followed by a second-line chemotherapy medication, is the standard treatment for the majority of patients with advanced NSCLC. The use of a therapeutic regimen containing cisplatin is commonly employed as a chemotherapy drug for treating different types of cancer, such as NSCLC. Cisplatin attaches to the DNA double-helix chain, disrupts DNA duplication, and hinders the production of biomolecules. Nevertheless, cisplatin frequently leads to significant toxicity, particularly nephrotoxicity, by stimulating the generation of substantial quantities of reactive oxygen species. Moreover, the utilization of cisplatin in conjunction with alternative chemotherapy medications has been employed for the treatment of NSCLC. Nevertheless, the effectiveness of this therapy is restricted due to the diverse methods of administration, resulting in patients’ inadequate adherence as well as the varying pharmacokinetics and physicochemical characteristics of the two medications. Recent research has attempted to explore solutions to these problems based on nanotechnology.

Curcumin, a naturally occurring antioxidant, shows promise as a means to boost the therapeutic impact of cisplatin by enhancing its anti-tumor metastatic activity through synergistic effects when combined with cisplatin. Through a series of sophisticated designs, Chen et al. developed a nanosystem that releases cisplatin and curcumin in response to changes in both redox potential and pH. This nanosystem is constructed using a disulfide link and phenylboronate ester and is coated with a polyethylene glycol (PEG) shell. This nanosystem possesses the capability to evade elimination by the reticuloendothelial system and sustain its stability inside the circulatory system. Upon reaching the tumor microenvironment, these chemical bonds may be disrupted, causing a change in the physical composition of the NPs, which leads to the quick release of the enclosed medicines. Both in vitro and in vivo experiments have shown that the nanosystem is better tolerated and has enhanced anti-tumor metastatic activity.^541^

Metformin is a common antidiabetic drug that has been shown to have significant anti-growth and pro-apoptotic effects in several cancers and is also used in combination with cisplatin to treat NSCLC.^542^ However, metformin needs to be administered orally, whereas cisplatin needs to be injected, leading to variable pharmacokinetic differences and reduced patients’ compliance. It is complicated to properly evaluate the synergistic effects of the two drugs, resulting in poor antitumor efficacy.^542,543^ To address these issues, Xiong et al. described a new approach for combining CDDP and metformin within a single self-assembled core-shell nanoparticle structure. The positively charged polymeric metformin (polymet) exhibits an electrostatic reaction with the negatively charged PGA-CDDP coupling to produce negatively charged nuclei, which are then anchored with PEGylated cationic liposomes. Experiments proved that the polymet-cisplatin NPs system successfully mitigated the nephrotoxic and weight-loss effects of cisplatin and significantly improved the targeting ability of drugs. Importantly, polymet-cisplatin NPs can be used in multiple doses without inducing drug resistance, which is not even possible when cisplatin is used alone.^544^

Demethyl cantharidin (DMC), an inhibitor of protein phosphatase 2A (PP2A), enhances the antitumor activity of DNA-damaging agents by specifically inhibiting PP2A without apparent toxicity. Previous studies have shown that cisplatin combined with DMC can be synergistic in cancer therapy. For combining two drugs and directly monitoring the fate of the drugs, Cong et al. designed a fixed DMC/cisplatin ratio, dual-sensitive, dual-drug backbone shattering polymer (DD-NP). Under an intracellular reducing/acidic microenvironment, DD-NP will be disintegrated in a chain-shattering manner, releasing dual synergistic drugs (cisplatin and DMC) in a precise (1:2) ratio to achieve the best anti-cancer effect. It has been proven that the use of DD-NP can significantly eliminate the tumor load. In addition, the high cisplatin content in the DD-NP can be directly tracked by Pt-based drug-mediated CT and ICP-MS.^545^

DOX can interfere with DNA replication and induce apoptosis in tumor cells. Previous studies have demonstrated that loading DOX and cisplatin into the same NP can achieve strong synergistic effects and effectively treat cancer. Xu et al. realized that the conventional approach to systemic medication administration presented numerous disadvantages, including inadequate tumor accumulation, limited effectiveness in inhibiting tumors, and significant toxic side effects. In comparison to systemic drug delivery, pulmonary drug delivery enables direct and efficient drug delivery to the lungs, resulting in lower dosage, higher efficacy, and reduced systemic side effects. This method avoids the liver-related first-pass effect by effectively controlling drug release and decreasing the frequency of drug administration. Xu et al. synthesized methoxy poly (ethylene glycol)-poly(ethylenimine)-poly(*L*-glutamate) (mPEG-OEI-PLG) copolymers, which loaded DOX by electrostatic interactions and loaded cisplatin by chelate interactions, respectively. The system could deliver DOX and cisplatin directly to the lung. In vitro experiments showed that Co-NPs were highly cytotoxic for tumor cells. In vivo experiments showed a high accumulation efficiency in the lung tumor, and rare aggregation in normal pulmonary tissue. In addition, Co-NPs exhibited superior antitumor efficacy against metastatic lung cancer compared to single treatment with DOX or cisplatin, and no significant adverse effects were seen throughout the course of treatment.^546^

Although several new approaches to treat NSCLC have been developed, chemotherapy for SCLC has not progressed satisfactorily, and the combination of etoposide (ETO) and platinum remains the mainstay of treatment.^547^ Drug co-delivery in nanosystems remains a challenge due to the differences in solubility and pharmacokinetics of different drugs. Wan et al. reported that a nanopolymer micelle, co-loaded with ETO and platinate, was used to coordinate the drug release ratio, which greatly increased the ratio of ETO to platinate. The combining effect can safely and effectively treat SCLC and NSCLC lung cancer.^548^ At the same time, there are many side effects of ETOs to treat SCLC, such as gastrointestinal reactions, leukopenia, thrombocytopenia, and alopecia. In addition, the pharmacokinetics of etoposide are unstable, and the therapeutic index is narrow, so there is an urgent need for a suitable drug carrier to overcome the inter- and intra-drug generation variability and improve the bioavailability. Although several NPs for chemotherapeutic drug loading have been developed to improve the bioavailability and anticancer efficacy of ETO, the cyclic metabolism and toxicity of NPs remain unsolved problems. To this end, Zhu et al. used the coprecipitation method to load ETO into pH-sensitive layered double hydroxide (LDH) nano-hybrids. The nano-hybrids could enhance the uptake of ETOs by lung cancer cells and induce apoptosis by inhibiting PI3K-AKT and ERBB signaling pathways, which increased the tumor suppression effect of ETOs by 3-fold. The nanosystem could also significantly reduce the hepatotoxicity and hematotoxicity of ETO as the laminar structure of LDH remained stable in plasma at pH = 7.4.^549^

Besides cisplatin, other chemotherapy drugs used for NSCLC therapy have also been explored in a better way based on nanotechnology. Docetaxel (DTX), a cell cycle-specific antineoplastic drug that inhibits microtubule depolymerization, has received approval as both first- and second-line therapy for NSCLC. However, the clinical administration of DTX can lead to severe side effects, such as hypersensitivity reactions, nephrotoxicity, and cardiotoxicity.^550^ In order to enhance the therapeutic efficacy of DTX, several nanocarriers, including PLGA, have been developed. Nevertheless, current PLGA-based nano-therapies commonly encounter challenges such as low plasma stability, premature drug release, inadequate tumor accumulation and retention, and inefficient uptake by tumor cells. Wu et al. reported an easy-to-fabricate HA-coated PLGA nano-docetaxel (DTX-HPLGA) system. Research has demonstrated that this system has notably potent anti-tumor capabilities and can decrease systemic side effects in comparison to free DTX. The application of a hyaluronic acid coating to PLGA NPs resulted in improved colloidal stability and increased specificity towards tumor cells, both in vitro and in vivo. The DTX-HPLGA exhibited an extended circulation period and showed a substantial increase in accumulation within the malignant lung tissues.^551^

Gemcitabine (GmcH), a potent chemotherapy drug, is widely utilized in the treatment of lung cancer to combat its growth. Nevertheless, GmcH faces challenges in its clinical utilization, such as limited duration in the bloodstream, inadequate pharmacokinetic and pharmacological effectiveness, and insufficient ability to infiltrate the intricate surroundings of lung cancer cells.^552^ In order to solve these challenges, Soni et al. employed the emulsification and solvent evaporation methods to load GmcH onto mannosylated solid lipid NPs (GmcH-SLNs). Additionally, they modified the surface of GmcH-SLNs by using the mannose ring-opening approach. The presence of mannose enhanced the drug loading rate, reduced drug release in the systemic circulation, decreased hemolytic toxicity, and allowed for a safer intravenous infusion of the drug. Furthermore, the presence of mannosyl facilitated the specific transportation of GmcH into lung tumor cells through the process of endocytosis. This led to a substantial enhancement in the toxicity of GmcH towards lung tumor cells while minimizing its harmful effects on normal cells.^553^

Photoluminescent semiconductor quantum dots (QDs) possess unique photochemical properties, such as sharper spectra and photostability. High-ZnO quantum dots (ZnO-QDs) are a cheap, low-toxicity, and pH-sensitive type of nanomaterial. Cai et al. developed a drug delivery method utilizing pH-sensitive ZnO-QDs to effectively load DOX. The system exhibited stability at a pH that is characteristic of normal bodily conditions, due to the safeguard provided by PEG. The system has the ability to attach to CD44, a protein that is abundantly present on the outer surface of cancer cells, in order to enhance the specific targeting effect. Due to the destabilization of ZnO-QDs in acidic intracellular environments, DOX can be precisely released within the cell. Furthermore, ZnO-QDs carriers can undergo complete biodegradation in the acidic environment of tumors and demonstrate anticancer action by selectively eliminating cancer cells. Hence, the synergistic effect of DOX and ZnO-QDs might greatly augment the process of cell death in cancer cells.^554^

Supramolecular drug-drug delivery systems (SDDDS), which involve the use of pure drugs through supramolecular interactions, present a hopeful approach for the treatment of cancer. Zhang et al. introduced an effortless approach to building SDDDS by combining the NSCLC medication gefitinib (GEF) and the tripeptide tyroservatide (YSV) using different intermolecular connections like hydrogen bonding and polyethylene glycol. Nanoparticles with consistent shape and size for drug administration were observed through transmission electron microscopy (TEM) and dynamic light scattering (DLS) when examining GEF and YSV self-assembly. Studies conducted in vitro and in vivo have demonstrated that SDDDS exhibits a superior ability to accumulate specifically in tumor tissues, resulting in remarkable therapeutic effectiveness without any notable adverse reactions. Significantly, the ready SDDD was uncomplicated and did not necessitate intricate chemical alterations, successfully evading unfavorable alterations in drug composition and characteristics.^555^

Although there are numerous benefits associated with lung administration, the utilization of inhaled nanoparticles in lung cancer treatment is restricted due to various factors, including lung dimensions, morphology, surface properties, and reduced metabolic function. The effectiveness of inhaled treatments for lung cancer relies heavily on (1) where and how much of the drug is deposited in the respiratory system; (2) the ability of the drug to pass through mucus in the airways; (3) the clearance of macrophages to avoid capture; and (4) the interactions with cells that are the intended target. Hence, the development of inhalable nanoparticles that can target specific cells in the tumor microenvironment or release therapeutic substances at precise locations is crucial. Conte et al. synthesized a novel redox-responsive PLGA-PEG NPs (RR-NPs). Upon entering the tumor microenvironment, this particular NP has the ability to adjust its surface properties, thereby improving the internalization of both particles and drugs by cells. The NPs demonstrated prolonged stability in the simulated biological fluids and a strong capacity to penetrate the artificial mucus layer, thanks to the external PEG coating. Simultaneously, the external covering of the NPs can be rapidly eliminated, thereby initiating the controlled release of the medication. Using RR-NPs, researchers have successfully developed a novel approach to improve the absorption of cancer cells with medication through the breakdown of proteins both inside and outside the cells, as well as the use of hydrophilic blocks to protect the cells.^556^ Mottaghitalab et al. created a silk fibroin NPs (SFNPs)-based inhalable delivery system for the efficient administration of gemcitabine (Gem) throughout the body. To target cancerous lung tissues, the researchers connected the SP5-52 peptide with Gem-loaded SFNPs. SFNPs enriched with specific gemstones demonstrated increased cytotoxicity against tumors, as well as enhanced cellular uptake and accumulation in lung tissue. According to the examination of tissue samples and imaging scans, animals that received targeted SFNPs demonstrated an increased rate of survival and showed no indications of metastasis.^557^

Epidermal growth factor receptor (EGFR), which is found in approximately 40% to 50% of cancer cells, including those in lung cancer, plays a crucial role in signaling pathways related to the metastasis, growth, and angiogenesis of cancer cells. Blocking the EGFR signaling pathway by inhibiting tyrosine kinase can result in the death of cancer cells.^558^ Hence, identifying effective drug delivery systems for EGFR signaling inhibitors is a crucial objective in the treatment of lung cancer. Erlotinib has the capability to inhibit the enzymatic function of tyrosine kinase and is effective in the treatment of various cancers, including NSCLC. However, the administration of this medication regularly leads to notable adverse reactions such as toxicity to living organisms, diarrhea, perforation in the gastrointestinal tract, reduced stability of the drug, and resistance to EGFR. Saravanakumar et al. have developed a pH-dependent chitosan NPs (CSNPs) loading with erlotinib and an adaptor AS1411 (APT) targeting the nucleolin receptor. By utilizing pH-sensitive and nucleolin receptor-targeting characteristics, the CSNPs have the ability to improve the absorption of erlotinib by cancer cells. This, in turn, facilitates the effective release of erlotinib into lung cancer cells, resulting in cancer cell death and ultimately enhancing the effectiveness of erlotinib for therapeutic purposes.^559^ Afatinib, another clinically approved drug targeting EGFR for treating lung cancer, is hydrophobic and has low bioavailability, making it easy to spread systemically and causing serious side effects. Cryer et al. proposed a new afatinib-gold NP (AuNP). It is formulated to enhance the efficacy and biocompatibility of afatinib. When tested on lung cancer cells in a laboratory setting, the killing effectiveness of the substance increased by 3.7 times, and it notably suppressed the growth of cancer cells. In addition to afatinib, this design offered a fresh opportunity for the advancement of unconventional AuNP pairings to be used with other molecules possessing therapeutic or diagnostic attributes. Furthermore, this design holds the potential for integration with photothermal therapy.^560^

EGFR resistance frequently occurs after 8–16 months when EGFR tyrosine kinase inhibitors like erlotinib and afatinib are utilized.^561^ Addressing drug-resistant lung cancer poses a significant obstacle in the realm of lung cancer treatment. Treating drug-resistant NSCLC can be achieved by using a combination of other chemotherapeutic agents along with tyrosine kinase inhibitors. Jeannot et al. reported a nanosystem with the ability to encapsulate both gefitinib, an inhibitor of EGFR tyrosine kinase, and vorinostat, an inhibitor of histone deacetylase, at the same time. It is a self-assembled NP with a 30-nm size consisting of a hyaluronic acid-based copolymer. The NPs, which are composed of hyaluronic acid, have the ability to carry both hydrophobic and hydrophilic chemotherapy drugs. They also offer targeted drug delivery through CD44 to avoid premature release of the drugs while in circulation. The drug-carrying nanoparticles triggered increased cancer cell apoptosis, and their antitumor effects in live organisms surpass those of free drug combinations. By applying these NPs on a weekly basis, the drug release is effectively regulated, leading to significant suppression of tumor growth while maintaining good tolerance.^562^ In order to achieve the optimal therapeutic outcome, additional experiments are required to enhance the drug combination’s concentration and the loading ratio of medications. Yang et al. created a pulmonary microsphere platform to concurrently administer afatinib and paclitaxel (PTX) for the treatment of NSCLC resistant to EGFR tyrosine kinase inhibitors. The authors loaded afatinib into stearic acid-based solid lipid NPs, and then co-loaded these NPs with PTX into poly-lactide-co-glycolide-based porous microspheres. Cellular studies demonstrated that the combination drug delivery system exhibited a remarkable therapeutic impact on drug-resistant NSCLC. The in vivo tests showed that afatinib and PTX in nanomaterials could remain in the lung at elevated levels for 96 hours, indicating the potential of this approach as a promising drug delivery method for the treatment of drug-resistant cancers.^563^

Biomimetic nanoengineering shows promise as a strategy for modifying the surface of nanoparticles in EGFR-mutated drug-resistant NSCLC. This approach involves using cell membranes derived from platelets, erythrocytes, leukocytes, and mesenchymal stem cells. Therefore, the nanoparticles that are coated with proteins associated with the membrane of cancer cells can penetrate into the cancer cells by binding to specific isotypes. Wu et al. created a bio-nanoparticle using a cancer cell membrane protein to combat drug-resistant NSCLC and improve the effectiveness of chemotherapy involving DOX and icotinib. In vitro, the bio-NPs demonstrated superior stability and enhanced homologous targeting ability compared to the control group. Intravenous administration of bionic nanoparticles resulted in higher tumor suppression with fewer side effects.^564^

The efficacy of many chemotherapy medications is reduced when used against tumors and metastases that are resistant to multiple drugs (MDR). Enhancing the dosage of medications yields minimal impact while enhancing the toxicity of the medication. Podophyllotoxin (PPT) is a cytotoxic drug that inhibits microtubule function, causing cell cycle arrest and programmed cell death. Additionally, it exhibits potent cytotoxicity against multidrug-resistant (MDR) cancer cells. Nevertheless, because of its limited solubility and lack of specificity, it is not suitable for systemic use. Roy et al. synthesized Celludo, a self-assembled PPT NP, through the covalent linkage of PPT and PEG to CMC-Ac, which was acetylated carboxymethyl cellulose, using ester bonds. Celludo exhibited an extended circulation time, with a half-life 18 times longer than that of free PPT. Additionally, it demonstrated a significant 9000-fold increase in accumulation under the curve (AUC) and a remarkable 1000-fold decrease in clearance compared to free PPT. The Celludo group exhibited a 500-fold increase in tumor delivery compared to the free PPT group. Celludo treatment in the animal model resulted in a significant increase in median survival without any observed toxicity.^565^ Additionally, in order to overcome the MDR in NSCLC, Cheng et al. developed a delivery system using polydopamine-coated mesoporous silica nanoparticles functionalized with TPGS and d-a-tocopheryl polyethylene glycol 1000 succinate (TPGS) for the controlled and pH-sensitive release of DOX. The existence of TPGS on the NPs’ surface caused a change in zeta potential from negative to positive, which in turn increased the uptake of tumor cells due to their negatively charged membranes. Furthermore, the TPGS alteration can provide NPs with the capability to uphold elevated levels of drugs within cells. MTT and in vitro cellular uptake experiments have demonstrated that NPs modified with TPGS can partially overcome MDR. The drug delivery system’s passive targeting capability can be improved by degrading the PH-sensitive PDA coating at low pH levels.^566^ The use of Cold atmospheric plasma (CAP), an innovative technique in the medical field, has the potential to enhance the potency of medications, thereby potentially mitigating drug resistance. Yu et al. reported a new strategy for treating cancer by combining CAP with innovative PTX-loaded NPs. They combined PTX with magnetic NPs and used biodegradable polylactic acid (PLA) as the NP shell. The results of the experiments indicated that the combination of PTX-loaded nanoparticles and CAP had a stronger inhibitory effect on the growth of A549 cells compared to the sole use of CAP. Therefore, the fusion of PTX-encapsulated nanoparticles and CAPs offers a hopeful instrument for the advancement of treatment approaches for NSCLC.^567^

Targeting mitochondria for antitumor purposes has garnered attention due to the pivotal regulatory role they play in cell apoptosis. Nevertheless, the delivery of drugs targeted at mitochondria encounters various obstacles, such as intricate and multi-step preparation, limited drug capacity, and the potential for systemic toxicity caused by the vector.^568^ In order to tackle these concerns, numerous NPs have been created. Song et al. constructed a mitochondria-targeted nanodrug by employing a straightforward nanoprecipitation technique with BDs, which are berberine derivatives substituted with 9-O-octadecyl. To enhance stability, the BDs-based nanodrugs were modified using DSPE-PEG2000, a derivative of bis-stearyl-phosphatidylethanolamine-methoxy-polyethylene glycol 2000. BDs-NDs underwent dual modification using PEG and hyaluronic acid, resulting in a surface charge of −25.8 mV and a significant capacity for drug loading. The degradation of HA by HAase in the tumor tissue leads to the exposure of positively charged PEG/BD NDs to cells. This exposure facilitates cellular uptake, escape from lysosomes, and targeting of mitochondria, ultimately leading to cell apoptosis. Mitochondria-targeted BD NDs provide an alternative approach for cancer therapy by improving drug loading and minimizing vector toxicity, in contrast to traditional NPs.^569^ Hydroxyapatite NPs (HAPNs) displayed fine biocompatibility and bioactivity, which are widely used in repairing hard tissue injuries. They are also used as carriers for drugs, proteins, and genes. The toxic effects of hydroxyapatite on different types of cancer cells have been documented. It has the ability to induce apoptosis through mitochondria-dependent pathways or accumulate in the endoplasmic reticulum, thereby inhibiting protein synthesis. Nevertheless, the precise cytotoxic mechanisms of HAP towards cancer cells remain inadequately comprehended, underscoring the significance of elucidating the specific interactions between HAP and cancer cells in the realm of nanomedicine. During their research, Sun et al. discovered that without any surface modification or bio-coupling targeting, HAP has the ability to successfully move into the mitochondria of cancer cells. This leads to a reduction in mitochondrial membrane potential and an increase in caspase-3 and caspase-9 enzyme activities, ultimately causing mitochondria-mediated apoptosis in A549 cells. The viability of 16HBE tracheal epithelial cells is not affected by HAP. Therefore, HAP has a promising future as a new safe and effective targeted anti-lung cancer drug.^570^

The multiphase Fenton reaction can continuously produce toxic hydroxyl radicals through the redox reaction of lattice iron ions with H_2_O_2_ in a high H_2_O_2_ levels environment, while normal physiological concentrations of catalase can protect healthy cells from this kind of attack. Compared to normal cells, cancer cells have high H_2_O_2_ level and low catalase levels. Therefore, the application of a heterogeneous Fenton reaction can selectively kill cancer cells. The recently synthesized spinel oxide SnFe_2_O_4_ as a magnetically recyclable multiphase Fenton catalyst showed excellent degradation efficiency, and Lee et al. successfully developed a specific H_2_O_2_ redox catalyst for SnFe_2_O_4_ nanocrystals. The findings clearly indicated that the SnFe_2_O_4_ nanocrystals treated with ultra-sonication exhibited a significant cytotoxic effect on tumors. The cancer cells can efficiently aggregate them through the use of either the EPR effect or the magnetically guided drug targeting technique. Meanwhile, SnFe_2_O_4_ nanocrystals can be slowly degraded in the lysosomes and eventually converted to metal ions for rapid excretion through the kidneys.^571^ Sun et al. developed cysteine-surface-modified FePt NPs. Extensive in vitro and in vivo investigations demonstrated that these nanoparticles effectively hindered tumor development by suppressing cell proliferation, enhancing sensitivity to radiotherapy, impeding cell migration and invasion, diminishing angiogenesis, and inducing the production of ROS. The release of FePt-Cys nanoparticles in lysosomes could potentially occur via the Fenton reaction. In vivo, the safety and effectiveness of FePt-Cys NPs have been shown in animal experiments. Nevertheless, further research is required to examine their pharmacokinetics and systemic toxicity.^572^

#### Gene therapy

Conventional therapies for lung cancer include surgical resection, chemotherapy, and radiotherapy. Owing to the deeper understanding of cancer development and progression at the gene level, gene therapy is expected to be an emerging and encouraging strategy for lung cancer. It involves specifically targeting and regulating abnormal genetic expressions via oligonucleotides such as small interfering RNA (siRNA), microRNA (miRNA), and long non-coding RNA (lncRNA).^573^ Nevertheless, on account of their negative charges, these oligonucleotides are lacking in the capacity to move across the cell membrane and enter the nucleus to exhibit an effective impact on cancer cells. Therefore, developing suitable and useful delivery systems for genes and molecules is necessary. With the development of nanotechnology, various NPs have been developed and widely used in gene delivery for lung cancer treatment. The delivery systems based on nanotechnology can protect the genetic fragments from degradation and effectively transport them into cancer cells.^574^ What’s more, through surface modification or conjugation with biomolecules of NPs, they can more precisely act on the aimed cells. Here we outline the research on NP delivery systems applied in gene therapy for lung cancer in recent years.

Recognized as a potent element in the treatment of lung cancer, siRNA is the primary driving force behind RNA interference (RNAi) technology. However, its poor cell absorption and high clearance limited its further application.^575^ Yan et al. developed a functional polyester capable of specifically delivering siRNA to lung cancer cells while sparing normal cells. The researchers created a collection of 840 functional polyplex NPs using a polymer-based library. These NPs were formed by combining diacyl chlorides with trimethylolpropane allyl ether, allowing for an adjustable molecular weight. By conducting high-throughput screening of libraries, it was discovered that polyesters possessing reduced molecular weight and hydrophobic side chains exhibited enhanced stability for siRNA delivery. Subsequently, the researchers introduced a small interfering RNA (siRNA) that specifically targeted ubiquitin B (UBB) into the NPs. They observed that these functional polyplex NPs effectively suppressed tumor growth by promoting siUBB-induced apoptosis while exhibiting no harm to healthy cells.^576^ Shi et al. consequently developed a nanoplatform with a poly(*L*-histidine) foundation that serves multiple functions. At pH 7.4, the combination of siRNA and the dendritic poly-*L*-lysine (PLL) was linked to the copolymer mPEG-PHis-PSD, which consists of methoxy poly (ethylene glycol), poly (*L*-histidine), and poly (sulfadimethoxine). The pH-sensitive delivery mechanism has the potential to enhance cellular uptake and facilitate the release of siRNA, evading the impact of the endo/lysosome. Furthermore, studies have demonstrated greater efficacy in suppressing tumors and a more favorable safety profile.^577^

The biological progression of lung tumor is greatly influenced by miRNA, particularly in relation to the tumor environment.^517,578^ Nowadays, more and more studies suggest that specifically miRNA may be involved in tumor management via monitoring and regulating tumor growth and resistance to the immune system.^579,580^ However, current studies on miRNA delivery are insufficient. P53, the tumor suppressor gene, is lacking or mutated in half of NSCLC cases. Mouse double minute 2 (MDM2) is an important modulator of P53. Morro et al. developed the CCL660 system encapsulated miR-660, which targets MDM2 in cationic Lipid-NPs.^581^ Systemic administration of CCL60 in mice models of lung cancer resulted in an upregulation of miRNA levels within tumors and an inhibition of tumor growth in both P53 wild-type and mutation-type tumors. This was achieved through the restoration of P53 and the suppression of MDM2 levels. It also exhibited no side effects on the surrounding normal tissues. Besides, it could also inhibit tumor metastasis. MiR-200c is another miRNA that plays a significant role in NSCLC. Its lower expressions are associated with a low grade of differentiation in tumors. It has been demonstrated to be capable of inhibiting cancer growth. Similar to the other gene therapies, miR-200c also needs a suitable delivery system. Peng et al. employed monomethoxy poly (ethylene glycol) as the hydrophilic backbone and ethyl-p-aminobenzoate (EAB) as the hydrophobic pendant groups to create an amphiphilic polyphosphazene polymersome with a neutral charge. This NP embedded miR-200c and protected it from being damaged. Due to the system’s electroneutrality, systemic toxicity could be avoided, and miR-200c can maintain a longer period of stability in circulation.^582^ Furthermore, Maryna et al. constructed a delivery system that delivers miRNA-29b into mucin-1(MUC1)-overexpressing cells. They combined IgG with poloxamer-188 to stabilize the system and conjugated MUC1 aptamer to the surface of the particles to achieve MUC1 targeting. This hybrid NP delivery system could limit the 100% release of miRNA-29b in an environment of pH = 5, so as to ensure that no miRNA enters the blood or tumor extracellular microenvironment and achieve effective release of miRNA to targeted positions and cells.^583^

Current studies for the restoration of p53 expression, including small molecules and DNA, have achieved progressive success but still have limitations. Interestingly, Kong et al. developed a NP platform for effective delivery of p53-encoding mRNA, which is redox-responsive and promotes a large amount of p53 expression. By inducing cell cycle arrest and apoptosis, the researchers exhibited a notable suppression of lung tumor cell growth. Additionally, it was discovered that the reintroduction of p53 could greatly enhance the responsiveness of cancer cells to everolimus, a medicine that inhibits mTOR.^584^

Lung cancer has been extensively demonstrated to be influenced by lncRNAs, which serve as significant and innovative regulators. One lncRNA, metastasis-associated lung adenocarcinoma transcript 1 (MALAT1), has been considered critical in lung tumor spread.^585^ More lncRNAs related to lung cancer are being established and defined. However, unlike siRNAs and miRNAs, lncRNA is rarely employed to treat lung cancer based on NPs. Despite this, they possess immense possibilities in the realm of lung cancer therapy. For instance, recently, Teng et al. constructed a delivery complex via coating Au NPs with antisense oligonucleotides (ASOs) and a nucleus-targeting TAT peptide. Au NPs have excellent biocompatibility and enzyme resistance. The TAT peptide helps the complex enter the nucleus through the nuclear pore complex. Then ASO recognizes and binds to MALAT1 to form a DNA-RNA heteroduplex. It will be degraded by RNase to achieve downregulation of gene expression.^586^

#### Immunotherapy

**Fig. 3:**
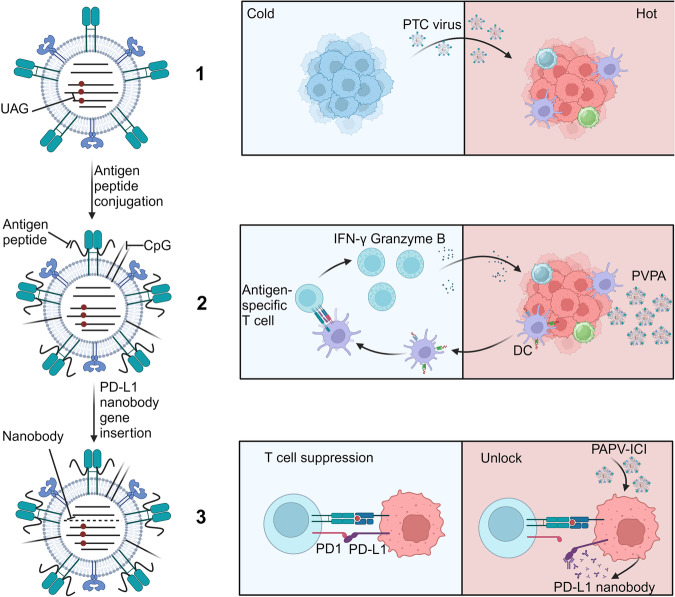
Schematic diagram of the PAPV-ICI platform for personalized cancer immunotherapy using peptide-armed PTC virus for lung cancer vaccination. PTC virus infection causes a transient proinflammatory state turning ‘cold’ TEM to hot, PAPV endows high DC Recruitment linked with generation and trafficking of antigen-specific CTLs; PAPV-ICI in situ delivers anti-PD-L1 nanobody synergizing anti-cancer immunotherapy. PAPV: peptide-armed PTC virus, PTC virus: the live but non-productive influenza A virus, ICI: immune checkpoint inhibitor. (Created with BioRender.com) Reproduced with permission from Ji et al. (2023), Copyright 2023 Springer Nature America, Inc

The progress of cancer vaccines, which activate the immune system’s anti-cancer reactions, has encountered challenges primarily because of the complexities of efficiently administering tumor neoantigens. Ji et al. introduced a new strategy that involved the use of a hybrid antigenic peptide influenza virus (CAP-Flu) platform. The main purpose of this system is to efficiently transport influenza A virus (IAV)-coupled antigenic peptides exclusively to the lung. The scientists combined weakened influenza A viruses (IAVs) with the natural immune-boosting substance CpG. Afterwards, they used to click chemistry to covalently display the model antigen ovalbumin (OVA) on IAV-CPG. The use of this structure via immunization led to a significant uptake of antigens by dendritic cells, a focused immune cell reaction, and a remarkable increase in the presence of lymphocytes infiltrating the tumor, in contrast to the use of peptides alone. Furthermore, the IAV was specifically engineered to produce nanobodies that target PD1-L1, leading to an enhanced reduction in lung metastases and an extended survival time in mice (Fig. 3).^587^

CpG oligodeoxynucleotides are among the most potent activators of toll-like receptor 9 (TLR9). Preclinical studies and clinical trials have demonstrated promising results. The combination of CpG and TLR9 can trigger both innate and adaptive immune responses. Initially, it stimulates the dendritic cells, resulting in a cascade of subsequent effects, such as the release of proinflammatory cytokines, the activation of natural killer cells, and the proliferation of T cell populations. Perry et al. used PRINT (particle replication in nonwetting templates) NPs as vectors for CpG, which could deliver CpG into the lungs of mice through intratracheal instillation. The results indicated that this system could effectively promote the substantial regression of tumors and induce the developed immune memory of treated mice. Moreover, the employing of this NP-based system prolonged the retention time of CpG in the lungs as well as promoted the levels and duration of antitumor cytokines. Therefore, PRINT-CpG is an efficacious nanotechnological platform applied in local treatments for lung cancers and may also have the capacity to potentially treat lung metastases of various kinds of malignancies.^588^

#### Radiotherapy

The use of radiation therapy is an essential component in the management of lung cancer. To date, 60% of NSCLC patients require radiotherapy. Nevertheless, conventional radiotherapy still presents obstacles like significant adverse reactions and a limited rate of local control due to the insufficient radiation absorption by cancer tissues and the administration of high levels of ionizing radiation, ultimately impacting the patients’ prognosis. Therefore, it is important to avoid leftover cancer cells, which could produce resistance to radiotherapy. It is reported that tumor growth and aggressiveness were distinctly decreased through low-dose radiation (LDR) pretreatment followed by radiotherapy. Moreover, the molecular mechanism has been studied in that miR-30a and miR-30b were overexpressed after LDR treatment in NSCLC, which could downregulate the expression of plasminogen activator inhibitor-1 (PAI-1).^589^ The advancement of nanotechnology has significantly broadened the use of radiotherapy for lung cancer.^590,591^

To strengthen the curation effects, sensitizers are widely used during radiotherapy. Sun et al. have successfully created cysteine-modified FePt-Cys NPs that are safe and effective. These NPs exhibit an enhanced radiotherapy effect by activating the caspase cascade reaction and also inhibit DNA damage repair in lung cancer. Thus, the FePt-Cys NPs have the potential to act as a sensitizer for chemo-radiotherapy.^572^ Reda et al. developed a novel PLK1 and EGFR-targeted nanomaterial, which emerged as a finely targeted therapy and enhanced radiation sensitivity in NSCLC.^592^ In another study, Sanche et al. indicated that Au nanomaterials have the potential to act as proficient sensitizers in cisplatin when exposed to X-ray radiation. This resulted in a three-fold increase in breaks in the DNA double strand when one cisplatin molecule was combined with one Au NP and a 7.5-fold increase when two cisplatin molecules were combined with one Au NP.^593^ Silver nanomaterials have also been studied to improve the radio-sensitivity activity, which could not only induce X-ray absorbance in cancer cells but also enhance electron trapping and ROS generation due to the production of Ag^+^.^590,594,595^ In non-small lung tumor cells, it has been studied that chitosan-coated Ag triangular nanomaterials enhanced the radio-sensitization effect.^596^ A recent study reported that DM1-NO-coated PLGA-b-PEG NPs could selectively sensitize H1299 cells to radiation therapy.^597^

Compared with normal tissues, the tumor microenvironment is hypoxic, which leads to more radiotherapy tolerance in tumor tissues.^598^ It has been reported a kind of synthetic NPs, perfluorocarbon (PFC), composed of fluorine and carbon atoms, has been used due to its high oxygen carrying property, satisfactory biocompatibility, and nontoxic ability. The work first emerged oxygen in the lung by PFC nano-droplets, and then a low-power ultrasound transducer could help oxygen release in the tumor, resulting in remarkable tumor oxygenation and improved treatment effects.^599^ Meanwhile, the PFC nanomaterials also demonstrated satisfactory biocompatibility and strong tumor penetration for better MR imaging upon being administered intravenously into the lung cancer rabbit model.^537^

Furthermore, the standard treatment for lung cancer often involves the utilization of both chemotherapy and radiotherapy.^600–602^ Administering chemotherapeutic medications systemically can enhance the effectiveness of radioisotope tracers on primary cancers and also suppress the growth of distant metastatic malignancies. However, this combination may induce higher toxicities than a single treatment. Nanomaterials show prominent advantages, such as targeting properties and controlled drug release. Ma et al. have designed Bi_2_S_3_ NPs composed of mesoporous silica (BMSNs), which exhibited a strong carrying DOX capability and stable drug release kinetics, as well as improved antitumor effects against multidrug resistance compared with that of free DOX. Meanwhile, a better therapeutic effect was observed when X-ray radiotherapy was added to nanotechnology chemotherapy.^603^ Thus, the BMSNs may be a promising nano-system agent to enhance radiotherapy for clinic applications.

#### Photothermal therapy

In recent decades, photothermal therapy (PTT) has made great progress, and cancer treatment has also been rapidly developed. As for the patients with local NSCLC who have not yet undergone a surgical operation, PTT is a selectable therapy method.^604^ This method mainly depends on PTT agents, which are able to absorb light and then dissipate heat energy locally, causing irreversible cell damage.^604^ However, it still faces challenges due to the low efficiency of photothermal conversion by NIR-II materials. In a recent study, researchers used organically conjugated small molecules to remold PTT agents and found that they could reach the NIR-II region by only replacing one type of atom. In vivo and in vitro experiments have demonstrated that this reconstructive agent has efficient tumor treatment capabilities with a 77% photothermal conversion efficiency. IR-SS NPs also showed significant killing effects from the photothermal therapy in the A549 cells under NIR-II irradiation.^605^ In another study, innovative GNPs were assembled for hPD-L1 siRNA delivery, forming GNPs-hPD-L1 siRNA as a hyperthermia agent for PTT. The HCC827 cell growth was significantly depressed, and the hPD-L1 expression was down-regulated with the combination of GNPs-hPD-L1 siRNA and PTT. Innovation in an alliance with GNPs and hPD-L1 siRNA may be an effective strategy in clinical applications.^606^

In the clinic, gefitinib is a targeted drug to cure advanced NSCLC. However, resistance to gefitinib can be mainly induced by the activation of bypass signaling, including insulin growth factor-1 receptor signaling (IGF1R). It has been reported that CuS NPs could enhance the ROS level in tumor cells by near-infrared laser irradiation, thus impeded bypass IGF1R and its downstream AKT/ERK/NF-κB signaling cascades to solve the resistance of gefitinib induced by IGF1R bypass. This can provide a new strategy to cure drug-resistant tumors.^607^

For early-stage peripheral lung tumors, research has adopted a minimally invasive method, which showed superiority in that the synthesized porphysome NP could accumulate in lung tumors. The ability to selectively accumulate in tumor segments greatly enhances the efficacy and safety of PTT. Meanwhile, the fluorescence-directed bronchoscope could deliver real-time imaging of lung tumors for transbronchial PTT. In the clinic, the use of nanomaterials in the newly developed technology could potentially offer a secure and efficient treatment option for peripheral lung cancer in its early stages.^608^

#### Combined therapy

Furthermore, there are some other studies that also indicate that combined therapy of siRNA and chemotherapeutic drugs carried on NPs has enormous potential in clinical treatment for lung cancer.^609–612^ For lung cancer, particularly advanced NSCLC, monotherapy based on chemotherapy or other strategies lacks enough efficacy. As mentioned earlier, cisplatin often induces adverse effects and drug resistance. Incorporating siRNA can enable the use of a lower drug dosage, mitigate the side effects, and augment the medical effectiveness of chemotherapeutic treatments.

Mitotic arrest deficient-2 (Mad2), an essential mitotic checkpoint gene, is significantly related to the proper segregation of chromosomes during mitosis. So Mad2 silencing by siRNA is an attractive gene therapy. Nascimento was encapsulated with siRNA targeting Mad2 into PEGylated chitosan NPs and then mixed with EGFR-targeted CS to construct a targeted NP system. The findings indicated that reducing the expression of Mad2 through siRNA could overcome drug resistance and enhance the responsiveness of lung cancer cells to cisplatin while causing minimal toxicity.^613^ Mutation of the Kirsten ras sarcoma viral oncogene homolog (KRAS) is also commonly seen in NSCLC. Besides, the loss of p53 function can accelerate KRAS-derived tumorigenesis. Hence, the restoration of p53 and the suppression of the KRAS oncogene are feasible treatment strategies. Gu et al. embedded cisplatin in liposomes and then constructed them with multiple polyelectrolyte layers containing siR-KRAS and miR-34a. This multifunctional layer-by-layer NP displayed a preferential uptake in lung tumors and improved the effect of cisplatin, due to the fact that siR-KRAS could knock down the KRAS oncogene and miR-34a stimulated the p53 functional pathway. Moreover, the duration of treatment was extended owing to the control of drug release by the NP.^614^ Tumor progression involves the promotion of angiogenesis by VEGF, which is abundantly present in numerous malignant tumors. Anti-angiogenic agents such as siR-VEGF and etoposide contributed to tumors inhibition. However, the efficacy of anti-angiogenic monotherapy is unsatisfactory and insufficient; VEGF siRNA suffers from enzymatic degradation, while etoposide has poor solubility. To solve this problem, Li et al. designed a multi-functional NP composed of cationic liposomes to co-deliver VEGF siRNA and etoposide, targeting orthotopic NSCLC. These NPs can avoid drug leakage and assure sufficient concentration, while this combined therapy has been proven to have significant anti-tumor effects in vitro experiments.^615^

The most aggressive types of NSCLC are those that have mutated EML4-ALK gene expression. So, the mutation of EML4-ALK has become a unique target in NSCLC. SiRNA is mostly expected to be applied to suppress ALK mRNA. Nevertheless, solely suppressing ALK mRNA is not sufficient; thus, miR-301a, also relating to the progression of lung cancers, is considered a new target. Therefore, Li et al. prepared a gold nanoshell-based system carrying ALK siRNA and miRNA-301 inhibitors, which was also coated with DOX via electrostatic adsorption. This delivery system significantly increased vascular permeability and therefore promoted drug accumulation in tumors, depending on its photothermal effect and dense spherical structure. Most importantly, it indicated that this method, based on gold nanoshells, has the potential to serve as a highly effective platform for the combination targeted therapy of siRNA, microRNA, and drugs.^616^

Genistein, naturally occurring in soybeans, has been proven to elevate the anticancer effects of some chemotherapeutic drugs. MiRNA-29b can suppress the antiapoptotic defense or stimulate the apoptosis of tumor cells. To assess the therapeutic outcomes of the combined treatment of genistein and miRNA-29b for NSCLC, Sacko et al. constructed genistein-miRNA-29b-loaded functionalized hybrid NPs (GMLHN) with MUC 1-aptamer. They found that GMLHN functioned better in proliferation suppression of tumor tissues than monotherapy with genistein or miRNA-29b, with lower possible toxicity. Because genistein and miRNA act on different targets by various mechanisms, their combination based on NPs can effectively downregulate oncoproteins such as phosphorylated protein kinase, myeloid cell leukemia sequence 1, phosphorylated phosphoinositide 3-kinase, etc.^617^

Apart from the combined therapies above, Garbuzenko et al. put forward a novel multi-tier biotechnology treatment approach because current non-surgical treatment strategies for NSCLC bring about tremendous disadvantages. Chemotherapy will result in many side effects and drug resistance. Using monoclonal antibodies, or TKIs, to block targets like EGFR is limited to a small portion of patients. They encapsulated PYX and a pool of siRNAs that specifically target the four main EGFR-TKIs together into nanostructured lipid carriers (NLC) with luteinizing hormone-releasing hormone (LHRH) decapeptide. The LHRH decapeptide helped the loaded drug and siRNA reach the cancer cells of the lung and enhanced the accumulation of the NP system in lung tumors after inhalation delivery. They also found that the co-delivery system had a significantly higher efficacy in tumor growth inhibition than monotherapy.^618^

The use of photosensitizer and laser irradiation to generate ROS makes photodynamic therapy (PDT) an innovative method for treating tumor diseases. For a better treatment, it is necessary to combine PDT and ROS-responsive chemotherapy.^619,620^ Yue et al. developed NP Ce6-CPT-UCNPs with fine biocompatibility. The researchers have verified that the nanoparticle is capable of generating reactive oxygen species (ROS) when exposed to 980 nm laser radiation and releasing the anticancer medication CPT. The Ce6-CPT-UCNPs have effectively accumulated in lung tissues and inhibited the growth of cancer in both in situ lung tumor-bearing mice models and subcutaneous lung cancer models following intratumoral injection of Ce6-CPT-UCNPs and 980 nm laser irradiation. Tumor recurrence and metastasis have not emerged even after fifty days.^620^ Xia et al. designed gold nanocluster-based Ce6-DOX-GNCs-MMP2 polypeptide NPs (CDGM NPs) for imaging and the combined treatment. They made a complex to deliver the photosensitizer chlorin Ce6, the chemical drug DOX, and the targeting effects of the MMP2 polypeptide into A549 cells. This particle exhibited a fine combination effect, which may be a potentially effective nanoprobe for lung cancer treatments.^621^ Zhong et al. have reported a novel and creative NaCeF_4_: Gd, Tb scintillator. In this field, cerium (Ce) was responsible for ROS generation due to photocatalytic activities and also acted as a sensitizer to help Tb ions release fluorescence by X-ray irradiation. Furthermore, both the Ce and Tb ions could assimilate the energy of secondary electrons under X-rays to generate ROS for radio dynamic therapy. Through the absorption of X-rays, the nanomaterials could help with CT imaging and radiotherapy sensitization. More than that, due to the heavy atoms of Ce, Gd, and Tb ions, the local radiation dose deposition was also significantly enhanced in vitro. The anticancer efficiency of combination therapy was higher than that of RT alone in the A549 mice model. Their work mainly adopted ScNPs to guide multimodal imaging into deep tumors, which is considerable for further study and clinical applications.^622^ In the situation of recurrence and metastasis of lung cancer, it may be a considerable approach to combine immunotherapy and photodynamic therapy. Song et al. have creatively designed PplX-1MT nanomaterials, which could include the photosensitizer PpIX and the immune checkpoint inhibitor 1MT. When stimulated by 630 nm light, the nanomaterials induced the apoptosis of cancer cells by generating ROS and strengthened the immune system by releasing 1MT. They were able to promote the activation of CD8^+^ T cells, showing a better treatment effect.^623^

To overcome the weaknesses of high toxicity, poor imaging effect, and ineffective curation of some nanomaterials, it has been reported that a novel BiOI@CuS NP co-coated with DOX, aspirin, phenacetin, and caffeine. This NP showed a safe and effective therapy effect in lung cancer, and it can also alleviate the inflammatory response caused by PTT. In this study, this NP possessed chemo-photothermal treatment function and favorable CT imaging features. Consequently, BiOI@CuS NP may be a potential imaging and treatment technology for lung cancer.^624^

For early lung cancer, the main detection method is spiral CT.^625^ In the curation of lung cancer, near-infrared fluorescence (NIRF) imaging has been instrumental in better diagnosis and treatment, which could provide high-contrast pictures.^626,627^ The NIRF molecules can also be used as a contrast agent in surgery due to their clear distinction between tumor regions and normal segments in real time.^628^ However, the NIRF molecules usually stay in bloodstream for a short time. On et al. synthesized glycol chitosan nanoparticles (CNPs) modified with a fluorophore. These NPs were able to effectively aggregate in the tumor region. The NIRF signals emitted by the nanomaterials were utilized to accurately locate the tumor in mouse and rabbit VX2 lung cancer models. Meanwhile, CNPs were utilized as an imaging contrast agent for performing surgery under image guidance. Hence, CNPs can be used in the diagnosis and treatment of tumors.^629^

## Nanomedicine in glioblastoma

Glioblastoma is a broad classification for tumors that develop from glial cells and neuronal cells within the nervous system. It is the prevailing type of brain tumor, known for its aggressive nature and high fatality rate. In the United States, glioma has an estimated annual occurrence of around 6 instances per 100,000 individuals. Glioblastoma represents roughly 60% of these cases, with males being predominantly affected.^630^ The 2016 World Health Organization (WHO) reclassified glioblastoma into: (1) Gioblastoma, IDH-wildtype (about 90% of cases), which commonly associates with primary or de novo glioblastoma; (2) Glioblastoma, IDH mutation type (about 10% of cases), is similar to so-called secondary glioblastoma, which has a history of low-grade diffuse glioma, especially in young patients; (3) Glioblastoma, NOS, cannot be evaluated with full IDH and is classified as such.^631^ What’s more, in addition to primary tumors, cancer patients with lung cancer, breast cancer, and melanoma often have brain metastasis or secondary brain tumors.^632,633^

### Diagnosis

It is well known that glioblastoma is highly aggressive and easily penetrates normal brain tissue around tumor cells. Surgical resection is usually the treatment standard for glioblastoma, but physical methods such as surgery often lead to poor therapeutic effects and a high recurrence rate of GBM after surgery because the tumor boundary far exceeds the annular enhancement of T1 contrast MRI radiographic imaging and the whole tumor cannot be completely resected.^634^ At present, imaging diagnostic technologies commonly used in treating glioblastoma mainly include CT, PET, MRI, etc., but these imaging technologies have their drawbacks. Hence, it is imperative to create novel imaging techniques or contrast substances to ensure glioblastoma patients can obtain a precise diagnosis, which serves as the first vital phase in effectively treating glioblastoma.^635^ With the rise of nanotechnology, imaging diagnostic technology has developed rapidly. In many clinical trials, some nanocarrier systems, such as liposomes, micelles, or polymer NPs, have been developed to deliver imaging or therapeutic agents into the brain.^636,637^

Aiming at related clinical problems of tumor imaging, Hou et al.^638^ utilized a pre-targeting strategy to develop PET imaging technology. To begin with, the integration of supramolecular NPs and bioorthogonal chemistry is employed for the synthesis of tumor-targeting agents known as TCO⊂SNPs. Following the administration of TCO⊂SNPs through intravenous injection, the tumor’s EPR phenomenon facilitates the selective gathering of TCO⊂SNPs within the tumor. As TCO⊂SNPs build up in the tumor, they break down to release TCO/CD-PEI, which is a molecular building block grafted with TCO. Subsequently, a radiolabeled reporter molecule (64Cu-TZ) is administered while TCO/CD-PEI remains present in the tumor. Following that, the bio-orthogonal reaction takes place, simultaneously ensuring the swift removal of any remaining 64Cu-TZ from the body. High-contrast PET imaging of the tumor is significantly enhanced due to the limitation of radioactivity in the tumor by the conjugation adduct (64Cu-DHP/Cd-PEI), resulting in improved imaging quality. Using bimodal and fluorescently labeled iron oxide NPs, Karimian-Jazi et al.^639^ successfully established a multimodal imaging technology platform using MR-MPM with iron oxide NPs as the core as the innate immune cell response in the microenvironment of glioma tumors, using related MRI and multi-photon microscopy (MR-MPM) with mouse glioma as the model. The imaging technology allows for the dynamic and static visualization of tumor-associated macrophages and microglia (TAMs) within the TME. It also enables the tracking of innate immune cells within the same animal, allowing for longitudinal studies on the dynamics of these cells in the TME. This provides a valuable reference for future research on glioblastoma.

Near-infrared fluorescence (NIRF) imaging, typically conducted at an excitation wavelength of 700–800 nm, can enhance the precision of glioblastoma boundary identification during surgery. This enables neurosurgeons to remove tumors with heightened sensitivity and accuracy.^640^ However, it is difficult to locate the glioblastoma boundary accurately, especially its infiltration area, which hinders the application of this technique. Recently, Reichel et al.^641^ conjugated ferumoxytol (FMX), a superparamagnetic iron oxide NP sensitive to MRI approved by the FDA, with heptamethine cyanine (HMC), and designed an HMC-FMX fluorescent nanoparticle probe based on near-infrared fluorescence (NIRF), which can be used for visualizing tumor boundaries and image-guided drug delivery to glioblastoma. The NPs can pass through the BBB and selectively accumulate in tumors to visualize invasive tumor tissues. Furthermore, HMC-FMX has the capability to enclose anti-cancer medications like paclitaxel or cisplatin and transport them to glioblastoma tumors.

However, before applying NPs imaging to clinical practice, it is necessary to further study the biological distribution and clearance of NPs, especially in nerve applications with damaged blood-brain barriers, and also consider the safety of NPs contrast agent residues in the brain.^642^

### Treatment

Although surgical resection is still the preferred treatment for glioblastoma, its prognosis is poor, and glioblastoma recurs easily. Adjuvant chemotherapy used after surgery normally has unsatisfactory efficacy due to the presence of BBB. The development of nanotechnology has promoted the delivery of drugs to glioma cells by different mechanisms, such as the EPR effect, P-glycoprotein inhibition, CMT-mediated, and EMT-mediated active targeting.^643^ Simultaneously, nano-drugs have been effectively utilized in research involving microenvironment, targeted therapy, gene therapy, and immunotherapy through active targeting.

#### Chemotherapy

Cytotoxic drugs are usually used to kill tumor cells in chemotherapy, but due to their lack of targeting, they often have a toxic effect on normal cells and have great toxic and side effects. In addition, the presence of BBB makes it difficult for drugs to act on glioblastoma through BBB. The development of nanotechnology has contributed greatly to treating complex diseases and overcoming the challenges of various drugs reaching the brain. Utilizing NPs as a means of delivering medication can achieve focused treatment, shield drugs from metabolic deterioration, enhance the localized drug concentration, and mitigate the harmful and adverse impacts of drugs on healthy cells.^644,645^ Nano-drugs can be passively targeted for the accumulation and diffusion of brain tumors using the EPR effect. However, due to the lack of specific targeting, the therapeutic effect is sometimes poor.^646^ Another method is called active targeting. With BBB, or overexpressed vectors or receptors in glioblastoma, as the target, it can be targeted to the tumor through BBB mediated by CMT or RMT. On the one hand, it can improve the accumulation of drugs and their efficacy. On the other hand, toxic effects and side effects can be reduced. Among these, the receptors that have been extensively researched for brain targeting are transferrin receptors, low-density lipoprotein (LDL) receptors, and insulin receptors.^647–649^

Transferrin receptor 1 (TfR1), which is highly expressed in BBB and glioblastoma cells, can mediate the transport of iron into the brain through the binding and intracellular transport of transferrin (Tf). A research group has found that heavy-chain ferritin (HFn) can specifically bind to TfR1 and use HFn as nanocarriers to load drugs and specifically deliver them to targeted tissues through TfR1-mediated targeting without any ligand modification.^650–652^ At the same time, they found that HFn targeted at glioma cells almost accumulates in lysosomes, causing the glioma cells to be killed without affecting the surrounding healthy tissues.^653^ On the other hand, studies have found that the receptors of Apoferritin (APO) and lactoferrin (LF) are overexpressed in various cells, so they can also be designed as NPs to specifically target brain tumors.^654,655^ In addition, low-density lipoprotein receptor (LDLR)-mediated targeted transport has also attracted extensive attention. Researchers functionalized novel polyester-based nanoparticles with a peptide with a special affinity for LDLR (the regulator EGR) and prepared paclitaxel-loaded nanoparticles. The results also showed that the functionalized nanoparticles increased transport in BBB and enhanced antiproliferative efficiency.^656^

Glioblastoma treatment commonly involves the use of Temozolomide (TMZ), which is the first-line chemotherapy medication. TMZ is used during radiotherapy (75 mg/m^2^ every day), followed by 6 courses of TMZ (150–200 mg/m^2^, once every 28 days on the first to fifth days). However, long-term use of TMZ therapy will lead to drug resistance in patients, and direct use of chemotherapy drugs will cause serious systemic problems. To solve these problems, researchers are committed to transforming nanoparticles (for example, iron oxide NPs, gold NPs, nanoliposomes, etc.) as a drug delivery system to deliver chemotherapy drugs into the body. The aforementioned NPs have the ability to cross the BBB and transport medicinal substances to the specific location of glioblastoma tumors.^657^ In addition, it is reported that the NPs are loaded with DOX, camptothecin, paclitaxel, curcumin, indomethacin, and other drugs, which can not only prolong the drug release time but also significantly improve the drug release selectivity.^658^ Norouzi et al.^659^ employed DOX-loaded biocompatible magnetic iron oxide NPs (IONPs) that were stabilized with trimethoxysilylpropyl-ethylenediamine triacetic acid (EDT) as the carrier for glioblastoma chemotherapy. The DOX-loaded EDT-ions have the capability to release DOX in a span of 4 days, and the acidic microenvironment can enhance its ability to release adriamycin. According to the study, DOX-EDT-IONPs have been found to enhance the absorption of DOX by U251 cells by 2.8-fold and effectively suppress the growth of U251 cells. In addition, it can effectively induce the apoptosis of glioblastoma cells.

Recently, there has been an increasing focus on the use of virus-like NPs to advance novel drug delivery methods. Certain nanocarriers inspired by viruses have demonstrated effectiveness in delivering drugs and genes.^660–662^ The research team created and assessed the nano-carrier MILB@LR, which is inspired by the remarkable capability of the rabies virus (RABV) to penetrate the central nervous system. This nano-carrier is built on the metal-organic framework called Materials Institut Lavoisier (MIL) metal-organic framework (MOFs), utilizing an iron atom as the foundation. By manipulating the water quantity during the synthesis of MIL, the morphology of MIL was modified to produce MILB. Subsequently, MiLB was further functionalized to obtain MILB@LR, which was then loaded with oxaliplatin. The vector closely resembles the elastic framework and surface functionality of the natural RABV. The findings indicate that MILB@LR loaded with OXA benefits from a more extensive RABV simulation approach, exhibits superior capacity to suppress tumor growth, has the potential to be a targeted and therapeutic drug for glioblastoma, and serves as a foundation for a bionic strategy to overcome biological barriers in vivo. This could ultimately enhance the diagnosis and treatment of central nervous system diseases.^662^ Despite numerous studies demonstrating the efficient transportation of drugs to tumor sites by virus vectors, the use of virus particles for drug delivery still poses inherent safety concerns, necessitating further research.^663^

#### Gene therapy

RNA interference (RNAi) technology has the characteristics of high specificity and low toxicity. The siRNA is introduced into cells to inhibit gene expression with high sequence specificity. Therefore, siRNA interference is a new and promising treatment method. By knocking out the gene expression that promotes cancer cell survival, migration, and tumorigenicity, siRNA can become a powerful tool to treat glioblastoma.^664–666^ Because gene drugs are easy to degrade in vivo and difficult to pass through the cell membrane, it is challenging to deliver genes safely and effectively into cells. Viruses are often used as nucleic acid delivery vectors, but their immunogenicity and toxicity hinder their application. Numerous advances have been made in the development of nanotechnology in the gene delivery system. A safer, more effective, and easier-to-produce siRNA delivery system has been developed by using nanotechnology to design and synthesize vectors carrying related genes, such as liposomes,^667^ silica nanoparticles,^668^ and dendritic polymers.^669,670^

There have been reports about encapsulating siRNA into nanocarriers for gene therapy for glioblastoma. Glioblastoma was targeted by a self-assembled gene delivery system created by a research team, which consisted of 1,2-dioleoyl-3-trimethylammonium-propane and methoxy poly (ethylene glycol)-poly (lactide) copolymer (DMP), enabling the delivery of Gli 1 siRNA.^671^ The researchers noted that the pairing of DMP and Gli 1-siRNA resulted in a notable decrease in Gli-1 protein expression, effectively impeding cell growth and enhancing apoptosis in glioma cells. Van Woensel et al. developed chitosan NPs containing siRNA (siGal-1) that specifically targets Gal-1 in the TME to achieve gene silencing. According to the research, the elimination of the Gal-1 gene resulted in a decrease in the transition of macrophages from the M1 (pro-inflammatory) to the M2 (anti-inflammatory) state during the advancement of glioblastoma.^672^

Researchers have recently developed a nano-drug called Ang-3I-NM@siRNA, which is sensitive to ROS and stabilized through triple interactions involving electrostatic forces, hydrogen bonding, and hydrophobic interactions. The addition of extra hydrogen and hydrophobic interaction has greatly enhanced the physiological stability, in contrast to siRNA nano-drugs that solely depend on electrostatic interaction for stability. Moreover, when Ang-3I-NM@siRNA comes into contact with cancer cells that are abundant in reactive oxygen species (ROS concentration in cancer cells reaching up to 100 µM^673^), the hydrophobic phenylboronic ester undergoes a conversion into its hydrophilic form with carboxyl groups. This transition from hydrophobic to hydrophilic depletes the hydrophobic stability, and at the same time, the newly formed carboxyl groups disrupt the interaction between static electricity and hydrogen bonds. As a result, siRNA can be efficiently released.^674^ The novel siRNA nano-drug exhibits threefold interaction stability and incorporates a self-destruction delivery capability, thereby offering a robust and efficient platform for targeted siRNA therapy of GBM. Moreover, it holds potential practical significance in RNA interference therapy for various tumors or brain disorders.^675^

#### Immunotherapy

In the field of cancer treatment, immunotherapy is rapidly advancing. In addition to chemotherapy, immunotherapy presents itself as a compelling alternative approach for addressing the tumor microenvironment in glioblastoma.^676–678^ Nevertheless, the limited success of immunomodulation in glioblastoma is attributed to the distinctive immune conditions of the central nervous system, including the BBB and blood-brain-tumor barrier, as well as the heterogeneous immunosuppressive microenvironment of glioblastoma.^679^ The outcomes of glioma immunotherapy vaccine clinical trials, oncolytic virus clinical trials, phase III clinical trials of checkpoint inhibitor antibodies, and chimeric antigen receptor-modified T cells (CAR-T cells) expressing chimeric antigen receptors were summarized in a recent review. However, the findings proved to be unsatisfactory.^673^ Up to now, no treatment method is superior to the standard therapy for glioblastoma represented by TMZ and radiotherapy.^674,680^

**Fig. 4:**
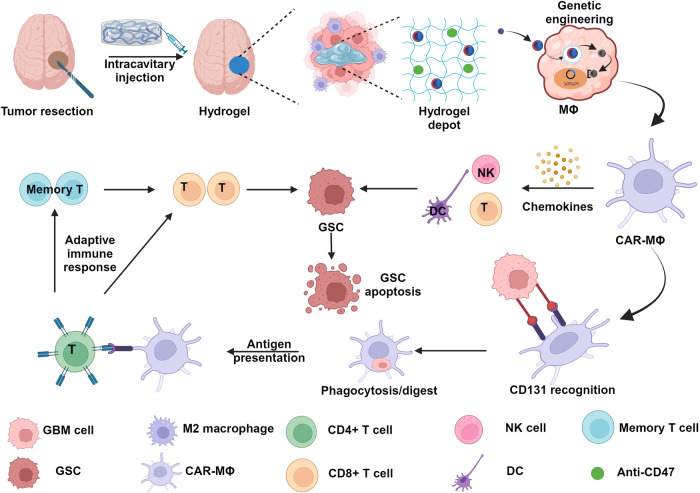
Intracavity generation of glioma stem cell-specific CAR macrophages for preventing postoperative glioblastoma relapse. (Created with BioRender.com) Reproduced with permission from Chen et al.^681^, Copyright 2022 American Association for the Advancement of Science

Glioblastoma multiforme poses significant challenges in terms of therapy due to its high recurrence rate. It is widely believed that the presence of glioma stem cells (GSCs) is linked to the occurrence of postoperative relapse. Chen and colleagues presented their creation of a nanoporter-hydrogel superstructure that can be injected into brain cavities. This superstructure is capable of generating chimeric antigen receptor (CAR) macrophages and microglia (MΦs) that are specific to CD133 as well as GSCs. These CAR MΦs surround the cavity, subsequently serving as a preventive measure against glioblastoma relapse. Additionally, the combination of anti-CD47 further increased the tumor suppression impact (Fig. 4).^681^

With the development of nanotechnology and deepening understanding of immunotherapy, nanotechnology can be combined with immunotherapy to deliver nano-sized immunoconjugates to glioblastoma, which is expected to achieve successful immunotherapy.^682^ Galstan et al. prepared targeted nanoconjugates using poly (β-*L*-malic acid) (PMLA) covalently bound to CTLA-4 and PD-1 antibodies (aCTLA-4 and a-PD-1) to deliver them to brain tumor cells via the transferrin receptor (TfR)-mediated exocytosis, and they observed an obvious increase in NK cells, CD8^+^ T cells, and macrophages in mice with intracranial GL261 glioblastoma, and the combination treatment with a checkpoint inhibitor also prolonged the animal survival compared to the use of a single checkpoint inhibitor.^683^

Schizophyllan (SPG), a soluble β-1,3-glucan extracted from *Schizophyllan commune*, is recognized as a dectin-1 ligand capable of effectively delivering brief DNA sequences into immune cells like macrophages and dendritic cells.^684^ Further, recent research has indicated that SPG NPs can function as a dectin-1 ligand that is not an agonist, facilitating the transfer of the payload to TLR9 within the body.^685^ Thus, Tiwari et al. evaluated the effects of C6 rat glioblastoma cells by using the fungal polymer SPG to load CpG ODN 1826 into prepared NPs. The NPs possessed a threshold size of 25.49 nm and had the capability to traverse the blood-brain barrier. This resulted in a notable rise in intracellular ROS and the expression of IFN-γ, along with IL-1β. The findings indicate that by stimulating significant levels of oxidative burst and inflammatory cytokines, M2 macrophages are prone to transitioning into the desired M1 phenotype, presenting a promising delivery mechanism anticipated to become a novel therapeutic approach for GBM.^686^ Therefore, the emergence of nanotechnology will promote the application of immunotherapy in glioblastoma treatment.

#### Combined therapy

Currently, there is a focus on developing approaches that can integrate chemotherapy and immunotherapy in order to amplify their effectiveness against tumors. Improved survival may be achieved by combining local chemotherapy and immunotherapy, which can reassemble tumor-infiltrating immune cells and memory T cells within the glioblastoma tumor microenvironment, thereby protecting animals from tumor re-attack. Preventing tumor recurrence relies on the crucial role of memory T cells in enhancing immune memory through immunotherapy. Xie et al. developed a nanocarrier that could deliver doxorubicin to glioma using a size-tunable strategy of Hsp70-targeting and acid-triggered self-assembled gold NPs. In vivo investigations showed that when combined with PD-1 checkpoint immunotherapy, the median survival time was significantly prolonged.^687^

Most immune cells express the TRL9 ligand known as the CpG oligonucleotide, which has been demonstrated to have the ability to initiate immunologic rejection and promote long-lasting immunity against glioma.^688,689^ In order to achieve this objective, researchers have investigated the use of carbon nanotubes loaded with CpG to efficiently treat gliomas through intracerebral administration.^690–692^ Compared with other nanocarriers, high-density lipoprotein (HDL) has a longer half-life and can circulate in plasma for up to 3–4 days.^693^ Hence, Kadiyala et al. created glioblastoma-targeted chemo-immunotherapy carriers utilizing sHDL nanodiscs containing CpG, a Toll-like receptor 9 (TLR9) stimulant, and docetaxel (DTX), a chemotherapy drug. Upon delivery of the DTX-sHDL-CpG nanodiscs to the tumor site, the findings revealed the initiation of tumor-specific T cells through the activation of tumor antigen-presenting dendritic cells and macrophages. This subsequently triggered both innate and acquired immune responses within the tumor microenvironment, with no apparent adverse effects observed. Furthermore, the combination of DTX-sHDL-CpG treatment and radiation therapy led to the regression of 80% of glioblastoma tumors in animals, ensuring their long-term survival. Notably, the mice remained completely free of tumors even when tumor cells reinvaded the opposite cerebral hemisphere, suggesting the development of immune memory against glioblastoma.^694^ To summarize, the utilization of radiotherapy in conjunction with this glioblastoma therapeutic approach holds significant promise for tumor regression, prolonged survival, and the development of immune memory.

### The challenges in diagnosis and treatment

Currently, surgical resection is listed as the preferred treatment for glioblastoma. However, because most surgical resections are incomplete and glioblastoma is highly invasive, residual cells often result in a dismal prognosis and poor quality of life.^695^ Cancer cells can be killed by radiation with high energy (~MeV) ionizing radiation deep into the brain, but the effects of radiation are often weakened by the hypoxia tumor microenvironment (TME) and intrinsic radiation resistance.^696^ Meanwhile, chemotherapy is controlled by many factors, including toxicity to normal cells, chemotherapeutic resistance of tumor cells, the existence of glioma stem cells, the blood-brain barrier (BBB), etc.,^697,698^ among which the existence of the BBB plays an important role in the chemotherapy process. In addition to the therapies listed above, researchers are working on the treatment of glioblastoma with cytotoxic precursor drugs, angiogenesis inhibitors, PI3K/Akt/mTOR pathway inhibitors, matrix MMP inhibitors, and other drugs.^699–702^ At present, the toughest challenges in the diagnosis and treatment of glioblastoma mainly lie in the following aspects: the blood-brain barrier, perivascular/perineural microinvasion, hypoxia microenvironment, drug resistance, intratumoral molecular heterogeneity, etc.^632,703,704^ We will present the below contents from the facing difficulties.

#### Blood–brain barrier (BBB)

In the late 1800s, Paul Ehrlich unintentionally stumbled upon the fact that when dye was injected intravenously into laboratory animals using in vivo staining methods, all organs except the brain became colored. Thus, the veil of BBB was adjourned. In the BBB, tightly connected brain capillary endothelial cells form tight intercellular junctions with low transcytosis and no perforated proteins. Preservation primarily occurs via interactions involving mural cells, glial cells, immune cells, and nerve cells. The BBB effectively controls the transportation of molecules, ions, and cells,^705^ ensuring the brain’s protection against harmful substances (including medications) and pathogens present in the bloodstream. Additionally, it supplies essential nutrients like glucose and amino acids to support proper functioning. Carrier-mediated transport (CMT) plays a crucial role in maintaining the brain’s steady state by utilizing the BBB.^635,706–708^

Due to the difference between the endothelial cells and peripheral cells in BBB, it is difficult for drugs and other chemicals to cross BBB, and it is one of the main obstacles to the development of treatments for brain tumors such as glioblastoma.^709^ The advancement in comprehending the biology of BBB has resulted in the creation of innovative approaches for administering medication.^710^ It is widely acknowledged that a solid tumor typically possesses “leakage” blood vessels that enhance the drug’s permeability and retention capacity, known as the EPR effect.^711^ However, various types of gliomas still have a blood-brain tumor barrier, which makes them resistant to chemotherapy, so people try to use a variety of methods to bypass the BBB for drug delivery. One of the most common methods is chemotherapy. On the one hand, by increasing the lipophilicity of the reagent and using non-polar materials with small molecular particle sizes, the polarity and ability to form hydrogen bonds can be weakened to pass more easily through BBB endothelial cells.^701,712^ BBB is home to large quantities of CMT and receptor-mediated transport (RMT) that can also be treated using a “Trojan Horse” strategy in conjunction with nanotechnology.^713^ However, it is important to consider the impact of the BBB drug efflux pump on drug transportation. In BBB endothelial cells and glioblastoma, a p-glycoprotein with a molecular weight of 170 kDa is present on the cell membrane. This protein belongs to the ATP-binding cassette (ABC) transporter superfamily and has the ability to actively remove various substances from cells. It plays a role in the transportation of chemotherapy drugs across the BBB into the brain and contributes to the development of chemotherapeutic resistance in glioma cells.^635,714^ At present, some physical strategies have gradually entered people’s vision, such as radiation, electroporation, focused ultrasound, and so on.^715^ Although these methods can bypass the BBB and increase the local concentration of the drug in the brain, they are highly invasive and lead to an increased risk of brain infection.

#### The microenvironment of glioblastoma

The glioblastoma microenvironment consists of both cellular and non-cellular elements. The cellular elements comprise neurons, astrocytes, endothelial cells, and immune cells, whereas the non-cellular elements consist of diverse ECM components, fluids, chemokines, cytokines, and other soluble factors.^716^ Studies have shown that the invasion of GBM is the result of a continuous two-way interaction between tumor cells and their microenvironment.^717^ Glioma cells release various soluble factors that attract different cell types to participate in the TME, such as astrocytes, endothelial cells, and numerous other immune cells. This process subsequently stimulates the local synthesis of cytokines, chemokines, and growth factors. Afterwards, these cytokines engage with components of the extracellular matrix (ECM) to ultimately impact the phenotypic alterations of immune cells and facilitate the growth and spread of glioblastoma.^718,719^ Drug resistance and tumor recurrence are the consequences of the proliferation of glioblastoma and its interaction with the tumor microenvironment.^720^ Most of the tumor microenvironment is composed of inflammatory infiltrates dominated by microglia and macrophages.^721^ Every complex substance in the tumor microenvironment plays a role in hindering immune recognition and response and promoting the rapid proliferation of tumor cells and infiltration and the invasion of normal tissues.

In glioblastoma, there exists a profoundly immunosuppressive microenvironment where tumor-associated macrophages (TAMs) play a crucial role as the primary infiltrating immune cell population. TAMs engage in interactions with tumor cells, facilitating their progression and advancement. In general, TAMs can be categorized as M1 macrophages, which are considered “classical activated” anti-tumor cells, and M2 macrophages, which are known as “alternative activated” cells that promote tumor growth. Among them, TLR ligand and IFN stimulate the production of M1 macrophages and induce the production of IFNγ, IL12, etc., forming a positive feedback loop. M2 macrophages are driven by IL4, IL13, transforming growth factor, and glucocorticoids, and then IL18, and IL22 are produced to enhance the function of NK cells. Studies have shown that tumor-promoting TAMs have stronger phagocytic ability.^722–725^ The migration and invasion capabilities of glioma-related microglia and macrophages are enhanced during the polarization process of M1/M2, thanks to the release of lactadherin and osteopontin by tumor cells, which promote the contraction of actin filaments and rearrangement of microtubules. Simultaneously, research has indicated that TGF-β derived from glioblastoma (glioma-associated microglia/macrophages) can enhance the expression of MMP-2 in the matrix, consequently facilitating the deposition of ECM and encouraging the invasion of glioma.^726,727^ Recently, the composition of TME and the number of cytotoxic CD8 T cells, or regulatory T cells, have been defined as predictive markers of the survival and treatment responses of various solid tumors, including gliomas.^639^ Recently, many new immunotherapies against glioblastoma that have been established for the adaptive immune system play a therapeutic role by regulating the tumor microenvironment.^724,728^ A better comprehension of the infiltration of immune cells in the glioblastoma microenvironment enables the improved development of therapeutic approaches that utilize immune checkpoint inhibitors (ICI) for regulating the immune response towards glioblastoma.^729^ In addition, based on some characteristics of the tumor microenvironment, such as tumor internal pressure, hypoxia, acidic environment, etc., special nanomaterials that can respond to these environments can be used to enhance tumor uptake of nanoparticles, thus enriching the lesion area to achieve a better effect.^730^

The emergence of nanotechnology has opened up new windows for treating various diseases, and nanomaterials have received more and more attention in the delivery of drugs or genes. Therapeutic NPs originated in the 1950s with Jatzkewitz’s development of a polymer-drug conjugate, followed by Bangham’s discovery of liposomes in the mid-1960s. Many nanomaterials, such as inorganic NPs, polymer NPs, etc., are designed for the treatment of diseases and show great advantages, such as passive and active targeting, sustained drug release, low toxicity, etc.^731^ Effective delivery of brain tumors has been reported using polymer NPs, micelles, liposomes, protein nanocages, and inorganic NPs in the past few years.^732^ Researchers have been able to customize NPs for brain tumor accumulation by utilizing advancements in nanotechnology, which allow for adjustments in size, shape, body density, lipophilicity, and surface chemistry.^733^ Studies have shown that particles with a size of 50–200 nm and neutral or negative zeta potential have the best effect, while NPs with higher potentials will damage the BBB. On this basis, Lundy et al.^734^ found that a small-dose intravenous injection of VEGF can temporarily enhance the permeability of the BBB and use this short-term opportunity to deliver nanomedicine to the brain tissue for the treatment of glioblastoma. In addition, NP-mediated drug delivery can also significantly reduce the required dose of drugs, stabilize the drug in the blood, increase the bioavailability of the drug, and decrease side effects. Here, we will summarize the relevant research progress of nanotechnology in the treatment of glioblastoma from several aspects, such as detection, diagnosis, and treatment.

## Nanomedicine in hematological malignancies

Hematological malignancies (HM) are mainly divided into three types, including leukemia, lymphoma and multiple myeloma. HM is responsible for a higher number of fatalities compared to all other types of cancers among children, adolescents, and young adults.^735^ Tumor localization primarily occurs in the bone marrow, as well as in peripheral blood and secondary lymphatic organs like the spleen and lymph nodes. Current therapy for HM includes chemotherapy, hematopoietic stem cell transplantation, radiotherapy, and immunotherapy. Although their therapeutic effects have been proven in numerous studies, they are still challenging in clinical application to date.^736^ Therefore, it is urgent to explore an appropriate drug/molecular delivery system to improve the therapeutic effects and safety.

Employing nanotechnology has been demonstrated to be effective in cancer therapy.^737,738^ The research of nanotechnology in treating hematological tumors has received widespread attention. In this part, we discuss the progress of nanotechnology on hematological malignancies in recent years.

### Diagnosis

Early diagnosis of leukemia is crucial to prevent irreversible damage and mortality. Currently, leukemia is diagnosed primarily by examining peripheral blood and bone marrow, which involves bone marrow biopsy, complete blood count (CBC), and analysis of blood smears. The first technique is an extremely invasive process with complications, whereas the second one is non-invasive but typically lacks sensitivity for early detection. The advent of nanotechnology has paved the way for a fresh avenue in the diagnosis of leukemia.

Nanosized biomarkers known as extracellular vesicles (EV), such as exosomes, are considered highly promising ways for cancer diagnosis. The molecular characteristics obtained from disease-specific extracellular vesicles found in blood or other bodily fluids can assist in the diagnosis and prognosis of leukemia. However, examining the EV data specific to the disease remains a difficult task. Belov et al. developed an antibody microarray (DotScan) that comprises a collection of fixed antibodies capable of identifying clusters of antigens present on the exterior of EVs derived from tumors. This technique can be utilized for the extraction and examination of the surface protein expression pattern of extracellular vesicles obtained from cancer cells or the plasma of patients with chronic lymphocytic leukemia (CLL).^739^ DotScan has introduced a novel approach for the detection and identification of leukemia. Nevertheless, the limited concentration of cancer-derived exosomes in bodily fluids restricts their potential use. Efficient acquirement of EV is still a challenging task. To solve this problem, Lin et al. developed a fluorescent biosensing platform. In their design, leukemia-derived exosomes can be captured by anti-CD63 antibody on the platform. The exosomes were bound by a set of DNA primers that specifically targeted the nucleolin-recognition aptamer (AS1411), initiating the amplification reaction and resulting in the production of a significant number of repeated sequences. Subsequent repetitions were hybridized with GNP-DNA-FAM, a gold nanoparticle conjugated with FAM fluorescent dye. The accumulated fluorescence signals from FAM would then be detected.^740^

The mutation status of the immunoglobulin heavy-chain variable region (IgVH) gene is a robust predictor of leukemia prognosis. The expression level ZAP-70 gene, which encodes a type of tyrosine kinase, is the most significant difference between the mutated lgVH and unmutated lgVH in CLL patients. Therefore, the expression level of ZAP-70 gene can be used to accurately predict the lgVH gene mutation status. Based on the features of ZAP-70 gene in CLL patients, Ensafi et al. designed a biosensor for the identification and differentiation of two variations of CLL (IgVH gene mutation status). A highly sensitive conversion platform for detecting the level of ZAP-70 gene and distinguishing CLL types through genetic analysis is created using a gold NPs (AuNPs) coated with ZAP-70 oligonucleotide probe on the surface of the biosensor.^741^ Furthermore, caspase-3, a well-established indicator of cellular programmed cell death, can be triggered in both the intrinsic and extrinsic pathways of apoptosis. Detecting the activity of caspase-3 allows for the evaluation of the impact of chemotherapy. Wen et al. introduced a straightforward and precise technique for surface-enhanced Raman scattering (SERS) utilizing interconnected plasmonic bio-interfaces. When caspase-3 is present, it can specifically identify and cut the N-terminus of the biotinylated DEVD-peptide that is immobilized on the SERS substrate. The remaining peptide then attaches to the streptavidin-modified AuNPs probe through the biotin-streptavidin interaction. The interaction between the electromagnetic field of the titanium dioxide nanotube arrays modified with gold nanoparticles can significantly amplify the Raman signal of the reporter molecule, thereby indicating caspase-3 activity. The broad application prospects of this technique in monitoring cell apoptosis are widely approved.^742^

Multiple myeloma (MM) is an incurable disease of plasma cell disorder. This condition is marked by the uncontrollable proliferation of monoclonal plasma cells within the bone marrow, leading to excessive production of inefficient immunoglobulin or immunoglobulin chains. The buildup of these antibodies and the interaction of atypical monoclonal plasma cells with other cells in the bone marrow can result in a range of symptoms, including debilitating fractures, infections, anemia, fatigue, pain, renal failure, and hypercalcemia. The incidence of MM is age-related, and it occurs mostly in people over 40 years old, especially those over 60 years old. The growth and expansion of myeloma cells occur predominantly within the bone marrow,^743^ highlighting the crucial role of the bone marrow microenvironment in facilitating the proliferation and viability of myeloma cells. Within this small-scale environment, intricate relationships occur between various cell types, involving both reciprocal encouragement and discouragement. The functional components of the bone marrow microenvironment can be classified into cellular and non-cellular compartments. The progression of MM involves the interaction of various components within each compartment, each playing distinct roles.^744^

Currently, the staging and assessment of MM have consistently depended on the examination of bone marrow and blood samples using molecular and flow cytometric analysis.^745^ However, these tests have overlooked the spatial heterogeneity of the disease within the patient. The successful utilization of clinical diagnostic imaging techniques has enabled the prediction, evaluation, and monitoring of the disease. Nonetheless, the development of collaborative or supplementary detection technologies remains essential in order to establish non-intrusive approaches that allow for more accurate and frequent monitoring of disease activity.^746,747^

Enhanced imaging of living tissues at the cellular level is frequently achieved through the use of optical coherence tomography (OCT), a non-invasive technique. Nevertheless, like other imaging methods that rely on coherence detection, the presence of speckle noise hinders the ability of OCT to provide cellular-level diagnostics due to its negative impact on spatial resolution. In speckle modulation OCT, a contrast agent made of gold nanorods was employed by a research group to identify individual polystyrene beads and myeloma cells of micron size within the bloodstream. The initial report demonstrates that OCT has the capability to identify individual cells in the bloodstream, creating a fresh opportunity for the real-time identification and measurement of circulating tumor cells in living organisms.^748^ MRI, in comparison to CT, SPECT, or PET, has a superior ability to differentiate between benign and malignant lesions, and it enables earlier detection of bone marrow infiltration. Nevertheless, MRI is a lengthy and costly procedure that depends on the passive buildup of contrast agents not specifically targeted to the tumor microenvironment. This significantly hampers the accuracy and effectiveness of MRI detection. Detappe et al. created nano-sized gadolinium-based particles (<5 nm) that were attached to full-length antibodies targeting B cell maturation antigen (BCMA), demonstrating fast absorption by tumors and enhanced accuracy in detecting multiple myeloma.^747^

In individuals diagnosed with multiple myeloma, plasma cells will generate a significant quantity of unbound immunoglobulin light chains, a substantial portion of which are unable to be utilized for the formation of complete immunoglobulin molecules. The surplus light chains in serum can be filtered into urine through the glomerulus, and the detection of Bence-Jones protein in urine is typically regarded as a diagnostic biomarker for multiple myeloma in clinical practice. Nevertheless, the conventional method for detecting protein in urine is not highly sensitive and requires a lot of effort. Recently, a research team has developed a new method that was demonstrated to be faster, more economical and convenient compared to traditional methods. They utilized macroporous silica foams (MOSF), a type of silica foam with a specific structure, to concentrate proteins in urine. Subsequently, they were able to identify and examine the composites of materials and proteins, which included Bence-Jones protein, using MALDI-TOF MS, a technique called matrix-assisted laser desorption ionization time-of-flight mass spectrometry.^749^ This design provides a new option for clinical diagnosis of Bence-Jones protein.

Exosomes derived from tumors are becoming biomarkers that are emerging for personalized diagnosis of medical tumors. Nevertheless, there exists a dearth of effective technological frameworks for the isolation and characterization of exosomes. A research team has proposed a new method for detecting exosomes from MM. In their studies, Di Noto et al. designed a functionalized surface plasma resonance (SPR) biosensor chip combined with colloidal gold nanoplasmonics. By utilizing this nanomaterial, it was discovered that only exosomes derived from MM have the ability to attach to heparin. Heparin is a structural analog of heparan sulfate proteoglycans (HSPGs) and is recognized to be involved in facilitating exosome endocytosis. The utilization of this approach supplements the traditional biochemical analysis technique for exosomes and presents a fresh concept for the diagnosis of MM.^750^ Iaccino et al. discovered that MM exosomes could express the B cell receptor for immunoglobulin (Ig-BCR) and could be targeted by specific binding peptides (Id-peptides).^751^ Identifying MM-released exosomes using heparin or the Id peptide could potentially serve as a highly sensitive diagnostic approach for evaluating disease progression in the clinical setting.

Recently, Tang et al. developed a thermosensitive nano-platform that can simultaneously image and treat MM. This platform includes titanium dioxide (TiO2) NPs that can collect ultraviolet rays from Cherenkov radiation to stimulate the production of ROS to induce the death of cancer cells. This platform was covered with a cancer-focusing substance called transferrin (Tf) and labeled with a radioactive element (89Zr) to specifically target the bone marrow. This method can effectively inhibit tumor growth and achieve a combination of diagnosis/treatment. It provides an effective model for treating MM and other bone-related malignancies.^752^

### Treatment

Leukemia is one of the most prevalent fatal types of HM. Leukemia is classified into acute and chronic types based on the level of differentiation and the duration of the disease’s progression. The majority of leukemia cases are attributed to acute leukemia (AL), which can typically be categorized into two primary forms: acute lymphoblastic leukemia (ALL) and acute myeloid leukemia (AML). AML is prevalent among adults, whereas ALL is more prevalent among children. The average survival period of AL is only approximate three months, while some patients will die even after a few days of diagnosis if they have not received treatment. Traditional treatment methods for leukemia are usually hypertoxic and will produce a series of side effects.^753^ Nanotechnology is a new and promising method for cancer treatment to improve the diagnosis and therapeutic effect of leukemia.

Lymphoma is a cancerous growth that develops in the lymphoid hematopoietic system. It is characterized primarily by painless swelling of the lymph nodes and enlargement of the liver and spleen. It is usually accompanied by systemic symptoms, including fever, night sweats, weight loss, and itching. Lymphoma is categorized into non-Hodgkin’s lymphoma (NHL) and Hodgkin’s lymphoma (HL) based on the characteristics of the tumor cells. The NHL is a collection of various independent diseases with significant heterogeneity, and its incidence is significantly higher than HL. NHL can be classified into B cell, T cell, and NK cell lymphoma based on the various sources of lymphocytes. Lymphoma has a wide variety of types and a complex pathogenesis that is not yet clear. Its pathogenesis may be related to heredity, immune, or viral infection, etc.^754^ Current treatments for lymphoma mainly include radiotherapy, chemotherapy, hematopoietic stem cell transplantation, and immunotherapy, such as CAR-T cells therapy.^755–757^ Nanomedicine technology has also been made great progress for the treatment of lymphoma in recent years.

Although the survival rates of patients with MM are significantly improved, the brief infusion of small-molecule drugs into the bone marrow fails to allow sufficient time for the augmentation of MM cell-drug interactions. Consequently, some myeloma cells manage to evade initial treatment.^747,752^ Over the last decade, there has been a growing validation of novel molecularly targeted treatments for MM, including immunomodulatory medications, proteasome inhibitors, inhibitors of histone deacetylation, as well as monoclonal antibodies targeting MM cell surface proteins or immune cell lineages. Although current treatments provide multiple opportunities to improve patients’ condition, there are still some challenges to be faced. It is necessary to find effective methods to overcome the toxicity of therapies and establish more accurate biomarkers for evaluating the effect of treatment.^746^

#### Chemotherapy

Chemotherapy drugs used in clinic have obvious limitations, such as poor pharmacokinetics, reduced bioavailability, lack of specific targeting and toxic side effects. Moreover, unexpected drug resistance often occurs. Hence, a targeted drug delivery system for leukemia cells can effectively minimize toxicity and provide a significant benefit in the treatment of leukemia. During recent years, great progress has been made in utilizing nanomedicine technology in leukemia chemotherapy.

Numerous nanomaterials have been created to improve the precision of targeting and minimize the adverse effects caused by toxicity. Auristatin E is a cytotoxic tubulin modifier which has anti-tumor activity. Pallarès et al. developed a new type of T22-GFP-H6-Auristatin nanoconjugate for targeted administration of Auristatin E to selectively eliminate chemokine receptor type 4 (CXCR4) positive AML cells. In the AML animal model, the use of NPs greatly decreased the presence of leukemia cells in both the bone marrow and circulating blood, effectively preventing the spread of leukemia cells to extramedullary organs.^758^ To obtain intelligent NPs, the researchers loaded FA-HSA-ATO, which stands for folic acid (FA)-labeled human serum albumin (HSA), with ATO. The study showcased that FA-HSA-ATO exhibited a distinct ability to identify CML cells that express folate receptor-β. Consequently, this recognition led to increased accumulation of ATO within the cells.^759^ In addition, Yasinska et al. utilized platforms consisting of gold nanoparticles with a size of 5 nanometers, coated with rapamycin, for the precise targeting of cancer cells that express the immune receptor Tim-3, employing single chain antibodies. The nanoconjugate facilitated precise and effective transportation of cytotoxic rapamycin to malignant blood cells in humans, offering potential for targeted therapy of AML.^760^ Activatable aptamers are being recognized as highly promising molecular instruments for the treatment of cancer, and Lei et al. developed a novel triangular DNA nanoarchitecture (NTri) as a prototype framework, equipping the NTri with numerous divided activatable aptamer probes (SAAPs) in a controllable manner regarding their location and alignment. NTri-SAAP exhibited a significantly enhanced affinity for the target, extremely low background interference, and strong durability in challenging environments, enabling the visualization of tumors with improved contrast for a prolonged duration of eight hours. Furthermore, NTri-SAAP has the potential to greatly enhance the body’s loading capacity and anti-cancer effect of DOX.^761^

Primary or acquired multi-drug resistance (MDR) makes most human cancers untreatable. Therefore, it is necessary to explore a reliable strategy to avoid drug resistance. Bellavia et al. employed exosomes function as a vehicle for transporting therapeutic substances through the loading of diverse molecules. They used genetic engineering technology, to promote HEK293T cells to produce exosomes that are rich with Lamp2b protein and interleukin 3 (IL3). Meanwhile, they also designed exosomes to load the chemotherapeutic drug imatinib. The results demonstrated that the modified exosomes have the ability to successfully suppress the proliferation of cancer cells in vitro/in vivo and overcome the resistance of cancer cells to chemotherapy.^762^ Due to its intrinsic ability to intercalate, daunorubicin is highly compatible with DNA-mediated delivery. Halley et al. employed a DNA nanostructure, created through the process of scaffolded DNA origami self-assembly, for the transportation of daunorubicin. The DNA nanostructure exhibited the ability to overcome drug resistance in an animal model of leukemia that was resistant to the drug. The research foundation provided by this design enables the clinical application of a drug delivery system to address the issue of drug resistance in leukemia and other types of blood cancers.^763^

Hematological neoplasms differ from solid tumors as they lack a distinct form and specific site, with tumor cells dispersing throughout the circulatory system. Hence, in the development of nanomaterials, addressing the issue of managing drug release at the targeted site and extending the duration of drug effectiveness becomes crucial for treating leukemia. Black phosphorus nanosheets (BP-NS), being a type of novel two-dimensional nanomaterial, have demonstrated exceptional drug carrier capabilities due to their superior optical characteristics and compatibility with living organisms. A team of researchers has recently suggested a combination drug nanocarrier utilizing BP-NS, capable of conducting a synergistic and focused chemo-photothermal treatment for ALL. Electrostatic adsorption allows for the loading of drugs onto the BP-NS. Enhancing the physiological stability of BP-NS, the adsorbed polyethylene glycol (PEG) layer effectively shields the interior from water and air damage. Furthermore, the pH and NIR lasers can serve as triggers for the drug release from the nanocarrier. By employing a dual-stimulus responsive drug delivery system, the potential for drug leakage can be effectively mitigated, consequently leading to a reduction in adverse effects.^764^ Boto et al. created NPs with the ability to store retinoic acid (RA) in leukemia cell cytoplasm for extended periods, utilizing light as a trigger. Upon being exposed to the blue/UV light, the RA will be released within a few minutes. Of greater significance, leukemia cells that have been transfected with light-responsive RA-NPs have the ability to migrate to the bone marrow in living organisms, undergo differentiation when exposed to blue light, and secrete paracrine factors that regulate neighboring cells. By utilizing this technique, the expected outcome of controlling cell populations and regulating leukemia cells from a distance was successfully achieved.^765^ Xiong et al. utilized a blend of solvent substitution and the assistance of a static magnetic field to fabricate nanocomponents with superparamagnetic anisotropy (SAN). Vincristine (VCR) was used to build and load SANs, resulting in the formation of VCR-SANs. According to research findings, VCR-SANs exhibit fast and consistent release characteristics, along with extended duration in the bloodstream.^766^

The treatment of lymphoma has enriched new concepts and approaches due to the rapid development of nanotechnology and the introduction of novel nanomaterials in recent years. Compared with traditional drug delivery systems, nano-drug delivery systems can enhance the pharmacodynamic properties and therapeutic effects of drugs by utilizing their unique size, shape, and materials. DOX is a commonly used drug in treating human malignant tumors with strong capability in killing tumor cells. However, the drawbacks of DOX are also bothersome as it has cardiac toxicity, myelosuppression and gastrointestinal adverse reactions. The combination of DOX and NPs can improve these adverse effects and promote its therapeutic effects.^767,768^ Currently, the majority of studies in the field of lymphoma chemotherapy are primarily focused on the use of DOX for drug delivery facilitated by nanomaterials.

Srivastava et al. developed a mesoporous silica NPs-based nano-drug delivery system consisting of DOX, 5-fluoro-2-deoxy uridine, and folic acid, aimed at responding to triple stimuli. Dalton’s lymphoma, a highly aggressive murine lymphoma, was the target of delivery using this system. The fusion of active and passive targeting mechanisms provided an innovative hyper-chimeric framework for a drug delivery system that responds to stimuli. The precise delivery effect of folic acid successfully delivered DOX and 5-fluoro-2-deoxy uridine to the nearby region of cancer cells, leading to a substantial suppression of tumor cell proliferation. In comparison to the control group that did not receive treatment, the administration of the dual drug-carrying structure resulted in a significant decrease in tumor burden and enhanced the survival rate of mice. The treatment using the dual delivery system also enhanced the natural and acquired immune defense mechanisms of the animals.^769^

Zhao et al. created a versatile Apt-NMed that enables precise chemotherapy and gene therapy for ALCL. DOX and synthetic oligonucleotides were used to create Apt-NMed, which consisted of a CD30-specific aptamer and ALK-specific siRNA targeting the oncogene. Apt-NMed displayed a clearly defined nanoscale structure (with a diameter of 59 mm) and remained stable in human serum. With the assistance of the aptamer, Apt-NMed precisely attached to and entered the designated ALCL cells. By delivering Apt-NMed into the cells, it induced the swift liberation of DOX and ALK-targeted siRNA, leading to a synergistic therapeutic outcome. The survival rate of mice was greatly enhanced by Apt-NMed therapy, which offers a novel approach for precise treatment of ALCL.^770^

Goswami et al. synthesized luminescent blue copper nanoclusters (Tf-Cu) using Transferrin (Tf) as a template. They coupled DOX in spherical Tf-Cu NPs based on electrostatic interaction. In Tf-Cu, the absorption of DOX caused the blue luminescence to be quenched, resulting in a remarkable red luminescence observed in Forster resonance energy transfer (FRET). When the transferrin receptor (TfR) that was overexpressed on the cell surface internalized Tf-Cu, the cytoplasm of cancer cells regained its blue luminescence. In the core, red luminescence observed as DOX was gradually released from Tf-Cu. Biocompatible Tf-Cu showed excellent targeting efficiency for cells overexpressing TfR compared with cells expressing less TfR. The assessment of Tf-Cu in live mice with TfR-positive Dalton’s lymphoma ascites (DLA) demonstrated a notable suppression of tumor growth, leading to an extended survival period of mice.^771^

ATP is an important transmitter that mediates various biological effects in cancer by P2X7 purinoceptors. Srivastava et al. studied the anti-tumor activity of ATP-modified biomineralization and DOX mesoporous silica against Dalton’s lymphoma. The results showed that the interactions between ATP and the phosphate group on calcium carbonate prevented the rapid release of DOX in the NPs and had obvious tumor-killing effects on anti-DOX tumor cells. This composite nanomaterial had excellent biocompatibility and higher absorption capacity via the participation of purinergic receptor P2X.^772^

Xiao et al. developed a micellar formulation of DOX (DOX-DCMs) with reversibly disulfide cross-linking to target B-cell lymphoma. The DOX-DCMs exhibited a high capacity for drug encapsulation, an ideal particle size ranging from 15 to 20 nm, excellent stability in human plasma, and a drug release profile that is triggered by reducing conditions. The pharmacokinetics of DOX were greatly enhanced by DOX-DCMs, with its elimination half-life and area under the curve (AUC) being 5.5 times and 12.4 times higher than those of free DOX, respectively. The biodistribution study demonstrated that DOX-DCMs have a tendency to selectively gather at the tumor location while effectively decreasing the cardiac absorption of DOX. In a xenograft model of B-cell lymphoma in humans, DOX-DCMs demonstrated not only tumor growth inhibition and increased survival rate, but also a decrease in DOX-induced cardiotoxicity compared to equivalent amounts of free DOX and non-crosslinked DOX.^773^

Guo et al. prepared two linear-dendritic telodendrimers (TDs) based on amphiphilic riboflavin (Rf) to achieve efficient delivery of DOX. DOX encapsulation was achieved using Rf-containing nucleophiles and a hydrophilic PEG shell in the composition of Micellar TD NPs. Due to the robust interaction between DOX and Rf, as well as the amphiphilic nature of Rf, these nanocarriers exhibited exceptional drug loading capacity for DOX, prolonged drug release, and optimal particle size ranging from 20 to 40 nm. The NPs have successfully targeted various tumors in living organisms and greatly prolonged the duration of DOX in the bloodstream, making them worth further development and utilization.^774^

Sedlacek et al. developed and created a novel transportation mechanism for DOX utilizing a biocompatible hydrophilic poly(2-ethyl-2-oxazoline) (PEtOx) carrier with a linear structure and precise molar mass range. The medication was connected to the polymer structure through an acid-responsive hydrazone, enabling its controlled release within the tumor. Experiments conducted in live mice with EL4 lymphoma demonstrated that the PEtOx conjugated with a higher molecular weight polymer (40 kDa) exhibited extended blood circulation, enhanced tumor aggregation, and improved anti-tumor efficacy when compared to that with lower molecular weight polymer (20 kDa). Besides, the synthesis of PEtOx is simple, making it a possible alternative for drug delivery.^775^

Significant advancements have been achieved in the management of lymphoma through the utilization of combining radiotherapy and chemotherapy. Man et al. selected Dox NPs, which are encapsulated with a ligand called SHAL and have a high affinity, for the treatment of tumors overexpressed HLA-DR antigen. Encapsulated DOX was delivered by the internalized NPs to cells expressing HLA-DR, resulting in enhanced immunogenic cell death. In addition to promoting ICD, the DOX also enhanced the susceptibility of cancer cells to radiation by causing cell cycle halt and inhibiting the restoration of DNA harm. In vivo studies on biodistribution and toxicity confirmed that the targeted NPs improved tumor uptake of DOX while decreasing its systemic toxicity.^776^

Within the bone marrow, which has a dense network of blood vessels and a porous texture, NPs can serve as a smart method of delivering drugs. This allows them to reach deeper into different compartments of the bone marrow, ultimately improving the efficiency of drug delivery and enhancing their ability to target specific areas. MM can be treated with Bortezomib (BTZ), which is a novel proteasome inhibitor. Nevertheless, the administration by standard intravenous or subcutaneous injection can lead to numerous undesirable side effects. It is imperative to urgently address the clinical constraints associated with the utilization of anticancer medications like BTZ. Nigro et al. have created a nanodevice made of mesoporous silicon that includes BTZ. This nanodevice has the ability to release the drug in an acidic setting and specifically target folic acid. The folate receptor (FR) overexpressing on MM cells’ membrane can selectively identify BTZ, without harming or disrupting the metabolic balance of FR-negative healthy cells. Anticipated to efficiently deliver medications at the tumor microenvironment, this device that can be adjusted for pH is designed for acidic tumor microenvironments.^777^ Lee et al. also designed a pH-sensitive nanomaterial to control BTZ release in cells. The micelle/hydrogel composites were created by combining phenylboronic acid (PBA) and catechol through a covalent bond to encapsulate BTZ within micellar NPs. These micelles, loaded with the drug, were then integrated into hydrogels, which have a core made of catechol-functionalized polycarbonate. When the composite material was circulated in the blood, the borate bond would be dissociated in the lysosome (pH 4.5–6.0) of cells to release the loaded drug. The composite of micelle/hydrogel loaded with BTZ can serve as a layer for storing drugs or as a barrier for diffusion, enabling a continuous release of medication and extending the duration of blood circulation.^778^ De la Puente et al. utilized chitosan NPs that target CD38 to enhance the effectiveness and targeting efficiency of BTZ in MM cells. This approach also increased the proteasome-inhibitory activity and specificity of BTZ through CD38-mediated endocytosis. These findings suggest that this method holds promise as a potential treatment for MM.^779^ One factor that leads to poor treatment of MM is the emergence of thrombotic complications. Developing a novel method for delivering drugs can enhance the existing treatment protocol and eradicate the associated complications, thereby providing significant advantages in managing MM. Hu et al. created a nanocarrier with a core-shell structure by enveloping the polymeric NPs’ surface with a platelet membrane. The NPs possessed characteristics that allowed them to specifically target bone marrow, effectively delivering BTZ/tissue plasminogen activator (tPA) and enhancing drug accumulation in bone marrow locations. This approach reduces unintended effects and eliminates thrombotic complications, while also inhibits the progression of MM.^780^ Swami et al. engineered polymeric NPs specifically designed to target bone marrow and deliver therapeutics BTZ in a controlled manner over time. The NPs consisted of PLGA, PEG and bisphosphonate. On one hand, PEG can significantly reduce immune recognition and enhance circulation in the body. On the other hand, bisphosphonate molecules that chelate calcium ions are a group of encouraging ligands capable of reducing off-target effects and enhancing localized drug concentration. By enhancing pharmacokinetics and biodistribution, this delivery system holds the promise of enhancing drug utilization, offering specificity to the bone microenvironment and minimizing off-target effects.^781^

Carfilzomib is another proteasome inhibitor approved by the FDA after BTZ. Similar with BTZ, it also has its limits in clinical application. In order to maintain the anti-tumor activity of carfilzomib and reduce toxicity, Ashley’s team used liposomal carfilzomib NPs to effectively target MM cells, thereby inhibiting the proteasome and inducing cancer cell apoptosis.^782^ It is well known that combination of drugs can exhibit a synergistic effect, but it is usually difficult to achieve due to uneven administration process, pharmacokinetic properties, and multiplied toxicity. In order to maximize the anti-tumor effect of combination therapy and improve the prognosis of patients, Ashley et al. used polyethylene glycol liposome NPs as a carrier, double-loaded with carfilzomib and adriamycin. By controlling their release rate, biodistribution and metabolism, the best synergy ratio can be obtained at the tumor site. The effect of tumor growth inhibiting in vivo by using liposomal NPs for combination therapy is better than the combined application of free drugs, while reducing systemic toxicity and improving the prognosis effectively.^783^

#### Gene therapy

Since the launch of gene therapy, some treatments have undergone clinical trials, including leukemia. This gene delivery systems have great potential and expectantly become an important tool for leukemia treatment. Compared with natural viral vectors, artificial nano-carrier is easier to prepare and modify with multiple functions.^784^ It has fine biocompatibility and reliable transfer efficiency of gene, which generally will not cause a strong immune response. Research on gene delivery using nanomaterials has gained increasing attention due to the rapid development of nanotechnology.

The fusion of the gene breakpoint cluster region (BCR) and Abelson murine leukemia viral oncogene homolog (ABL) occurs when the long arm segment of chromosome 22 translocates to the long arm of chromosome 9, leading to the manifestation of the Philadelphia chromosome. Philadelphia chromosome can be occurred in most CML, some ALL and a few AML. At present, several nano-treatment methods targeting the Philadelphia chromosome have been reported. Liu et al. reported a CRISPR/Cas9 plasmid into PEG-PLGA-based cationic lipid-assisted polymeric NPs (CLANs). The system’s gRNA has the ability to focus on the protruding fusion area of the BCR-ABL gene. The BCR-ABL fusion gene region was effectively eliminated by this system, while maintaining the regular expression of BCR and ABL genes. Following the administration of CLANs through intravenous injection, the CML mouse model showed enhanced survival due to the presence of pCas9/gRNA targeting BCR-ABL.^785^ Hong et al. created a novel combination of viral and nonviral chimeric nanoparticles (ChNPs) that effectively target both crucial BCR-ABL-related pathways. The ChNPs comprised of a core made of adeno-associated virus (AAV) that induces pro-apoptotic BIM expression and a polymeric shell that can be degraded by acid. The shell effectively silences pro-survival MCL-1 expression through the encapsulated siRNA. CHNPs have the ability to suppress the growth of Philadelphia chromosome-positive cells.^786^ Vinhas et al. designed a nanoconjugate for combined therapy of chemotherapy and BCR-ABL fusion gene targeting. The combination makes K562 cells more sensitive to chemotherapy and can overcome the resistance mechanism of imatinib, thereby providing effective treatment for patients with CML that exhibit drug tolerance.^787^

Chimeric Antigen Receptor (CAR) T cell therapy, which is based on the manipulation of a patient’s T cells in vitro, is utilized to develop precise and efficient cancer treatments. Despite the impressive outcomes observed in trials, the complexity of current CAR T cell engineering techniques hinders their widespread use in cancer therapy. This is primarily due to the requirement of viral delivery vectors and the need to produce a substantial quantity of tumor-specific T cells in vitro. Nanotechnology has been successfully utilized by some research groups to attain favorable outcomes in the in vivo modification of T cells. Smith et al. demonstrated that NPs containing DNA can effectively transport CAR genes that target leukemia into the nuclei of T-cells, resulting in long-lasting remission of the disease. To initiate the process, T cell-targeting fragments anti-CD3 were attached to the exteriors of biodegradable poly (β-amino ester)-based NPs. This attachment allowed T lymphocytes to selectively internalize the NPs through receptor-mediated endocytosis. In the meantime, the researchers equipped the specific NPs with cancer-fighting abilities by incorporating plasmid containing the leukemia-specific 194-1BBz CAR. The manufacture of these polymer NPs in a stable state is simple and cost reduction can be achieved, potentially offering a practical and widespread solution for leukemia treatment.^788^ Exploration of messenger RNA (mRNA) as a potential approach to induce temporary CAR expression in T cells has also been undertaken. Billingsley et al. developed a formulation of ionizable lipid nanoparticles (LNPs) capable of transporting CAR mRNA to T cells. The CAR expression on this platform was stimulated to the same extent as electroporation, but with significantly less toxicity, and it exhibited potent anti-cancer effects.^789^

The siRNA treatment of hematological malignancies has been confirmed unsuccessful because of the resistance of standard transfection methods. Kedmi et al. described a self-assembled modular platform capable of constructing a limitless range of siRNA targeted carriers. The platform’s self-assembly relied on lipoproteins anchored to the membrane, which were incorporated into liposomes containing siRNA and engaged with antibodies that targeted Fc domains of crystalline fragments. In vivo, the findings indicated that the specific uptake of siRNAs by various leukocyte subsets was redirected by a straightforward exchange of eight distinct monoclonal antibodies. By specifically targeting cancer cells, this platform caused cell death and enhanced the survival of mice in a xenograft model of mantle cell lymphoma. The introduction of this modular distribution system marks a significant achievement in the development of precision medicine.^790^

Non-Hodgkin B-cell lymphoma includes an aggressive and incurable form known as mantle cell lymphoma. Individuals diagnosed with mantle cell lymphoma typically exhibit advanced disease and die within ten years. The new treatment method will impact mantle cell lymphoma through a distinct mechanism. Knapp et al. produced a lipid NP that efficiently transported siRNA to JeKo-1 and MAVER-1 cell lines of mantle cell lymphoma. Three siRNAs targeting Cyclin D1, Bcl-2 and mcl1, which prevented cell apoptosis, were co-delivered to induce the silence of these three genes at the same time. Three days after transfection, nearly 75% of JeKo-1 cells underwent apoptosis. These data indicate that the multi-channel siRNA “cocktail” formulated with lipid NPs may be a useful supplement to treat mantle cell lymphoma and other aggressive cancers.^791^

By inducing functional protein expression in a dose-dependent and time-controlled manner, the delivery of mRNA into B cells can be utilized to modify and investigate these biological functions. Nevertheless, the existing in vivo mRNA delivery mechanism is incapable to transfect B lymphocytes, mainly concentrated in hepatocytes and dendritic cells. Fenton et al. described a lipid-based nanoparticle platform capable of enclosing mRNA, specifically targeting the spleen, and delivering genetic material to B lymphocytes. Significantly, this system stimulated over 85% of overall protein synthesis in the spleen. The findings suggest that the lipid NP system has the ability to regulate the location of protein activation and stimulate protein synthesis in vivo.^792^

Zilkowski et al. created neutral nanogels that can be chemically altered with peptides and are responsive to their oxidative conditions using linear thiolated poly(glycidol) (PGSH) as a carrier for delivering miR-34a.The discovery of miRNA transport and target gene inhibition in transfected drug-resistant multiple myeloma cells provides a novel approach for gene delivery and holds great significance in cancer investigation.^793^

#### Targeted therapy

With the development of molecular mechanism of leukemia, some other molecules are used as targets of nanotechnology research. FMS-like Tyrosine Kinase-3 (FLT3) is a crucial therapeutic target in AML, while the overexpression or mutations of FLT3 have been shown to occur in most cases of AML. Although multiple tyrosine kinase inhibitors against FLT3 have promising activity for AML patients in combination with chemotherapy, their efficacy remains limited mainly by common FLT3-ITD mutations. Park et al. developed a new method targeting FLT3. They designed a soluble bioactive NP fused with anti-FLT3 antibody and the elastin-like polypeptide (ELP) A192. The fusion proteins have the ability to form multi-valent NPs that possess exceptional stability and pharmacokinetic characteristics.^794^ Jiang et al. developed a new system, consisted of FLT3 ligand-conjugated G7 polyamide (PAMAM) nano-dendritic complex, to delivery miR-150 which is a key tumor suppressor and negative regulator of FLT3, which selectively and effectively target FLT3-overexpressing AML cells, significantly inhibits AML cell growth, and promotes apoptosis.^795^ One third of all cancers in humans can be attributed to the functional suppression of the tumor suppressor protein p53 caused by its two negative regulators, murine double minute2 (MDM2) and MDMX. Targeting both MDM2 and MDMX with dual specificity is an extremely promising option for cancer treatment. Nevertheless, the primary challenges in the advancement of peptide-based therapeutics remain vulnerability to proteolytic degradation within the body and the incapacity to penetrate the cellular membrane. A team of researchers developed a versatile peptide drug delivery system using nanoparticles made of fluorescent lanthanide oxyfluoride (LONp). The polypeptide antagonists of MDM2/MDMX and the monoclonal antibody targeting CD33 were coupled to LONp by metallothioic acid bonds. This NP can activate the p53 pathway by antagonizing MDM2 and MDMX, thereby inducing apoptosis of AML cells.^796^ Furthermore, a group of researchers disclosed the utilization of layer-by-layer NPs (LbL-NPs) that target both CD20 and CD44 to transport siRNA molecules targeting B-cell lymphoma 2 (BCL-2), a protein responsible for cell survival. By encapsulating siRNA within polyelectrolyte layers that cover a polymeric core, the LbL-NP offers protection against nucleases in the bloodstream.CD20 antibodies are covalently conjugated to hyaluronic acid, which forms the outermost layer and acts as a CD44-ligand. The outer layer that targets both CD20 and CD44 ensures accurate attachment to leukemia cells, effectively triggering leukemia cell apoptosis and inhibiting their proliferation.^797^

The current options available for AML treatment are restricted due to the challenge of eliminating the population of leukemia stem cells (LSCs), which are accountable for both drug resistance and relapse. Eliminating the disease entirely may require focusing on LSC as a crucial measure. Nevertheless, LSCs exhibit resistance to traditional chemotherapy treatments, necessitating innovative strategies to eradicate these cells and enhance clinical results. By employing a new targeted NP containing anti-miR-126, Dorrance et al. showed the feasibility of therapy that targets miR-126 in LSCs. Targeting miR-126 as a novel therapeutic approach enhanced the effectiveness to treat AML by focusing on LSC.^798^ In addition, Zong et al created a multi-tiered vector framework. Initially, mPEG-PLA micelles were combined with Parthenolide (PTL), which was subsequently enclosed within degradable porous silicon (pSi) particles (MSV-PTL) for protection. The MSV-PTL particles were treated with an E-selectin thioaptamer in order to direct them towards the bone marrow. This system can deliver sufficient levels of active PTL to ablate LSCs in vivo.^799^

The treatment of lymphoma has been completely altered by molecular inhibitors, which is why researchers are becoming increasingly interested in them. Zhao et al. conducted a study to develop a novel nanodrug to combat lymphoma. The nanodrug consisted of two components: (1) a central component composed of Ag-MOFs, a metal-organic framework, loaded with PFK15, an inhibitor of tumor aerobic glycolysis; and (2) an outer component made up of red blood cell membrane incorporating a CD20 aptamer, a molecule with targeting capabilities. In both in vitro and in vivo settings, this nanodrug can effectively target B-cell lymphoma with the assistance of CD20 aptamer. The findings demonstrated that the nanodrug effectively suppressed tumor growth without any apparent adverse reactions, suggesting its precise targeting of tumor cells, alteration of aerobic glycolysis, and collaborative anti-tumor effect through Ag^+^ and PFK15, making it a reliable and efficient drug for lymphoma treatment.^800^ NHL has been verified to exhibit an overactive PI3K/mTOR pathway. Due to its inadequate solubility and toxicity, the use of BEZ235, a potent inhibitor of both PI3K and mTOR, has been discontinued in initial clinical trials. Man et al. developed a NP-based pre-targeting system to target lymphoma cells which express CD20 and HLA-DR. The pre-targeting mechanism comprised of dibenzocyclooctyne-modified anti-CD20 and anti-Lym1 antibodies used as tumor-targeting elements, along with azide-modified BEZ235.The research showed that the dual antibody pre-targeting approach successfully enhanced the amount of nanoparticles remaining in the tumor cells of interest, and enhanced the anti-cancer effectiveness of BEZ235 by blocking the PI3K/mTOR pathway.^801^

STAT3 is an oncoprotein that has been shown to contribute to the resistance of MM. However, the application of STAT3 inhibitors (such as Stattic, S3I-201 and S3I-1757) in treating MM is still limited for relatively low therapeutic effects and high toxicity. Huang et al. used a new type of NPs to pack the STAT3 inhibitors, and modified these NPs with monoclonal anti-CD38 antibodies to further enhance the therapeutic efficacy of STAT3 inhibitors on MM. The results indicated that CD38-S3I-NP has better anti-tumor effect.^802^

#### Immunotherapy

Immunotherapy and tumor vaccines have completely impacted the traditional cancer treatment methods. Stimulation of the host’s immune system through cancer immunotherapy can enhance the elimination of tumors by immune-mediated mechanisms. As a result, it offers unparalleled therapeutic benefits, including full recovery and prolonged survival for patients in advanced stages. Although immunotherapy has shown remarkable and impressive clinical outcomes, its limitations encompass low response rates and potentially fatal side effects resulting from non-specific activation of the immune system. The progress of nanotechnology has led to the development and utilization of numerous nanomaterials in immunotherapy, aiming to stimulate targeted anti-cancer immune reactions and minimize overall toxicity.^803–806^

The potential of the mRNA that encodes tumor antigens is to induce a strong immune response against tumors. Verbeke et al. described a nanoparticle system known as mRNA Galsomes, which effectively delivered modified nucleoside antigen-encoding mRNA, glycolipid antigen, and immunopotentiator α-galactosylceramide (α-GC) to antigen-presenting cells through intravenous injection. The combination of a low-dose nucleoside-GC and mRNA Galsomes resulted in the activation of natural killer T cells (iNKT) in mice, leading to a targeted immune response against tumor cells. On the other hand, the authors noticed that mRNA Galsomes exhibited benefits compared to unaltered ovalbumin (OVA)-encoded mRNA cancer vaccines, as there was a 7-fold rise in the activation of specific cytotoxic T cells, iNKT cells, and NK cells. The data indicated that administering mRNA Galsomes through intravenous injection could deliver a manageable, diverse, and successful anti-cancer impact, particularly when used in conjunction with checkpoint inhibitors.^807^

Enhancing the microenvironment that inhibits tumor growth can enhance the effectiveness of vaccines against tumors. Da Silva et al. investigated the function of poly (I:C), resiquimod (R848), and CCL20 (MIP3α) in the regulation of tumor immunity. In the tumor region, therapeutic peptide vaccines were combined with biodegradable polymer nanoparticles to serve as a carrier for the gradual and continuous release of drugs, ensuring specific immunotherapy. Administering poly (I:C) or R848 NPs resulted in improved therapeutic outcomes, and when combined with MIP3α, it significantly enhanced the anti-tumor response. The proportion of surviving patients in the long run rose to 75–100%. According to the data, the simultaneous administration of NP-facilitated poly (I:C), R848, and MIP3α demonstrated an increased impact on the efficacy of cancer vaccines.^808^

The increase of tumor-induced myeloid-derived suppressor cells (MDSCs) will affect the effectiveness of tumor immunotherapy. Sasso et al. found that lipid NPs (LNCs) loaded with lauroyl-modified gemcitabine (GemC12) can effectively target MDSC subpopulations. Subcutaneous injection with GemC12-loaded LNCs can reduce the percentage of tumor infiltrating mesenchymal stem cells, which had a higher therapeutic effect than free gemcitabine. GemC12-LNCs administered at very low dose can attenuate tumor-related immunosuppression and improve the efficacy of T cell therapy.^809^

#### Other therapy

Recently, novel nanomaterials have been continuously developed. Magnetic hyperthermia has become an innovative effective method for tumor treatment and has made breakthrough progress. Improving the ability of magnetic nanoparticles (MNPs) to target cancer cells and precisely regulating tumor cell temperature while preserving the integrity of surrounding healthy cells and tissues are the primary challenges faced by magnetic hyperthermia in cancer treatment.^810^ Faruque et al. have utilized the technique of immobilizing epithelial cell adhesion molecule (EpCAM) antibodies on the surface of MNPs to increase the selectivity of MNPs towards leukemia cells. This method can selectively remove leukemia cells selectively from the blood through magnetic hyperthermia, with almost no effect on normal cells and tissues.^811^

Peuler et al. designed a layered NP composed of cationic macromer and anionic polysaccharide at the center. A clickable polysaccharide was applied to the NPs’ surface, enabling bioconjugation with various drugs using the norbornene-tetrazine click chemistry. Enhanced protein sequestration, selective cell targeting, and controlled loading and release of chemotherapy drugs have been shown to be benefits of these layered NPs.^812^ Jin et al. developed a non-toxic and natural biocompatible polysaccharide nanotube Se-containing nanoparticles. The stable inclusion compound BFP-Se is formed by the SeNPs embedded in the nanotubes, which consist of the triple helix β-(1,3)-D-glucan (BFP) derived from the black fungus. It has the ability to greatly inhibit the growth of AML cells and enhance the cytotoxicity, offering a novel approach to treat AML.^813^ Ferumoxytol, a nanoparticle composed of iron oxide, is currently used in clinic to treat patients with iron deficiency anemia. Ferumoxytol consists of a combination of ferrous (Fe2^+^) and ferric iron (Fe3^+^), and when peroxides are present, both types of iron can generate detrimental ROS through the Fenton reaction. Trujillo-Alonso et al. demonstrated that both in vitro and in vivo, leukemic cells and leukemic stem/progenitor cells exhibit sensitivity to the production of ROS by ferumoxytol. This study first demonstrated the capacity to selectively target leukemia cells using oxidative ferrotherapy by the delivery of ferumoxytol.^814^ Shen et al. described a therapeutic system (HDL-AuNPs-BMS) for AML that utilized AuNPs and targeted the AML-promoting factor fatty acid-binding protein 4 using BMS309403 (BMS), a small molecule with selective inhibition properties. The initial evidence demonstrates that HDL-AuNPs serve as a successful vehicle for AML treatment, promoting AML cell differentiation and minimizing AML disease progression without any apparent adverse reactions. This novel approach shows promising prospects in the management of leukemia.^815^ Durfee et al. examined a protocell composed of lipid bilayers supported by mesoporous silica nanoparticles (MSN). The targeting agents used for modification of this nanocarrier were anti-EGFR antibodies. It has been proven to have low immunogenicity, constant circulation time and some other advantages.^816^

Au et al. reported a novel radioimmunotherapy utilizing nanotechnology for the management of NHL. The pre-targeting setup consisted of an anti-CD20 antibody functionalized with dibenzylcyclooctyne (DBCO) and azide-yttrium-90-(90Y) bifunctional dendrimer. The findings demonstrated that the pre-target mechanism effectively transported radioactive substances exclusively to the tumor cells being targeted, thereby amplifying the anti-cancer effects. This outcome was verified through experiments conducted on mouse models with NHL xenotransplantation.^817^

Nanomaterials-based photothermal therapy (PTT) has gained significant attention in recent years as a means to minimize the adverse effects of radiotherapy and chemotherapy. Zuo et al. reported a platelet membrane acted as a nanocarrier to co-load W18O49 NPs and metformin. The platelet membrane provides protection against oxidation and immune evasion for W18O49. Additionally, it can enhance the buildup of W18O49 in the cancerous region through both the passive EPR effect and the active bonding between platelets and malignant cells. The experiments provided evidence that this substance has the ability to effectively suppress the growth of tumors and induce apoptosis in tumor cells.^818^ Optical imaging-guidance of indocyanine green (ICG) for PTT has great potential in treating tumors. However, the traditional optical image guidance method makes living tissues produce strong tissue autofluorescence, which makes it impossible to carry out precise infrared light irradiation. Zheng et al. designed a ICG and PLPs co-doped mesoporous silica nanocarriers for continuous luminescence imaging of PTT.^819^ The findings indicated that this nanocarrier greatly enhanced the ratio of signal-to-noise in PTT luminescence imaging and the precision of photothermal treatment of tumor in vivo.

The presence of hematopoietic stem cells and progenitor cells in the bone marrow poses a significant challenge for selectively eliminating cancer cells in MM.^820^ Increasing targeting and cellular uptake are essential to be solved. In order to solve the problem of drug targeting, VLA-4 is used as a targeting ligand, which can selectively target MM cells and inhibit the cell adhesion-mediated drug resistance. Stefanick et al. developed liposome NPs with accurate stoichiometry of targeting peptides, ensuring consistent high purity without variations between batches. The liposomes were modified by attaching a cyclic late antigen-4 (VLA-4) antagonist peptide, which was modified using EG peptide-linkers and a short oligolysine chain. This modification was done to improve the liposomes’ hydrophilia and the ability to target tumor.^821^ Furthermore, they dual-targeted VLA-4 and lpam1 to prepare functionalized liposomes by two peptide antagonists that tend to bind to cancer cells to produce synergistic effects while minimizing damage to healthy cells and tissues. Compared with the traditional single-receptor targeting methods, it has higher selectivity and improves the targeting problem of cancer treatment.^822^ Kiziltepe et al. have designed and developed a multifunctional NP that combined with traditional chemotherapy and VLA-4 targeting. Polyethylene glycol micellar NPs are used as a drug delivery system, combined with DOX and VLA-4. The accumulation of NPs in tumors was enhanced. A promoted tumor growth inhibition and reduced systemic toxicity were also observed. The study offers a preclinical theoretical foundation for assessing multifunctional NPs’ efficacy in improving tumor targeting.^823^

By combining direct cytotoxicity, immune-stimulatory, and antiangiogenic mechanisms, phototherapy (PT) can provide precise and controlled tumor killing with high spatiotemporal accuracy. Kotagiri et al. successfully transformed chemotherapeutic medications into light-activated drugs with spatial and temporal functions. Additionally, they introduced untreated hydrophobic photosensitive drugs to lipid nanomicelles specifically designed to target tumors or human serum protein NPs. It can selectively deliver drugs to cancer cells and effectively inhibit the proliferation of diffuse multiple myeloma using Cerenkov radiation induction therapy (CRIT).^824^

In addition to the use of phototherapy and magnetic hyperthermia, immunotherapy has also made significant progress in cancer treatment. B cell maturation antigen (BCMA) belongs to the superfamily of tumor necrosis factor (TNF) receptors. Due to its limited expression on MM and plasma cells and crucial function in enhancing tumor cell growth, survival, and drug resistance, it presents a hopeful objective for the development of immunotherapy. Recently, Bae et al. created and analyzed a nanovehicle-based formulation containing a heteroclitic BCMA peptide to enhance antigen delivery. This formulation induces stronger poly-functional BCMA-specific CD8^+^ cytotoxic T lymphocyte (CTL) responses against MM compared to vaccination with the peptide alone. The utilization of PLGA nanotechnology in this cancer vaccine improves the possible clinical use of BCMA in targeted immunotherapy for myeloma.^825^

### Diagnosis and treatment integration

The combination of targeting ligands, imaging markers and therapeutic drugs can achieve effectively controlled delivery in the body, which can perform non-invasive monitoring and treatment in real time. With the continuous exploration of multidisciplinary methods by scientific researchers, the use of nanotechnology will bring new directions for lymphoma therapy.

Bai et al. coupled biotinylated CD20 and CD3 antibodies to the surface of ultrafine Fe_3_O_4_ NPs modified with streptavidin to construct a bispecific nanoplatform (BSNP). Compared with clinical Gd-chelated contrast agents, synthetic BSNP with a hydrodynamic size of 30 nm had better MRI capabilities. In vitro, the nano-platform specifically targeted Raji cells expressing CD20 and improved the killing mediated by T cells. Furthermore, it had the potential to impede the growth of tumors and extend the lifespan of in vivo transplantation models of NHL. The modularly designed BSNP has great potential as a versatile nano-platform.^826^

Etrych et al. documented the efficacy against lymphoma and the potential for diagnosis of a recently developed near-infrared fluorescent dye, which includes polymer-DOX conjugates. The polymer DOX conjugate demonstrated substantial effectiveness against malignant lymphomas in murine models in comparison to the equivalent dosage of unconjugated DOX. Fluorine-labeled polymer prodrugs had good diagnostic potential. The fluorescence intensity of subcutaneous xenograft lymphoma was closely related to the change of lymphoma volume, so the treatment effect could be noninvasively evaluated.^827^

Currently, the advancement of nanotechnology has led to an increasing number of unconventional nanotechnology discoveries, offering promising possibilities for the diagnosis and treatment of lymphoma. Traditional death/viability analysis usually requires laborious sample preparation and expensive equipment or reagents. The study of Ninno et al. used electrochemical impedance spectroscopy as a label-free method to describe living, necrotic, and apoptotic human lymphoma U937 cells. At a low frequency of 0.5 MHz, the signal intensity was capable of differentiating between live/necrotic cells and cell debris, and the phase information could be used to distinguish live cells from necrotic cells. At a higher frequency (10 MHz), the cell fragments of the two subgroups were distinguished, suggesting the important role of electrical impedance technology in the next generation of cell viability testing.^828^ The tissue sample’s chemical atlas offers crucial data on biological processes. Over the past few years, there has been advancement in tissue imaging through desorption electrospray ionization mass spectrometry (DESI-MS). However, the capability of this technology in mapping lipids and metabolites is restricted. Hsu et al. documented the utilization of nano-DESI. Under atmospheric pressure, they only used a small amount of samples to visualize proteins in tissue samples without the need for labeling. Nano-DESI successfully detected multiple charged proteins with a mass of 15 kda. This imaging method can be further applied to MYC-induced lymphoma.^829^

When studying tumor proteins expression information, only the volume of the serum sample is large enough to be used for single target analysis of multiple protein analysis. However, most traditional mouse experiments cannot collect serum samples frequently. Lee et al. established a magneto-nanosensors to detect protein expression in serum. The difference of cytokine expression in plasma can be accurately measured by using this nanosensor. This nano-magnetic field sensor-based technology can be used to longitudinally monitor the protein profile of lymphoma model mice, facilitating multiple analyses which use only a small quantity of samples.^830^ To comprehend the diversity of cellular function and devise novel approaches for treating various ailments, it is imperative to conduct an examination of cytokine secretion at the single-cell level. Cell integrity is inevitably disrupted by the burdensome molecular labeling required in fluorescence and colorimetric-based methods. Li et al. discovered a groundbreaking optofluidic nanoplasmonic biosensor without the need for labels, enabling real-time examination of individual cells. The nanobiosensor utilized an innovative configuration of a versatile microfluidic system comprising of a compact microchamber and control channels to ensure accurate tracking of cytokine release from individual cells. Real-time determination of the secretion fingerprints of individual cells in space can be achieved through the utilization of optical spectral imaging in conjunction with sensor architecture. Anticipated to transform into a potent instrument for analyzing individual cellular signals in fundamental and medical investigations, this novel biosensor system holds great promise.^831^

Nanotechnology has broad prospects in the treatment of lymphoma. Nevertheless, the challenge of translating them into clinical practice persists, despite the significant advantages demonstrated by NP-based systems in preclinical environments. The development of effective nanomedicine formulations involves addressing various challenges, including the incorporation of drug molecules into NPs, ensuring the stability of NP formulations, and achieving controlled release of the encapsulated drugs at the desired location within the body. Furthermore, the absence of suitable animal models that replicate the real clinical scenario (etiology, pathology and progression) in lymphoma poses challenges in forecasting positive results for clinical trials. However, nanomedicines have been recognized and demonstrated as promising mechanisms for delivering drugs. Numerous formulations have been effectively developed and approved for the treatment of various malignancies as well as chronic inflammatory conditions. Further exploration in the field will result in innovative NP formulations for lymphoma treatment in the coming years.

## Nanomedicine in genitourinary system tumors

The genitourinary system is mainly composed of the urinary system and the reproductive system. The urinary system includes four parts: kidney, ureter, bladder and urethra, where the highest incidence of urinary system tumors is bladder cancer (BC), ranking ninth among all malignant tumors and sixth among male tumors. BC is characterized with rapid progression and high recurrence rate. There are approximately 549,000 cases diagnosed each year, and the incidence of women is always lower than that of men.^358,832–834^ Most BCs are urothelial cancers. Presently, around 75% patients suffer from non-muscle invasive bladder cancer (NMIBC), with 25% of them experiencing muscle invasive or metastatic illness.^835,836^ In 2016, the World Health Organization (WHO) “Blue Book” released the fourth edition of the WHO Classification of Tumors of the Genitourinary System, which suggested significant changes to male reproductive system tumors.^837^ Prostate cancer is the predominant ailment among tumors affecting the male reproductive system, while ovarian cancer and breast cancer exhibit the highest occurrence among tumors impacting the female reproductive system. Around 230,000 females worldwide receive an ovarian cancer diagnosis annually, with a mere 46% survival rate within 5 years after diagnosis.^838,839^ Additionally, breast cancer ranks as the second leading cause of cancer fatalities among women.^840^ Currently, the treatments of tumors of the genitourinary system are mainly surgery, chemotherapy, radiotherapy and others, but the existing treatments cannot achieve satisfactory therapeutic effects, and chemotherapy and radiotherapy often produce toxic side effects onto normal cells which is unsatisfactory and harmful to the body. Besides, tumors are highly metastatic which brings huge challenges to treatment. Hence, developing new diagnostic and therapeutic strategies is the key to solving these problems.

In recent years, the great advancement of nanotechnology has sparked optimism for the cure of malignant tumors. Many nanomaterials, such as inorganic NPs, polymer NPs, are designed for treating genitourinary system tumors and show great advantages.^731^ Presently, relying on nanomaterials as transporters for curative substances, external stimuli (light, magnetic waves, heat, etc) are introduced to expand their functions to realize drug release at tumor sites. While therapeutic compounds are often limited in clinical treatments due to poor aqueous solubility, ability to cross body barriers and penetrate into tumors, and low biological stability.^841^ A series of new treatment strategies were developed for various malignant tumors after combining nanotechnology with therapeutic compounds. Nanomaterials as a delivery system can not only prolong drug residence time and improve bioavailability, but also reduce or even avoid induced adverse reactions/drug toxicity caused by drugs. Next, we will focus on the application of nanomaterials as a delivery system in several common tumors of the genitourinary system.

### Diagnosis

The detection and diagnosis of tumors of genitourinary system is mainly through the detection of relevant biomarkers in the tumor and imaging methods. Early detection of tumors can be achieved by identifying biomarkers in the bloodstream, such as circulating tumor cells (CTC) or circulating tumor DNA (ctDNA), but most of the biomarkers are diluted after blood circulation, which leads to the difficulties with detection. Currently, imaging methods are widely used in the detection, location or staging of tumors. Commonly-used clinical imaging methods mainly include: X-ray,^842^ ultrasound,^355^ MRI,^843^ digital chest tomography,^844^ CT, PET, etc., which have the drawbacks of limited sensitivity and signal specificity, long acquisition time and the risk of ionizing radiation.^635,845^ Determining the boundary between the lesion site and normal tissue cells, accurately detecting metastasis, and accurately judging small tumors and occult tumors are still challenges for current tumor imaging technology. Therefore, using a visualization system to detect and kill tumor cells is a tumor treatment strategy with low side effects and high efficiency. Precise positioning and visualization are of great significance to its success rate and reduction of tumor mortality.^846^

The investigation of biomarkers for the prevention of early cancer and the mitigation of metastasis is a crucial undertaking and highly significant in clinical diagnosis and treatment. However, existing detection techniques demonstrate attributes of limited sensitivity, throughput, and affordability.^847^ Recently, microRNA (miRNA) has been reported as an attractive tool for liquid biopsy in tumor screening.^848^ The miRNA is an endogenously encoded non-coding RNA molecule consisting of around 22 nucleotides in length. It regulates protein-coding genes by specifically inhibiting translation or cutting ribonucleotide transcripts expression. There is growing evidence that miRNA is related to important tumor pathways.^849^ Therefore, miRNA can be used as a tumor marker to improve the efficiency and sensitivity of tumor detection. Wei et al.^847^ proposed a technique to detect miRNA associated with bladder cancer using photonic crystal (PhC) barcodes, which enables highly sensitive quantification of miRNA. The reflective peaks produced by periodic and organized porous nanostructures are distinctive features of PhC barcodes. Chemical coupling connects the probe to the barcode surface, while base pairing binds the miRNA. Simultaneously, the hybrid chain reaction (HCR) is utilized to amplify the detected signal. The findings indicate that the PhC barcode detection system exhibits excellent precision and correctness. Moreover, various categories of miRNAs can be measured simultaneously by attaching diverse probes onto the nanoparticles’ surface, enabling rapid, precise, and highly sensitive quantification of miRNA.

Exosomes are nanovesicles with a diameter of less than 200 nm, which fused with the plasma membrane in multivesicular endosomes (MVEs). Therefore, exosomes usually carry biological surface markers of specific membranes, such as surface proteins, which are receiving more and more attention in tumor detection and diagnosis. Moura et al.^850^ have devised an electrochemical immunosensor that utilizes a magnet to detect exosomes generated from three distinct breast cancer cell lines. This sensor has the capability to increase the sensitivity of detection and accurately differentiate between healthy individuals and those with breast cancer. Furthermore, research has demonstrated that the TME plays a crucial role in facilitating the release of exosomes, which are mostly concentrated in the hypoxic TME.^851,852^ An integrated magnetic-electrochemical exosome (iMEX) has been developed which was used to screen for ovarian cancer. Exosomes are captured from patients through immune-magnetics and analyzed through electrochemical reactions. Magnetic enrichment and enzyme amplification realize high-sensitive and specific exosome detection, as well as the miniaturization of sensors and the amplification of high-throughput measurement, which further improves the ability of rapid, high-throughput and on-site analysis.

Imaging technology cannot accurately detect and visualize cancer staging, however, the application of nanomaterials to tumor imaging can increase its sensitivity and targeting, reduce adverse reactions, improve imaging effects and achieve visualization.^853^ Yang et al.^854^ developed a therapeutic nano-drug (AuNCs-Pt) with both near-infrared-I/II (NIR-I/II) imaging and glutathione scavenging functions based on gold nanoclusters (AuNC). The AuNCs-Pt possesses the ability to perform NIR-II imaging in the model of abdominal lethal high-grade serous ovarian cancer (HGSOC), enhancing the visualization of platinum transportation in deep tissues. Consequently, it is anticipated to emerge as a valuable instrument for investigating the in vivo drug destiny. Meanwhile, AuNCs-Pt can also deplete glutathione in cells which minimizes the detoxification effect to platinum-based drugs, and maximize the chemotherapy effect. Recently, some studies have found that fluorescence and multispectral photoacoustic tomography (MSOT) imaging can detect local or distant breast cancer metastases caused by tumors in situ.^855^ Some researchers have developed an activatable nanoprobe with aggregation-induced emission capability, which can detect and image breast cancer metastasis in mouse models through NIR-I and NIR-II fluorescence imaging^856^ and MSOT. The nanoprobe is capable of detecting and reacting to an increased presence of nitroreductase in tumor hypoxia. It produces distinct NIR-I/NIR-II signals and photoacoustic signals, allowing for a comprehensive visualization of breast cancer metastasis. In addition, Zhao et al.^846^ also proposed a new gene amplification strategy in nanoparticle tumor homing, and designed a prostate cancer cell-specific genetic probe (PDD3-TfR-WPRE-PCMV-Luc) to promote high expression of transferrin in cells. Then transferrin (Tf) modified by ultra-small iron oxide superparamagnetic nanoprobes (Tf-USPins) is used for detection. The results show that the contrast of MRI imaging compared with surrounding normal tissues is enhanced, which helps to be more accurate positioning. Additionally, Li et al. developed the polyethylene glycol-manganese dioxide NPs as a T1MRI contrast agent in renal cancer.^857^

Typically, penile cancer is assessed by conducting a sentinel lymph (SN) node biopsy with the aid of 99mTc nano-colloid and blue dye. To improve the optical detection sensitivity, a combination of a dual-labeled tracer, comprising the fluorescent dye indocyanine green (ICG) and 99mTc nano-colloid (ICG-99mTc-nano-colloid), was used. The results showed that fluorescence imaging of the NPs improved the detection of optical SN without contaminating the surgical area or additional injections, improving the accuracy of detection of penile cancer preoperatively and intraoperatively.^858^

Squamous-cell carcinoma antigen (SCC-Ag) is a biomarker found in the blood that is highly expressed in gynecological tumors of squamous-cell carcinoma. A recent study has demonstrated that the utilization of nanogold-labeled biomolecules can enhance the precision of detection. The sensitivity of SCC-Ag-GNP on SCC-Ag-antibody is greater, indicating that this method using a gold-labeled probe or target can assist in the identification and quantification of gynecological tumors, thereby assessing their severity.^859^

### Treatment

At present, surgical treatment, radiotherapy and chemotherapy are still the first choice for cancer of urogenital system. Because of its poor prognosis and easy to produce drug resistance, which affects patients’ mental health and happiness, it is imperative to develop new treatment strategies to alleviate patients’ pain and prolong their survival time. Currently, great progress has been made in the research of nano-drugs in prostate cancer, breast cancer and other high-incidence malignant urogenital tumors, including diagnosis, gene therapy, immunotherapy and targeted therapy.

#### Chemotherapy

Chemotherapy plays an important role in oncotherapy, but traditional chemotherapy normally lacks specificity. Besides, the concentration of drugs in tumors is always not high enough, so large doses of drugs are often required which can produce toxic side effects onto normal cells. Paclitaxel, as a clinical first-line anti-tumor drug, is widely used in treating breast cancer, prostate cancer and other malignant tumors, but its shortcomings such as poor water solubility and high toxicity greatly limit its applications. The emergence of nanotechnology provides a new treatment strategy. Using nanomaterials to load chemotherapeutic drugs can prolong the circulation of drugs in the body, increase the bioavailability of the drugs and reduce toxic side effects, etc., so as to enhance the therapeutic effect of chemotherapy. Guo et al.^860^ developed a super-thermodynamically stable RNA 4WJ-X nanoparticle based on the PRNA-3WJ motif, which was used for solubilizing and loading paclitaxel for targeted cancer treatment. RNA-paclitaxel complex nanoparticles are chemically stable, and their water solubility is increased by as high as 32,000 times. The utilization of distinct ultra-heat resistant RNA nanoscale particles for the purpose of delivering paclitaxel intends to address the issue of RNA unwinding and nanoparticle separation during the loading of drugs at high density. According to the research findings, the administration of RNA-paclitaxel NPs through intravenous injection, along with ligands that specifically target breast cancer, effectively suppressed tumor growth in the mice model. Furthermore, almost no toxicity and immune responses were observed during the study.

In chemotherapy, in addition to the side effects caused by chemical drugs, the development of drug resistance is also one of the important reasons for the poor effect of tumor treatment. It has been found that prostate cancer is prone to develop drug resistance during chemotherapy,^861^ and it has been proven that the development of drug resistance is caused by the aging mechanism of prostate cancer in preclinical and clinical studies.^862^ Tannins was reported to show anti-tumor effects in prostate cancer and can prevent cancer cells from developing drug resistance.^863^ However, the two drugs are not so useful when administrated separately due to drug resistance, dosage, etc. Therefore, a tannins-docetaxel self-assembly (DSA) nano-formulation was developed for this phenomenon.^863^ The experimental results show that DSA shows superior tumor targeting, anti-cancer activity and inhibition of senescence blocking and the removal of the senescent cells in the tumor, which promotes the efficacy of docetaxel.

Chemotherapy, surgery, radiation therapy and targeted therapy are still the most commonly used treatments for cervical cancer. Cisplatin, one of the commonly employed treatments in radiotherapy and chemotherapy regimens for cervical cancer, is associated with significant systemic side effects and vulnerability to drug resistance. This event is strongly correlated with the rise in sulfur-containing compounds within the cell, particularly glutathione (GSH), as well as the increase of the glutathione S-conjugate pump, which relies on adenosine triphosphate (ATP). Luo et al.^864^ utilized the nano-precipitation method to create the Pt (IV) prodrug nano-drug SSCV5NPs, which consists of a VES-modified prodrug and a VES-modified disulfide-rich carrier. The nano-sensitizer possesses the attributes of safety, redox sensitivity, a substantial disulfide density, and a high drug loading capacity (reaching up to 16.50%Pt or perhaps even more).

#### Radiotherapy

Since Roentgen discovered X-rays in 1895 to the first clinical application of radiotherapy in 1896, radiotherapy (RT) has undergone tremendous development during the years. Until now, radiotherapy is still one of the indispensable treatments for tumor treatment. However, it is still a challenge to deliver appropriate radiation doses to the tumor site to reduce damage to normal tissues in RT. Currently, there are reports of using radiosensitizers to reduce the side effects of RT.^865^ In addition, MRI-guided RT can further improve the accuracy and effectiveness of treatment. A gold-gadolinium (III) NPs (Au-Gd (III)-PSMA) targeting PSMA (highly expressed in prostate cancer) was synthesized to selectively deliver gadolinium (III) contrast agents to prostate cancer.^866^ The results indicated that the gold-gadolinium (III) PSMA NPs showed favorable tumor accumulation, and enhanced the cell’s magnetic resonance contrast and radio-sensitization, meanwhile they also reduced the damage to the surrounding normal tissues. In addition, the biological characteristics of the tumor will also have an important impact on the efficacy of radiotherapy. Insufficient oxygen supply has been proved to be one of the main drivers of tumor angiogenesis and metastasis. However, traditional low-dose radiotherapy may aggravate hypoxic conditions and impels the tumor cells less susceptible to radiation-induced killing.^867^ In order to solve the challenges, Huo et al.^868^ developed a nanocluster “bomb”. This “bomb” is used as a radiosensitizer for RT, and is composed of non-stoichiometric tungsten oxide nanoparticles (WO NP) to target a low-oxygen microenvironment and modified with a ligand of CCL-28 chemokine. The majority of these NPs maintain their original bigger size while circulating in the body, resulting in a prolonged half-life and passive targeting of tumors through EPR effects. When reaching the tumor site, the original NPs will be converted into small NPs under the stimulation of the TME (such as enzymes, pH) to promote the deeper penetration into the tumor. Meanwhile, it enables higher resolution nanoparticle-enhanced CT to achieve accurate imaging of the hypoxic contour of solid tumors. Local photothermal ablation of the tumor and the generation of free radicals can be achieved at the same time under 1064 nm laser irradiation and CT guidance. The induced free radicals reduce the resistance induced by hypoxia in an Akt-mTOR pathway-dependent manner, and enhance the tumor’s radiation killing sensitivity to action.

#### Gene therapy

Non-coding RNA (ncRNA) is becoming an important regulator in the process of various diseases, including circular RNA, lncRNA, siRNA, etc. Abnormal expression of non-coding RNA is associated with tumor occurrence; therefore, it attracts increasingly more researchers’ attention.^869–872^ The role of siRNA in cancer has been well studied. siRNA is a double-stranded ribonucleic acid molecule^873^ which is a powerful tool for gene silencing. It has an inhibitory effect on the expression of target genes and can target almost any gene,^874,875^ so it has the potential to treat various diseases. Due to the poor stability of naked RNA in the blood, it is often degraded in endosomes/lysosomes, and negatively charged genes cannot pass through the plasma membrane.^876^ Protecting siRNA from nucleases to reach cancer sites in deep tissues, and avoiding activating immune responses, etc. are challenges in the clinical application.^877^ In addition, the choice of vector also has an important impact on the efficacy of gene therapy. The ideal gene vector should be highly effective, specific and biocompatible. Viral vectors frequently used in the past have shown high efficiency in delivering genetic material, but the application is greatly limited due to the packaging capacity, instability and immunogenicity of the vector.^878^

In recent times, there has been a surge in the advancement of nanotechnology, leading to the emergence of novel approaches for delivering siRNA. Various materials (lipids, polymer micelles, etc.) have the purpose of acting as transporters for delivering siRNA, thereby prolonging the lifespan of RNA in the bloodstream. These carriers have a preference for accumulating in tumors, improving cellular uptake, and successfully escaping endo/lysosomes. Dong et al. developed the siRNA-loaded amphiphilic phospholipid dendrimers (AmPPDs).^879^ They found that NPs protected siRNA from enzymatic degradation and promoted the uptake and release in prostate cancer. Besides, halloysite nanotubes (HNTs) are also commonly used as non-viral vectors to capture and release active agents. HNTs were used to deliver therapeutic siRNA to target RIPK4 (receptor interacting protein kinase 4) which are highly expressed in bladder cancer cells.^880^ Studies have found that NPs coated with HNTs are more stable in the body, easily taken up by cells and accumulated in the bladder cancer lesion, thereby effectively inhibiting the occurrence and development of bladder cancer.

Although many nano-delivery systems have been developed for gene therapy, such as polymer NPs, liposomes, inorganic nanoparticles, genes encapsulated in an acidic environment rich in enzymes are easily degraded and inactivated. Meanwhile, the release of drugs is uncontrolled and inaccurate. These have greatly affected the therapeutic effect of gene therapy. Therefore, in the small nucleic acid nanomedicine therapy, another challenge is that most of the small nucleic acid-based drugs are rapidly transported to the lysosome, resulting in the degradation. Van de Vyver et al.^881^ found that cationic amphiphilic drugs (CADs) can transiently induce the lysosomal membrane permeability and strongly promote the delivery of functional siRNA from lysosomal compartments.

The TME plays a crucial role in the development, proliferation, and spread of malignancies. Cancer-associated fibroblasts (CAF) are known to have a significant impact on the TME and various other processes, including extracellular matrix (ECM) remodeling, facilitation of local invasion, and promotion of metastasis.^882^ C-X-X motif chemokine 12 (CXCL12) ligand participates in the migration of various cancer cells,^883^ and activates multiple responses (such as migration, proliferation, etc.^884^) through the CXCL12/CXCR4 pathway in the tumor microenvironment. Therefore, a group developed a nano-system targeting CAF and CXCL12 to inhibit the metastasis of prostate cancer. They used cell-penetrating peptide (CPP)-based self-assembled nanoparticles to load siRNA that can silence the CXCL12 gene, and modify the NPs with fibroblast activating protein-α monoclonal antibody to target CAF to specifically deliver siRNA to cells. The findings demonstrated a considerable inhibition of tumor cell migration, invasion, and tumor angiogenesis.^885^ The microenvironment of malignant tumors is characterized with low oxygen and slight acidity. Xu et al.^312^ designed a low-pH activated “nano-bomb” based on the acidic environment of tumor microenvironment to deliver POLR2A siRNA (SiPol2) and precisely target POLR2A in TP53^loss^TNBC. This nano-delivery system reduces the growth of POLR2A^loss^ tumors by inhibiting POLR2A. Meanwhile there is no obvious systemic toxicity.

#### Immunotherapy

Cancer immunotherapy aims to promote the immune system to recognize and eliminate cancer cells. A series of local cancer immunotherapies, such as in situ vaccines and oncolytic viruses, can help reverse the immunosuppression in TME and lead to a systemic anti-tumor response, which is regarded as a particularly promising intervention.^886^ In prostate treatment, immunotherapy has also shown great success. For example, therapeutic anti-cancer vaccines, including dendritic cell (DC) (Provenge), whole cell (GVAX) and carrier (PROSTVAC)-based vaccines, are the main immunotherapy strategy used to treat prostate cancer.^887,888^ However, issues such as targeting and off-targeting in immunotherapy have received widespread attention. The application of NPs holds great potential in addressing the issue of targeted distribution and mitigating off-target negative effects to a certain degree. Additionally, it has the potential to boost the stability, pharmacokinetics, and tumor accumulation, hence improving the effectiveness of anti-tumor actions and reducing systemic side effects in checkpoint blocking therapy. Nanocarrier vaccines have been reported to exhibit excellent stability and biocompatibility, and increase the accumulation of antigens/adjuvants in lymph nodes (LNs). Meanwhile, they can be delivered to DC in a controlled release manner, which may improve cytotoxic T lymphocyte (CTL) and humoral response. In addition, they can prolong the antigen presentation process, and significantly improve the anti-tumor immune response.^889^ Virus-like nanoparticles (VLP) have become an attractive means of tumor immunotherapy due to their unique innate immune stimulating properties. Wang et al.^301^ developed a bacteriophage QβVLPs-loaded Mg-based micromotor. It can be propelled autonomously in the peritoneal fluid so that complete immunostimulatory QβVLPs can be actively delivered to the abdominal cavity of mice with ovarian tumors. It greatly improves the local distribution and retention of QβVLPs, enhances immune stimulation, and improves the survival rate of tumor-bearing mice.

#### Targeted therapy

NPs can selectively target to tumors through appropriate surface modification. NPs are used as delivery vehicles, which can passively accumulate into the tumors through enhanced penetration retention effect. In addition, active targeting, using NPs modified with specific ligands for disease biomarkers, increases the affinity of NPs for target tissues.^890,891^ Prostate-specific membrane antigen (PSMA) is a type II transmembrane protein composed of 170 amino acids. It is overexpressed in the new blood vessels of prostate cancer and other solid tumors (such as breast cancer). It has been identified to distinguish between benign and malignant tumors.^892^ Joey et al.^890^ constructed prostate-specific membrane-targeted gold NPs. The PSMA-1 ligand underwent conjugation with polyethylene glycol terminated with sulfhydryl groups. Subsequently, it was connected to the surface of gold nanoparticles that contained the fluorescent photodynamic therapy drug Pc4. This connection was achieved through a ligand exchange reaction. Experiments conducted in vivo and in vitro suggest that nanoparticles have the ability to specifically transport medications, while photodynamic therapy can interfere with the formation and growth of tumors. Approximately 20–30% of breast cancers exhibit an overexpression of human epidermal growth factor receptor-2 (HER2). HER2 belongs to the EGFR family and has a weight of 185 kDa. In the treatment of HER2-positive breast cancer, blocking HER2 signaling pathway is a successful approach.^893,894^ A new strategy is to use HER2 *N*-glycan nanomolecular imprinted polymer (nano MIP) to effectively adhere to almost all HER2 polysaccharides. This attachment prevents the dimerization of HER2 with other members of the HER2 family, ultimately obstructing downstream signaling pathways and reducing cell proliferation by 30%. Consequently, this method successfully achieves the objective of inhibiting HER2-positive breast cancer.^894^ The drug can be injected directly into the bladder through a catheter in the treatment of bladder cancer. Although the diseased tissue is directly exposed to drugs to increase the therapeutic effect and reduce systemic side effects, the efficacy of drugs may be affected due to the bladder wall glycosaminoglycan (GAG) mucosa and periodic urination and other factors.^895^ Currently, micro-nanomotors or nano-nanomotors have great potential in drug delivery and have received great attention with the development of nanotechnology.^896^ Hortelão et al.^897^ designed an antibody-modified urease-driven nanomotor based on the over-expressed fibroblast factor 3 (FGFR3) in bladder cancer. Urea provides propulsion for nanomotors under the action of urease biocatalysis. The results show that the antigen-antibody interaction and targeting make the nanomotor composed of anti-FGFR3 show more effective internalization and the block of fibroblast growth factor (FGF) pathways which prevent cell proliferation and lead to cell death.

#### Combined therapy

As mentioned above, immunotherapy has played a favorable therapeutic effect in the treatment of prostate cancer, but due to the existence of immunosuppression in the TME, the application of therapeutic cancer vaccines is subject to certain restrictions. TME not only plays an important role in the progression of cancer, but also is a major obstacle to cancer immunotherapy. Regulatory T cells (Treg), and immune checkpoints, such as CTL associated protein (CTLA-4), PD-1, etc., play important roles in the immune suppression.^898,899^ Therefore, overcoming tumor microenvironment is the key to successful cancer immunotherapy. It is reported that standard cancer treatment methods such as chemotherapy and radiotherapy can induce immunogenic stimulation or death of tumor cells and produce a large amount of tumor antigens.^900^ Hence, the implementation of combined immunotherapy is an excellent approach for treatment. A novel formulation called Fer-ICB-UPMSNPs, consisting of Ferumoxytol (Fer) and anti-programmed cell death-ligand 1 antibodies, loaded onto ultralarge pore mesoporous silica nanoparticles, was created. This formulation aims to provide sequential MRI-guided local immunotherapy following cabazitaxel (Cbz) chemotherapy, specifically for the treatment of prostate cancer and other tumors of the urogenital system.^901^ The findings indicated that the T cell infiltration is effectively activated by the local delivery of Fer-ICB-UPSnPs guided by MRI following Cbz treatment. Meanwhile, it also reduces the role of Treg in the tumor-specific adoptive immune response and significantly inhibits the tumor growth.

A combination therapy consisting of gene therapy and chemotherapy is an effective method to treat tumors. It was reported that a photo-activated Pt (IV) prodrug backbone polymer nanoparticle system (CNP_PtCP/si(c-fos)_) could deliver small interfering RNA (si(c-fos)) in a light-controlled manner. This system was employed in conjunction with light-activated chemotherapy and RNA interference (RNAi) to treat platinum-resistant ovarian cancer.^902^ CNP_PtCP/si(c-fos)_ can produce oxygen-independent N_3_• with slight oxidation energy under blue light (430 nm) irradiation. The N_3_• assisted photochemical internalization (PCI) mechanism can effectively escape the action of lysosomes or endonucleases and reduce the inactivation of gene c-fos. Subsequently, while the Pt (IV) prodrug is activated to be Pt (II), CNP_PtCP/si(c-fos)_ is dissociated, and the active Pt (II) and si(c-fos) are released. Both in vitro and in vivo experiments have shown that CNP_PtCP/si (c-fos)_ has an outstanding synergistic therapeutic effect on platinum-resistant ovarian cancer. This system can further improve the effect of gene therapy even in the hypoxic TME.

## Nanomedicine in primary bone tumors

Neoplasms originating from bones are exceptionally uncommon, constituting less than 0.2% of all cancer cases. However, accurately determining their incidence is challenging due to the infrequency of these tumors.^903^ In 2019, approximately 3500 people were diagnosed with this disease, resulting in around 1660 deaths in the United States.^3^ Primary bone tumors exhibit diverse clinical variability and can frequently be cured with appropriate therapy. Bone cancers are classified according to their histologic origin. The three most prevalent types of bone cancer are osteosarcoma (35%), chondrosarcoma (30%), and Ewing sarcoma (16%). Chondrosarcoma originates from cartilage, osteosarcoma originates from bone, fibrosarcoma of bone originates from fibrogenic tissue, hemangioendothelioma and hemangiopericytoma originate from vascular tissue, and chordoma arises from notochordal tissue. The histologic origin of several primary bone cancers, such as Ewing sarcoma, remains unknown.

The prognosis for patients with osteosarcoma and Ewing sarcoma has significantly improved due to the development of multiagent chemotherapy regimens for neoadjuvant and adjuvant treatment.^904–906^ As a result of current multimodality treatment approaches, approximately three quarters of all osteosarcoma patients are cured, and 90% to 95% of osteosarcoma patients are successfully treated with limb-sparing approaches rather than amputation. In some cases, patients diagnosed with metastatic disease at presentation can still achieve a cure, especially among those with Ewing sarcoma and osteosarcoma.^907^ Among current multimodality treatment, nanotechnology is widely recognized as a promising drug delivery platform. This section will provide an overview of the most recent findings and advancements in nanotechnology for detecting and treating primary bone cancer.

### Diagnosis

Osteosarcoma is the prevailing primary cancer of bone tissue, disproportionately impacting children and teenagers compared to other age groups. This is the most frequently detected primary bone cancer, with a yearly occurrence of 0.2–3 cases per 100,000 in children and 0.8–11 cases per 100,000 in adolescents. The highest occurrence is observed during the second ten years of life. Although only 20% of individuals show clinically detectable metastasis, it is presumed that the majority of the remaining 80% have undetectable micro-metastases upon diagnosis.^908^ Cancer may be detected on the surface of the bone, inside the bone, or in locations outside the bone, such as the lung.^909–911^ The malignant bone tumor is identified by the creation of immature osteoid extracellular matrix and originates from mesenchymal cells.^318,910,912^ Nanotechnology-based approaches are increasingly utilized in the detection and therapy of osteosarcoma to enhance its early identification.

When a patient experiences pain, plain radiographs are frequently used to initially identify osteosarcoma. Typically, MRI with contrast enhancement is the subsequent stage in the diagnostic evaluation, and the images should encompass both the entire affected bone and the neighboring bones. The histopathological identification of osteosarcoma relies primarily on observations in specimens stained with hematoxylin and eosin, despite the wide range of morphological variations seen in osteosarcoma. In addition to visualizing the main tumor, it is crucial to conduct a chest CT scan during the initial stage to determine the extent of metastasis to the lungs, as this is the most common site for osteosarcoma metastasis. Typically, it is advisable to undergo either a bone scan or a PET scan to assess the presence of distant bone metastases.^913,914^ Nevertheless, it has been revealed that the utilization of nanotechnology in the detection of osteosarcoma has made minimal advancements, although there are still numerous groundbreaking investigations. Meng et al.^915^ proposed a novel approach involving negative CT contrast agents (NCTCAs) in order to decrease the CT-DV associated with osteosarcoma. Clinical iodides, which are the existing positive CT contrast agents (PCTCAs), exhibit a significant CT density value (CT-DV). Nevertheless, they lack the ability to provide precise diagnoses for certain diseases that have a high CT-DV, like osteosarcoma. Since bones and PCTCAs in osteosarcoma typically produce comparable levels of X-ray attenuations. Simultaneously, they fabricated empty mesoporous silica nanoparticles, incorporating ammonia borane molecules and subsequently modifying them with polyethylene glycol. These nanoparticles have been synthesized as NCTCAs for the detection of osteosarcoma. By reacting to the acidic microenvironment of osteosarcoma, the nanocomposites have the ability to generate H_2_ directly at osteosarcoma sites, leading to an approximate 20-fold decrease in CT density within osteosarcoma regions. By creating a significant difference in CT density between bones and osteosarcoma, this method effectively enables precise detection and diagnosis of osteosarcoma. In addition to identifying primary osteosarcoma, it is crucial to use imaging techniques to detect lymph node metastasis in bone tumors, as it serves as a significant factor in assessing patients’ prognosis. Accurately imaging the lymph nodes is crucial for tumor staging and surgical planning, such as in the case of osteosarcoma. From a clinical perspective, the use of a radio-nanocolloid for nodal tracing is frequently restricted due to the unavailability of live visuals and insufficient anatomical data. The study conducted by Xu et al.^916^ showed that the (99m)Tc-biomineralization nanoprobe has the potential to be utilized in the precise and sensitive examination of lymphatic drainage in a model of orthotopic osteosarcoma.

Early diagnosis and treatment of osteosarcoma can be bridged with the assistance of nanotechnology, which can enhance the ratio of successful cures for the disease. A novel variant of mesoporous silica-coated bismuth sulfide nanoparticles (Bi2S3@MSN NPs) was developed by Lu et al.^917^ Upon being covalently linked to the arginine-glycine-aspartic acid (RGD) peptide [c(RGDyC)], the NPs demonstrated remarkable selectivity towards osteosarcoma. Because of this, their accumulation in tumor cells is ten times higher than that in the surrounding peritumoral tissues. This makes CT imaging and tumor ablation possible.

### Treatment

#### Chemotherapy

Nanomedicine has become a prominent field in biomedical research technology, supporting the detection, treatment, and control of various diseases. Nanocarriers have the ability to effectively incorporate drugs and various molecules for precise treatment. To facilitate the therapy, various nanosystems have been created.^918–923^ In current researches, nanotechnology-based approaches for cancer therapy are highly relevant as they aim to improve targeting efficiency, minimize systemic adverse effects, and lower drug dosage. In the realm of cancer treatment, various methods have been explored, including diverse pharmacological and mechanistic techniques.

Significant advancements have been made in the field of novel drug delivery systems, and the utilization of nanotechnology in biomedicine has emerged as a highly advantageous method. This method greatly improves the drugs’ availability (by making them more stable and dissolvable) and their distribution throughout the body. It also allows for controlled release, longer circulation, targeted delivery to specific sites, and higher levels of accumulation in tumors. As a result, it achieves a selective targeting delivery.^924–926^ Numerous studies have demonstrated that DOX, a traditional medication for osteosarcoma, can enhance the cytotoxicity to osteosarcoma cells when utilized in conjunction with various drug delivery systems.^917,927–941^ These systems include hyaluronic acid (HA) nanogel, polymeric NPs, exosomes, mesoporous silica NPs, Fe_3_O_4_ NPs, calcium carbonate (CaCO_3_)-core-crosslinked NPs, liposomes, and platinum NPs (PtNPs). The use of these nanoparticles has been instrumental in addressing the challenges of drug resistance and metastasis. They have effectively enhanced the accumulation of drugs in solid tumors while minimizing the adverse effects of chemotherapy medications. Though the specific mechanisms and pathways of these drug delivery platforms to achieve targeted therapy are not completely the same, their effects on traditional chemotherapy drugs are similar and obvious.

In addition to DOX, there are other drugs that can be encapsulated in the NPs, such as berberine, methomyl, paclitaxel, curcumin and other chemotherapeutic drugs. Utilizing nanotechnology in conjunction with these medications can significantly enhance drug effectiveness while minimizing adverse reactions. For instance, Hsu et al.^942^ created a new formulation of berberine NPs using heparin (HP) and BBR, with or without a shell made of linear polyethyleneimine (LPEI) to improve its effectiveness in inhibiting tumor growth in osteosarcoma U-2 OS cells. Li et al.^943^ investigated the use of poloxamer-modified trimethyl chitosan (TMC) encapsulated MTX to enhance the effectiveness of osteosarcoma treatment and reduce the harmful side effects linked to the clinical application of MTX. The researchers discovered that MTCN displayed higher toxicity in MG63 cells when compared to free MTX, primarily because of its improved cellular absorption. Martella et al.^944^ provided a comprehensive analysis of photo- and chemo-active keratin NPs that were loaded with two different substances. These NPs were proposed as an innovative method for delivering drugs to treat osteosarcoma, both in vitro and in a chemical context. The high molecular weight and water-soluble keratin is used to prepare the NPs, which are then functionalized with the photosensitizer Chlorin-e6 (Ce6) and loaded with the chemotherapeutic drug Paclitaxel. Li et al. exploited an effective drug targeting osteosarcoma.^945^ This research examined the use of a peptide-decorated nanogel made from disulfide-crosslinked polypeptides (STP-NG) to improve the intracellular delivery of shikonin (SHK), a medicinal herb extract. The goal is to hinder the progression of osteosarcoma while minimizing any harmful effects on the body. In vitro, the STP-NG/SHK nanogel, which was specifically designed and filled with substances, effectively eliminated osteosarcoma cells by triggering necroptosis that relied on the presence of RIP1 and RIP3.

Combining NPs can also serve as a carrier for various chemotherapy medications to enhance their effectiveness against tumors. In the study conducted by Liu et al.^946^, they successfully developed and examined the therapeutic effectiveness of glycol chitosan nanoparticles loaded with both gambogic acid (GA) and retinoic acid chlorochalcone (RACC) against osteosarcoma. The glycol chitosan nanoparticles (RGNP) loaded with GA/RACC were of nanoscale size and demonstrated a controlled drug release at both pH 7.4 and pH 5.0. Due to the robust positive charge on the surface of RGNP, cancer cells exhibited efficient cellular uptake. Li et al.^933^ synthesized a copolymer called mPEG-PαLA by attaching polyethylene glycol (PEG) to poly(α-lipoic acid). This copolymer was designed to act as a nanocarrier that responds to changes in both reduction and pH levels. It was used to carry both paclitaxel and DOX concurrently for the treatment of osteosarcoma. The NP-PTX-DOX sample exhibited an increased release of PTX and DOX when exposed to reducing and acidic conditions.

#### Other therapy

**Fig. 5:**
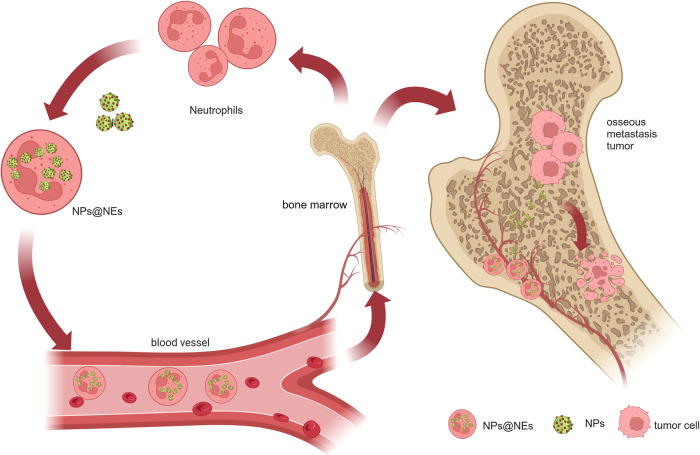
Bone marrow-derived neutrophils ingest nanopharmaceuticals containing CTX, transforming into NPs@NEs. These cells return to the bone marrow, where they exert a therapeutic effect on senescent bone metastatic tumors. (Created with BioRender.com) Reproduced with permission from Luo et al. (2023), Copyright 2023 Springer Nature Limited

In addition to chemotherapy, other treatment methods can also apply nanotechnology to deliver the drugs, such as genetic therapy, radiotherapy, immunotherapy and so on.^947–951^ The efficacy of pharmacological therapy is hindered by the limited penetration of drugs into the bone marrow, mostly attributed to the existence of the bone marrow-blood barrier. Luo et al. presented their findings on the utilization of neutrophils as a means of delivering free medicines and drug NPs specifically to the bone marrow. The study demonstrated the absorption of drug-loaded poly (lactic-co-glycolic acid) NPs by neutrophils and their subsequent transfer through the bone marrow-blood barrier, leading to increased drug levels in the bone marrow. The findings of this study demonstrated that the administration of cabazitaxel by neutrophil transport in a bone metastasis cancer model led to a substantial suppression of tumor growth (Fig. 5).^952^ Haghiralsadat et al. reported that siRNA, which will not be hardly cleared in the blood, can successfully reach the tumor cells and be released in the acidic environment.^936^ Similar results were obtained in the research by Wang et al.^953^ As for immunotherapy, it has been reported that all-trans retinoic acid (ATRA), a metabolically active form of vitamin A belonging to the retinoid family, will not change the structure and biological characteristics of the drug, but can promote the drug act on the target rapidly and efficiently by using nanomaterials-based carriers.^954,955^ Zhang et al. created therapeutic gadolinium-based metal-bisphosphonate NPs dubbed OVA-GdZol NPs that were stabilized by ovalbumin (OVA) and worked as both radiosensitizers and immune adjuvants for osteosarcoma treatment.^956^

Cartilage malignancies occur in various parts of the body and tend to be more prevalent as individuals get older. Conventional chondrosarcoma may typically be cured with surgery, particularly when it is at a low histologic grade. However, alternative treatments are pursued when the tumor cannot be removed surgically, which usually happens after several local recurrences or the formation of pulmonary metastases.^957^ The palliative use of cisplatin and DOX can sometimes provide therapeutic benefit for those with advanced disease and a favorable performance status. There is few research progress in nanotherapy of chondrosarcoma. According to a publication, chondrosarcoma cells were exposed to an epithelial neuron beam in vitro after the delivery of b-MSNs and exposure to BNCT (boron-neutron capture treatment) beam. This exposure effectively caused DNA damage and subsequent cell death.^958^

Ewing sarcoma is a malignant tumor that primarily develops in the bones (especially in the femur, tibia, ribs and pelvis) or soft tissues (particularly in the cervical muscles, thoracic wall, pleural cavities, and gluteal muscle). It mainly affects children, adolescents, and young adults, with approximately 1.5 cases per million individuals in this age group worldwide.^959^ Patients having a new diagnosis of Ewing sarcoma are treated with a combination of local control measures (radiation therapy and/or surgery) and multi-agent cytotoxic chemotherapy.^960^ Induction chemotherapy is administered prior to local treatment in order to diminish the size of the primary malignancy and inhibit micro-metastatic lesions, as it is anticipated to be present in all patients.^961^ Although the nanotherapy for Ewing’s sarcoma has not been extensively studied, it has been proven that the targeted administration and controlled release of SN-38 from gold NPs in Ewing sarcoma cells is highly effective both in in vitro and in vivo studies.^962^ Besides, hydrolyzed galactomannan (hGM)-based amphiphilic NPs also showed favorable performance of rapid accumulation in the tumor tissue.^963^ Additionally, due to Ewing sarcoma being caused by a fusion oncogene, such as EWSYFli1, a siRNA molecule could be used to reduce its expression. Toub et al.^964^ utilized noncationic polyisobutylcyanoacrylate nanocapsules as a safeguard in vivo delivery mechanism for siRNA molecules targeting the Ewing sarcoma oncogene. This method was significantly more effective at inhibiting tumor growth compared to using free siRNA.

## Nanomedicine in skin cancers

Malignant melanoma (MM) is the most serious form among skin cancers. This is the fifth most prevalent cancer among both men and women in the United States.^965^ Cutaneous squamous cell carcinoma (cSCC) is the second most frequent nonmelanoma skin cancers (NMSCs) after basal cell carcinoma (BCC).^966^ The cSCC and BCC account for 99% of NMSCs.^965,967,968^ According to the up-to-date clinical guideline,^969,970^ the most widely-used radical cure for skin cancers today is still surgery. The other mainstream treatments include cryotherapy, photodynamic therapy, immunotherapy and chemotherapy. However, traditional non-surgery treatments for skin cancers have some troubles in the precise positioning and infiltration through the barrier of skin. Thus, the NP is promising in the application of any kinds of therapeutic tactics except of surgery to overcome the disadvantage of traditional treatments. As for the diagnosis of skin cancer, most of MM and NMSCs depend on dermoscopy and biopsy, which are relatively more available and accurate than other cancer types because of the location on the surface.^971–974^ In that case, since the dermoscopy and biopsy can meet the demand of diagnosis of skin cancer, limited researches are dedicated in exploring the potential of NP in the area of diagnosis.

### Diagnosis

Unlike NMSCs, since there is no single pathological feature to diagnose melanoma, histopathological diagnosis is based on a combination of structural, cytological, and host reaction characteristics. Immunohistochemistry, helpful in distinguishing the melanocytic origin of difficult lesions, and other types of molecules methods are widely applied in the diagnosis of MM,^975^ which meet the common requirement with both sensitivity and specificity of above 90%.^976^ According to Wang et al., the H_2_O_2_-responsive photoacoustic (PA) imaging modality can also be used in differential diagnosis by different enhanced tumor signals among different skin cancers. In addition, it is recognized that the increasing level of hydrogen peroxide is relevant to the proliferation of tumor cells. Under this hypothesis, Wang et al. managed to develop a probe named intelligent H2O2-responsive ABTS-loaded HRP@Gd nanoprobes (iHRANPs) to determine the position of cancer tissue and distinguish cSCC from malignant melanoma.^977^

### Treatment

Compared to NMSCs, malignant melanoma is more aggressive and has more opportunity to metastasize, which means it is relatively difficult to treat and leads to a worse prognosis.^978–982^ As for the malignant melanoma with a limit level of metastasis, surgery can always make a good prognosis.^978^ Therefore, the greatest challenge today of malignant melanoma is treatment of metastatic malignant melanoma,^983,984^ in which nanoparticle material is promising. Similar to NPs application of other tumors, one of the most crucial strategies of malignant melanoma treatment is tackle the TME. Pu et al.^981^ designed a NP-based oxidative stress amplifier named APAP@PEG/HMnO_2_ to enhance the response of oxidative stress for melanoma therapy. Tyrosinase, over-expressed in malignant melanoma cells, is considered as a special target that can activate the anti-melanoma prodrugs. In that case, APAP@PEG/HMnO_2_ is modified to be triggered by tyrosinase so that the non-toxic prodrug acetaminophen (APAP) can be precisely escorted to the position of malignant melanoma. After the accumulation of this NPs, amount of oxygen will be generated by HMnO_2_ to interfere the TME at the same time as APAP is released to produce cytotoxic benzoquinone metabolites (AOBQ), which can induce the generation of reactive oxygen species (ROS) and lead to tumor cells apoptosis. A kind of RBC membrane-cloaked NPs called R-RBC@BPtI^280^ and an inorganic material NPs named Au@Se-R/A NCs^985^ can also accelerate the intracellular ROS overproduction to attack TME and treat malignant melanoma. In addition to the method of ROS, assistant position of PTT or PDT,^977,986–989^ immune therapy^280,990–993^ and gene-related therapy^994,995^ of NPs are promising in the treatment of metastatic MM as well, even though advanced trials are required.

Although the widely recognized treatment for NMSCs is surgery, many patients have contraindications of operation. Since the lesions of NMSCs cannot be removed easily, the efficacy of surgery largely depends on the individual facts especially the surgical technique. Besides, during the surgery, some normal tissues will be removed as well, which will enlarge the scar and even lead to the psycho-social problems of patients. Photodynamic therapy (PDT) and photothermal therapy (PTT) are both regarded as relatively more stable, precise and efficient therapies than surgery,^996,967^ which can cooperate with nanomedicine technology in treatment of cSCC.^997–1000^ Most of the nanomaterial in treatment of skin cancer is for positon or imaging of PDT or PTT. According to Wang et al.^1001^, aminolevulinic acid-loaded PLGA NP (ALA-PLGA NP) can carry 5-ALA, an add-on substance can induce the protoporphyrin IX production in cSCC. This effective and targeting method provides a promising strategy in PDT of cSCC. Beside the application of orientation of phototherapy, NPs can also be applied in the topical drug delivery for skin cancer as well.^1002–1004^ He et al. designed hybrid peptide-based nanoplatform named Lupbin which is multiple-targeting and can easily penetrate the cell membrane, can carry drugs in cytosol and nucleus to attack tumor cells.^1005^

## Nanomedicine in other cancers

### Diagnosis

Thyroid cancer can be categorized into two groups by cellular origin: (1) follicular epithelial cell derived cancers; (2) parafollicular cell (C cell) derived cancer. Follicular derived cancers consist of papillary, follicular and anaplastic cancers, while C cell-derived cancer is mainly medullar type. Thyroid cancers vary in terms of incidence and lethality, driving the differential diagnosis especially important. Generally, in the past few decades, the incidence of thyroid cancer has been gradually climbing while the mortality rate has been decreasing steadily in most countries.^1006,1007^ This is partly due to the ever-improving rate of diagnosis and treatment, enabling much earlier detection and better management of the thyroid cancer. Over 95% of thyroid cancers are differentiated cancers (mainly papillary thyroid cancer and follicular thyroid cancer) in which optimal over prognosis are observed.^1008^ However, high aggressiveness can be seen in some rarer thyroid cancers like anaplastic thyroid cancer (ATC), which is poorly differentiated, highly metastatic and lacking in normal functions of normal follicular cells, for example, taking in iodine.^1009^ Patients show fast-growing neck mass and symptoms like hoarseness, dysphagia and dyspnea and few of them can survive over 6 months upon being diagnosed.^1008,1009^ Genetical and chromosomal changes can be observed in thyroid cancer cells. Abnormal changes of mitogen-activated protein kinase (MAPK) pathway are believed to be a major indicator for differentiated thyroid cancers (DTC), while RET proto-oncogene mutations for medullary thyroid cancer (MTC) and chromosomal abnormalities along with several mutations such as TP53 and CTNNB1 for ATC.^1008–1011^ Diagnostic researches of thyroid cancer have been long focused on non-invasive diagnosis. Gold, platinum and iron oxide NPs have been reported to be fabricated as novel diagnostic tools for thyroid malignancies. Omer et al. designed a kind of gold nanobiosensor in the sol-gel/PEG matrix to detect the calcitonin, which is secreted by neuroendocrine C cells, functioning as an excellent biomarker for early diagnosing MTC.^1012^ By assessing calcitonin in serum, the method offered a convenient possibility of MTC early diagnosis as well as evaluating therapeutic effects of treatment. A biosensor made by Choi et al., combined platinum NPs with TSH detection antibody-conjugated horseradish peroxidase.^1013^ With high sensitivity of assessing TSH level in the serum, they were able to detect a 100-fold lower level compared to the enzyme immunoassay on the market. Contrast agents combined with ligands showed better biocompatibility and targeting ability.^1014,1015^ Fanfone et al. improved the contrast agent for MRI, by conjugating selected peptides to the vectorized iron oxide NPs.^1015^ In vitro experiments showed ideal toxicity and binding specificity with DTC cells, making it a possible candidate as imaging probes in diagnosing thyroid cancer.

Breast cancer is a prevalent malignancy among women, with an increasing prevalence.^1,1016^ Breast cancer is characterized by the uncontrolled division and proliferation of cells in the breast tissue. Breast cancer contains various forms, which are determined by the origin within breast tissue and the extent of its spread. These varieties include ductal carcinoma in situ, invasive ductal carcinoma, lobular carcinoma in situ, and invasive lobular carcinoma. Breast cancer exhibits various molecular subtypes, including Luminal A, Luminal B, HER2 positive, and triple negative.^1017,1018^ Nanobiosensors have demonstrated great potential in the detection of a variety of biomarkers, circulating tumor cells, and other unique biomolecules in diagnosis of breast cancer.^1019–1023^ MicroRNA miR-99a-5p dysregulation has been identified as a reliable indicator of early breast cancer. Garrido-Cano et al. demonstrated a novel nanoporous anodic alumina-based biosensor for breast cancer diagnostics with great sensitivity and selectivity for detecting plasma miR-99a-5p.^1024^

### Treatment

Treatment strategies normally differ among all subtypes in thyroid cancers. Standard treatment for DTC is usually effective with surgery (lobectomy, total thyroidectomy, with or without lymph node dissection), followed by TSH suppression with L-T or radioactive iodine.^1025–1027^ However, problems are hiding behind the radioactive therapy and TSH suppression. The purpose of using radioactive iodine is to clear the residual cancer cells in the body after surgery. However, it can increase the systemic exposure to the radioactive agent as well as the chance of second primary malignant tumors.^1028–1030^ Although the strategy of TSH suppression has been proven effective in reducing the recurrence of DTC, the adverse effects on cardiovascular and skeletal systems as well as the potential of increasing the risk of malignant tumors in other systems cannot be neglected.^1025,1031–1033^ Treatment for MTC is generally surgery followed by observation, and metastatic MTC is managed by kinase inhibitors. For example, vandetanib and cabozantinib also have the limitations on efficacy.^1034,1035^ Surgical interventions, external radiation therapy and chemoradiation therapy are considered for ATC, yet its high histomorphological abnormalities and biological behaviors promote the management as a challenging work.^1009,1036,1037^ Here, recent advances of nanomedicines utilized in thyroid cancer treatment are focused and discussed.

To improve the therapeutic effects and reduce systemic toxicity of normal treatments, several kinds of nanomedicines have been designed and applied to treat thyroid cancers, including exosomes,^261,1038^ liposomes,^1039^ inorganic NPs (silicon,^883,1040–1042^ carbon,^1043^ gold,^1044,1045^ silver^1046,1047^) and so on. These nanomedicines exhibited many therapeutic priorities compared with the conventional treating protocols, especially in anaplastic thyroid cancer which poses the greatest obstacle in all thyroid cancers. Several progress regarding ATC treatment has been made in the past few years, mainly achieved by precise photothermal ablation improved by the NP modifications,^1048^ by combining nanotube-delivered drugs and shock waves to increase the intracellular drug accumulation,^1049^ by targeting vascular endothelial growth factor receptor and increase NP accumulation,^883,1042,1048^ by targeted nanoplatform delivery of RNAi agents,^1050^ and by targeting cancer cells using NK cell-derived extracellular particles.^1043^ All those explorations offer us new ideas of conquering the barriers of ATC treatment.

Nanomedicines can also be helpful assistants for the surgery. Carbon NPs (CNs) were reported to be lymph node tracers in thyroid microcarcinoma surgeries for the purpose of showing lymph nodes and preserving parathyroid glands. Results suggest CNs could be an ideal choice for the aim of tracing and preservation, for it helps retrieve more lymph nodes of metastasis, reduces unwanted parathyroid gland removal, and leads to a shorter recovery duration.^1051–1062^ However, the significance of the improvement remains controversial. Some insignificant reported negative results in the comparison of lymph nodes removed and parathyroid glands preserved,^1063^ while some reported insignificant changes of hypocalcemia incidence (confirmed by a meta-analysis in 2017)^1062,1064–1066^ nor number of metastatic lymph nodes detected.^1065^ Since a huge amount of new results are being reported during these years, a new and more comprehensive meta-analyses are in urgent demand to solve the controversy.

The current treatment strategies for breast cancer encompass surgery, chemotherapy, radiotherapy, hormone therapy, and immunotherapy, with adjuvant systemic therapy always required. Nanomedicines are emerging as promising breast cancer therapeutic agents that could correct drugs defects and ensure precise drug distribution.^1067–1071^ Zhang et al. created the PPD self-assembling dendrimer nanosystem, which was coated with an anti-PD-L1 antibody and used to deliver a small interfering RNA (siRNA) to target PDK1. PPD significantly inhibits both PDK1-induced glycolysis and the PD-1/PD-L1 pathway-related immune response, resulting in strong reduction of tumor development and metastasis in tumor-bearing mice models with no discernible harm.^1072^ A promising method for treating breast cancer non-invasively is photothermal therapy (PTT). Nanoparticles with unique properties for penetrating and targeting tumor tissues enhance the efficacy of PTT and have recently attracted increased interest of researchers.^1019,1073–1075^ During anti-tumor nanotherapy, it is frequently discovered that therapeutic NPs have trouble penetrating into the tumor tissue, resulting in disappointing treatment outcomes. The study conducted by Wang et al. provided evidence that the tumor vascular basement membrane, in addition to the endothelial barrier, functioned as a significant mechanical obstacle which firmly trapped NPs within the subendothelial space. In an experimental model of breast cancer in mice, researchers successfully broke the basement membrane barrier and enhanced the efflux of NPs by applying local hyperthermia therapy.^1076^

## Prospect and challenges

### Prospects

Nanomedicine is an emerging area with rapid developments, and being regarded as a promising tactic in solving problems in the diagnosis and treatment of cancer. Cancer cells, especially for solid tumors often highly express some receptors or ligands for the substance-transport and recognition among cells. Scientists can design corresponding targeting strategies based on understanding the intrinsic bio-characteristics of tumors. Nanoparticles designed to target tumor cells can precisely detect malignant tumors in the body, facilitating early diagnosis and accurate localization of cancer. This offers healthcare practitioners more accurate data, which helps in creating customized treatment plans and allows for the implementation of integrated diagnosis and treatment strategies. Nanotechnology’s exceptional performance enables the loading of several materials and the integration of diagnostic and treatment. Therapeutic medications and contrast agents can both be encapsulated in nanoparticles simultaneously to achieve imaging diagnostic and visual therapy effects.

With the developments of material science and computation technology, the nanovector for loading the aimed drugs is also emerging in endlessly, to satisfy the demands of delivering small-molecule-based chemotherapeutic drugs, genes and antibodies, etc. The nano-complex can reach a long-circulation and arrive the tumor site in a positive or passive manner. By integrating the drug carrier with nanoparticles, accurate drug delivery to the tumor site can be achieved, enhancing the medication’s local concentration and minimizing adverse effects on healthy tissues. Cancers are often with specific tumor microenvironment that generates obvious variation in biomarkers, which can be used as the trigger for promoting the drug-release of the nanocarriers upon reaching the tumor lesions. Sophisticated design can normally achieve a controlled drug-release kinetics to favor the need in precise tumor therapy at the aimed time and position. With all the efforts shown above, a fine therapeutic efficacy can be achieved with lower side-effects. Nanotechnology can also be used in photothermal therapy and immunotherapy by using nano materials to treat tumor cells with heat or stimulate the immune system to eliminate malignant cells.

The heterogeneity of tumors should always be considered during the therapeutic procedures in clinic. We should also pay attention to the necessity of pharmacokinetics and toxicology analysis of nanomaterials in vivo, and further optimize the pharmacokinetic effects and strengthen the therapeutic effects. However, the clinical approaches normally display hysteretic effects in combining therapies and diagnoses, which could bring about over-dose of drugs and unexpected unnecessary effects. The real-time distribution of the drugs in vivo is also difficult to be monitored upon being administrated intravenously. Nanotechnology-based vectors, to some extent, are emerging as promising tactics in providing theraonostic information in a spatial-tempo way.

### Challenges

It is generally agreed that the tumor microenvironment (TME) accounts for the disappointing results of nanomedicine therapy.^185,1077–1082^ TME, including malignant cells, tumor-associated fibroblasts (CAFs or TAFs), certain kinds of immune cells, and stroma (vessels and extravascular matrix), plays an indispensable role in the medicine resistance of cancer.^1083^ Martin et al.^1077^ hypothesized that the TME appears to limit drug delivery and immune cell migration through three key steps: immune cell/drug accumulation, distribution, and function. Three key mechanisms implement these effects: abnormal angiogenesis, desmoplasia, and hypoxia. In this case, the “renormalization or reeducation” of the TME, including the normalization of the vasculature to improve perfusion and the mediation of CAFs to reduce the level of ECM (extracellular matrix), is a possible and accessible strategy to optimize the design of nanomedicine, even if the whole mechanism of chemotherapy and immunotherapy resistance is relatively complicated.

Although the active targeting of nano drugs shows great potential in cancer, the clinical application of nano drugs is still unhurried. Firstly, due to safety issues in preclinical and clinical studies, the approval rate of emerging nano-drugs is less than 10%, and biosafety issues have attracted increasing attention.^1067,1083–1086^ It is commonly recognized that the oxidative stress that follow the production of reactive oxygen species, as well as inflammation account for the toxicity of nanoparticles. In vivo, NPs will go through four stages: absorption, distribution, metabolism, elimination. The metabolism and breakdown of NPs is mainly done in the liver and kidneys. It is generally believed that the excretion of NPs primarily occurs through the kidneys in the form of urine, and through the biliary duct in the form of feces.^302^ Nonetheless, some studies have indicated that some nanoparticles might not break down or be eliminated, and inadequate elimination may cause the NPs to remain in the body for a long time.^303,1087,1088^ Accumulation of nanocarriers in normal organs could induce physical damage, which refers to the cell damage caused by NPs through the physical obstruction of microcirculation, which triggers a series of toxic reactions, such as cell dysfunction or inflammatory reaction. The physical damage caused by the nanocarrier is mostly due to the nanocarrier’s inability to biodegrade and its ability to aggregate and move freely in bodily tissues, resulting in long-term physical harm.^1089–1093^ Therefore, the development of fine biodegradable and biocompatible nano-drug carriers is the main direction of nanomaterials research at present.

Secondly, the difficulty in finding an appropriate pre-clinical research model that truly represents the human condition and the lack of validated analytical methods to characterize nanoparticles are also important factors that hinder the development of nano-drugs.^726,1094–1096^ The development of favorable biodegradable and biocompatible nano-drug carriers, the establishment of standardized analytical methods to characterize nanoparticles, and the construction of methods to monitor the safety and efficacy of nano drugs, as well as the understanding of structure-and-property relationship of the nanomedicines will be major challenges in the future. At the same time, with the development of new biological materials and nanotechnology, more effective and safe nanomaterials will be more designed for targeted drug delivery to cancer.

The clinical translation in nanomedicine should always be considered as the ultimate goal. This requests the scientists to seriously tailoring the building materials, size, surficial property, charge in the design, the drug loading/encapsulating efficiency, drug distribution, as well as metabolism and excretion of the vectors during applications.^1096,1097^ During the clinical translation phase, nanomedicines face various challenges, especially related to the establishment of pharmacokinetic models, the formulation of nanomedicines, and the evaluation of their biological properties. The rational design of physicochemical properties of nanomedicine is critical in allowing nanomedicine immune evasion, tumor extravasation and diffusion, cellular targeting and internalization, and controlled release of therapeutic drugs. Nevertheless, the systematic simultaneous evaluation of the numerous properties of NPs continues to pose difficulties due to the inherent challenge of synthesizing NP libraries with distinct characteristics in a rapid, accurate, and reproducible manner.^1098^ Actually, the biocompatibility is currently regarded as one of the bottlenecks in clinical translation. Most nanotechnology-based drug delivery systems can achieve favorable results in the cell and animal models, however, more challenges are still facing upon the clinical II–IV trials as human bodies vary greatly from the animal models.^1099^ A classic example is the so-called EPR effect, which is normally observed in animal models and support the mechanism of most nanosystems in accumulating into the tumors. However, few evidences have been found in clinical research about EPR effect in human bodies. These phenomena request us to exploit new and reliable evidences, theory and techniques in developing drug delivery systems.

In summary, employing nanomedicine in treating cancers are still in the ascendant, since nanotechnology possesses multiple advantages and many of which can fit the requirements in tumor treatment. Ideally, the therapeutic nanotechnology-based vectors should be able to specifically deliver the loaded cargos into the tumor, after which the vectors should be bio-degraded with no side effects. Numerous works, including the principle-establishment, material-design, animal model development and the deep understanding of tumors’ biological characteristics, should be undertaken in better utilizing nanotechnology in battling cancers in a more efficient way.
